# Single molecule real-time sequencing of *Xanthomonas oryzae* genomes reveals a dynamic structure and complex TAL (transcription activator-like) effector gene relationships

**DOI:** 10.1099/mgen.0.000032

**Published:** 2015-10-30

**Authors:** Nicholas J. Booher, Sara C. D. Carpenter, Robert P. Sebra, Li Wang, Steven L. Salzberg, Jan E. Leach, Adam J. Bogdanove

**Affiliations:** ^1^​ Plant Pathology and Plant-Microbe Biology Section, School of Integrative Plant Science, Cornell University, Ithaca, NY 14853, USA; ^2^​ Icahn Institute for Genomics and Multiscale Biology and Department of Genetics & Genomic Sciences, Icahn School of Medicine at Mount Sinai, New York, NY 10029, USA; ^3^​ Departments of Biomedical Engineering, Computer Science, and Biostatistics and Center for Computational Biology, Johns Hopkins University, Baltimore, MD 21205, USA; ^4^​ Bioagricultural Sciences and Pest Management, Colorado State University, Fort Collins, CO 80523, USA

**Keywords:** PacBio, single molecule real-time sequencing, SMRT, TAL effectors, whole gene assembly, *Xanthomonas*

## Abstract

Pathogen-injected, direct transcriptional activators of host genes, TAL (transcription activator-like) effectors play determinative roles in plant diseases caused by *Xanthomonas* spp. A large domain of nearly identical, 33–35 aa repeats in each protein mediates DNA recognition. This modularity makes TAL effectors customizable and thus important also in biotechnology. However, the repeats render TAL effector (*tal*) genes nearly impossible to assemble using next-generation, short reads. Here, we demonstrate that long-read, single molecule real-time (SMRT) sequencing solves this problem. Taking an ensemble approach to first generate local, *tal* gene contigs, we correctly assembled *de novo* the genomes of two strains of the rice pathogen *X. oryzae* completed previously using the Sanger method and even identified errors in those references. Sequencing two more strains revealed a dynamic genome structure and a striking plasticity in *tal* gene content. Our results pave the way for population-level studies to inform resistance breeding, improve biotechnology and probe TAL effector evolution.

## Data Summary

1. The PacBio assembly for BLS256 has been deposited in GenBank as an update to the original accession number CP003057.1 (http://www.ncbi.nlm.nih.gov/nuccore/CP003057.1).
2. The PacBio assembly for PXO99A has been deposited in GenBank as an update to the original accession number CP000967.1 (http://www.ncbi.nlm.nih.gov/nuccore/CP000967.1).
3. The raw sequence data for the BLS256 assembly have been deposited in the Sequence Read Archive under accession number SRX502906 (http://www.ncbi.nlm.nih.gov/sra/SRX502906).
4. The raw sequence data for the PXO99A assembly have been deposited in the Sequence Read Archive under accession number SRX502899 (http://www.ncbi.nlm.nih.gov/sra/SRX502899).
5. The assembly for CFBP7342 has been deposited in GenBank under accession number CP007221.1 (http://www.ncbi.nlm.nih.gov/nuccore/CP007221.1).
6. The assembly for PXO86 has been deposited in GenBank under accession number CP007166.1 (http://www.ncbi.nlm.nih.gov/nuccore/CP007166.1).
7. The raw sequence data for the CFBP7342 assembly have been deposited in the Sequence Read Archive under accession number SRX502893 (http://www.ncbi.nlm.nih.gov/sra/SRX502893).
8. The raw sequence data for the PXO86 assembly have been deposited in the Sequence Read Archive under accession number SRX463048 (http://www.ncbi.nlm.nih.gov/sra/SRX463048).
9. The raw RS I sequence data for PXO99A-L have been deposited in the Sequence Read Archive under accession number SRX1053794 (http://www.ncbi.nlm.nih.gov/sra/SRX1053794).
10. The raw RS I sequence data for PXO99 have been deposited in the Sequence Read Archive under accession number SRX1053696 (http://www.ncbi.nlm.nih.gov/sra/SRX1053696).
11. Raw data for any of the above as bas.h5/bax.h5 files are available from the authors.
12. The pbx toolkit for assembly of *tal* genes and extraction of encoded RVD sequences is available on GitHub (https://github.com/boglab/pbx).

## Impact Statement

This study describes a moderately high-throughput approach to accurately determine whole-genome sequences of an important group of plant-pathogenic bacteria that deploy proteins known as TAL (transcription activator-like) effectors during infection, and presents an analysis of four such genome sequences and the TAL effectors they encode. Due to their remarkably complex repetitive structure, TAL effector-encoding DNA sequences are not captured by commonly used high-throughput methods, so this new approach is an enabling advance. The analysis revealed a dynamic overall genome structure and marked plasticity of the TAL effector-encoding sequences, illustrating the strong adaptive potential of these bacteria. As TAL effectors play determinative roles in many plant diseases and are important as customizable DNA-binding proteins, the ability to inventory them across populations, and the insights gained in this initial study, will foster more rapid identification of key host targets, the development of durable disease resistance, better understanding of host–pathogen evolution and improvements in biotechnology.

## Introduction

*Xanthomonas* is a large genus of plant-associated bacteria. Many species are important pathogens, and they reduce quality and yield in plants we depend on for food, feed, fibre and ornamentation. Not all, but many *Xanthomonas* species deploy DNA-binding proteins called TAL (transcription activator-like) effectors that enter the host nucleus and directly upregulate specific host genes. Individual TAL effectors are often critical determinants of the host–pathogen interaction, boosting expression of disease susceptibility (*S*) genes important for infection and symptom development or activating a resistance (*R*) gene that orchestrates a localized host cell death and limits infection (Bogdanove *et al.*, 2010). The target specificity of each TAL effector is conferred by a central domain [the central repeat region (CRR)] displaying tandem repeats of a 33–35 aa sequence (Herbers *et al.*, 1992). The repeats create a superhelical structure that wraps around the DNA, aligning contiguously with individual nucleotides on one strand of the DNA through characterized, base-specific interactions at residue 13 (Deng *et al.*, 2012; Mak *et al.*, 2012). The number of repeats and the amino acids at residue 13 across the repeats can therefore be used to predict the length and nucleotide sequence of the target (Boch *et al.*, 2009; Moscou & Bogdanove, 2009). Residue 12 also varies, typically between His and Asp, and together 12 and 13 constitute the repeat variable diresidue (RVD), with residue 12 playing an intramolecular structural role to stabilize the repeat. The simple modularity of TAL effector–DNA interaction facilitates target identification, enables the synthesis of artificial targets and allows assembly of TAL effector constructs with custom specificities. For this reason, in addition to their importance in plant disease, TAL effectors have become widely used tools for genome editing, systems biology and other DNA targeting applications (Doyle *et al.*, 2013).

**Fig. 1.**
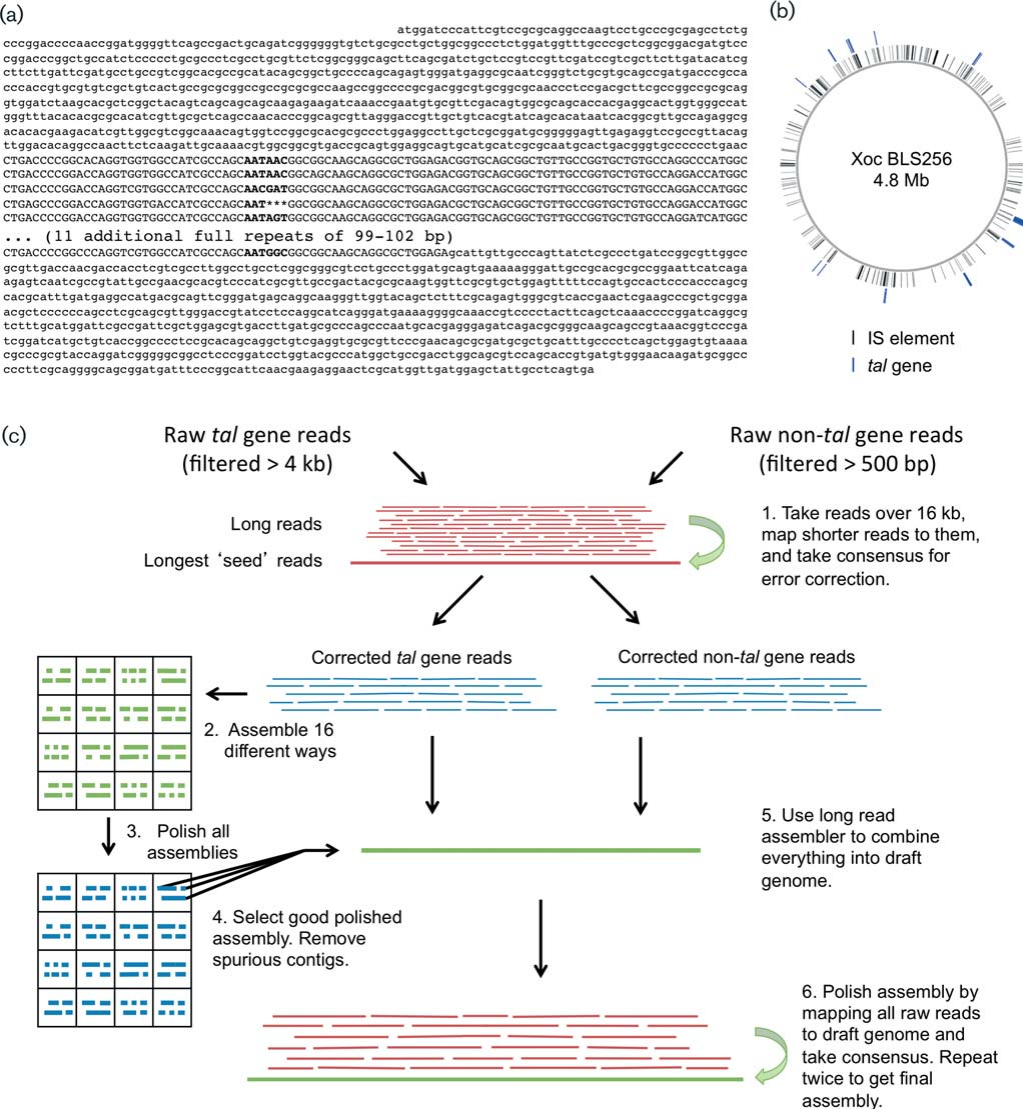
The *tal* gene-rich *Xanthomonas* whole-genome assembly challenge and our workflow. (a) Nucleotide sequence of a typical *tal* gene, i.e. *tal2g* of Xoc BLS256, illustrating the multiple, tandem, near-identical repeats (capital letters) of ∼100 bp that make accurate assembly a challenge. RVD codons are bold; asterisks represent missing bases relative to the other repeats. Eleven repeats are omitted to conserve space. Some *tal* genes have >30 repeats (Boch & Bonas, 2010). (b) A schematic of an *X. oryzae* genome (strain BLS256) indicating the 28 *tal* genes (in 12 clusters) as well as the numerous IS elements that it harbours. The characteristic abundance of each adds to the assembly challenge. The schematic was prepared using Circos (Krzywinski *et al.*, 2009). (c) Our assembly workflow. The method is based on the hgap workflow described by Chin *et al.* (2013) separately applied to reads that belong to regions containing *tal* genes and reads for the rest of the genome. For the *tal* gene regions, only reads ≥ 4 kb are used to reduce the chance of an ambiguous alignment interfering with the consensus. After assembling the *tal* gene reads and polishing the assemblies, the best assembly is chosen based on concordance with the list of RVD sequences determined by consensus across all the assemblies and representation of the most complete assembly of each *tal* gene region based on length. Specifically, the assembly is chosen that contains a *tal* gene for each consensus RVD sequence, in the fewest contigs, with the fewest or no duplicate RVD sequences. If this assembly has any spurious contigs with interior regions of no coverage, they are discarded before proceeding. Red, raw reads; green, draft assembly; blue, corrected reads or assembly. An automated workflow for the local assembly, called the ‘pbx toolkit’, is available on GitHub (https://github.com/boglab/pbx).

On average, TAL effectors comprise 17 repeats, plus a final repeat truncated at 20 aa, but some contain >30 repeats (Boch & Bonas, 2010). Apart from the number of repeats and variation at the RVD, TAL effectors are highly conserved across their entire amino acid sequences. Depending on the species and strain, individual *Xanthomonas* genomes may harbour zero to >24 TAL effector-encoding (*tal*) genes. These may be plasmid-borne or distributed in clusters around the chromosome, are typically flanked by highly conserved sequences and are often associated with insertion sequence (IS) elements, which are generally abundant in *Xanthomonas*. Not surprisingly, these features render accurate assembly of *tal* gene sequences in their genomic context challenging (Fig. 1). Indeed, available sequences to date derive exclusively from individually cloned genes and genes in the still relatively few whole *Xanthomonas* genomes assembled from Sanger sequencing reads. None of the numerous *Xanthomonas* draft genomes that have been generated using next-generation, short-read sequencing technologies contain *tal* genes, the read lengths being insufficient to accurately assemble the repeats or to distinguish which *tal* genes belong where in a genome (e.g. Bart *et al.*, 2012; see http://www.xanthomonas.org/ for a compilation).

Capturing *tal* gene sequences is nonetheless critically important. By allowing target prediction, it hastens the identification of host *S* and *R* genes (Cernadas *et al.*, 2014; Hu *et al.*, 2014; Li *et al.*, 2014; Pereira *et al.*, 2014; Strauss *et al.*, 2012) and the development of novel, more effective disease control measures (Schornack *et al.*, 2013). Also, as molecular contact points in plant–pathogen co-evolution, the sequences of TAL effectors and their targets, compared across strains and species and respective hosts, provide a basis for inferences about function based on evidence for diversifying or purifying selection. Such comparisons, especially in the genomic context, might also reveal the relative importance and frequencies of point mutations, recombination and horizontal transfer in the evolution of new TAL effector specificities within and across populations, which would inform estimates of durability of any TAL effector-oriented disease resistance mechanism. Not least, additional TAL effector sequences promise to reveal structural variation that may be useful in fine-tuning specificity or other behaviour of engineered TAL effector proteins.

In this study, we assessed the efficacy of single molecule, real-time (SMRT) sequencing (Pacific Biosciences) (Eid *et al.*, 2009), hereafter ‘PacBio’ sequencing, for *Xanthomonas* whole-genome assembly and comparative analysis of *tal* gene content. PacBio sequencing on an RS II machine using the latest P6-C4 chemistry yields ∼55 000 long reads per reaction unit (‘SMRT cell’) with a mean read length of 10 000 bp and maximum read length >60 000 bp. The technology has a high error rate for the raw output, approaching 15 %, but the lack of coverage bias and the non-systematic distribution of errors enable correction algorithms like Quiver to achieve accuracy >99.999 % by taking consensus sequences across reads (Chin *et al.*, 2013). These properties allow PacBio sequencing to be used for *de novo* assembly of microbial genomes to finished quality (Koren *et al.*, 2013), targeted sequencing of repetitive elements (Guo *et al.*, 2014), structural variant discovery (English *et al.*, 2014) and methylation motif finding (Flusberg *et al.*, 2010).

Our strategy was to first resequence with PacBio technology the genomes of two *Xanthomonas* strains we completely sequenced previously by the Sanger method, i.e. the rice pathogens *X. oryzae* pv. *oryzicola* (Xoc) strain BLS256 (Bogdanove *et al.*, 2011) and *X. oryzae* pv. *oryzae* (Xoo) strain PXO99A (Salzberg *et al.*, 2008), and to evaluate the results relative to the originals, hereafter referred to as the ‘reference’ genomes. We used stored aliquots of the original DNA preparations used for the Sanger sequencing. Assembly was *de novo*, without use of the reference genomes. Next, we PacBio sequenced two more strains (not previously sequenced) that were of interest for comparison with BLS256 and PXO99A, i.e. Xoc strain CFBP7342 and Xoo strain PXO86. Xoc strains cause bacterial leaf streak of rice and Xoo strains cause the distinct disease bacterial blight of rice (Niño-Liu *et al.*, 2006).

Like many strains of *X. oryzae*, BLS256 and PXO99A have large numbers of *tal* genes (28 and 19, respectively), more than any of the other completely sequenced *Xanthomonas* genomes, and along with Xoo strains MAFF311018 and KACC10331, the greatest and most diverse IS element content (Bogdanove *et al.*, 2011; Lee *et al.*, 2005; Ochiai *et al.*, 2005; Salzberg *et al.*, 2008). BLS256 was isolated in the Philippines in 1984 (by C. M. Vera-Cruz; C. M. Vera-Cruz, personal communication). PXO99A was derived in 1991 (Choi & Leach, 1994b) from Philippines race 6 isolate PXO99, which was collected in 1980 (by C. M. Vera-Cruz; Mew *et al.*, 1992). PXO99A was selected on media containing 5-azacytidine and is more readily transformed than its progenitor, presumably due to mutation in one or more restriction modification systems (Choi & Leach, 1994b).

CFBP7342 was isolated (by V. Verdier) in 2009 in Burkina Faso from a wild rice species; it is highly virulent and exhibits a distinct *tal* gene RFLP profile from BLS256, and like other African Xoc strains, it groups distinctly from Asian strains based on MLST (Wonni *et al.*, 2014). PXO86 is a Philippines race 2 isolate, collected in 1977 (by C. M. Vera-Cruz; Vera Cruz *et al.*, 1984), that grouped more closely than PXO99 with other strains from the Philippines (PXO99 showed greater similarity in RFLP analyses to isolates from Nepal and India) (Adhikari *et al.*, 1995). PXO86 is the source of two of the earliest known and best-studied TAL effector genes: *avrXa10* (Hopkins *et al.*, 1992) and *avrXa7* (Yang *et al.*, 2000). A third *tal* gene from this strain, *aB4.5*, although less well characterized, has also been cloned and sequenced (Bai *et al.*, 2000; S. Makino, C. Younger and A. J. Bogdanove, unpublished).

## Methods

### DNA isolation

The BLS256 and PXO99A DNA used for Sanger sequencing and subsequently for PacBio sequencing, and the CFBP7342 and PXO86 DNA used for PacBio sequencing was prepared using a protocol for total genomic DNA isolation modified from a previous protocol (Ausubel *et al.*, 1994), as follows. Bacteria were cultured overnight in 30 ml glucose yeast extract media (2 % glucose, 1 % yeast extract) in a 250 ml flask at 28 °C on a rotary shaker at 250 r.p.m., harvested by centrifugation at 3000 ***g*** for 10 min at 4 °C, then gently resuspended and washed in 20 ml NE buffer (0.15 M NaCl, 50 mM EDTA) twice, and also at 3000 ***g*** for 10 min at 4 °C to remove extracellular polysaccharide. Cells were then gently resuspended in 2.5 ml sterile 50 mM Tris, pH 8.0, 50 mM EDTA, and then 0.5 ml 25 mM Tris, pH 8.0, 10 μl ReadyLyse (Epicentre) and 50 μl RNase (10 mg ml^− 1^) added. Suspensions were mixed gently by inversion and then incubated on ice for 45 min. Following incubation on ice, 1.0 ml STEP buffer (0.5 % SDS, 50 mM Tris, pH 7.5, 40 mM EDTA, protease K at 2 mg ml^− 1^) was added, and the lysate was mixed well by inversion and incubated at 37 °C for 1 h, mixing every 10–15 min. Next, 1.8 ml 7.5 M ammonium acetate was added and the lysates mixed rapidly by hand, then subjected to extraction with phenol/chloroform (10 ml) twice and chloroform/isoamyl alcohol (24 : 1, pH 8.0, 10 ml) once, shaking vigorously by hand to mix, and separating the aqueous and organic phases by centrifugation at 7000 ***g*** for 10 min at 4 °C. Following this, the aqueous phase was transferred to a 14 ml tube and DNA precipitated by addition of 2 vols cold 95 % ethanol and gentle, repeated inversion. Once solidified, the DNA was transferred to a 2 ml microcentrifuge tube using a Pasteur pipette with the tip previously sealed and bent into a hook over a flame. Remaining liquid was then removed by centrifugation at 2000 ***g*** for 5 min, the pellet washed once with 70 % ethanol, remaining liquid removed as before and the tube left open to dry in a laminar flow hood until the edges of the pellet became glossy in appearance (10–15 min). Finally, the pellet was dissolved in 100 μl TE buffer (10 mM Tris/HCl, 1 mM EDTA, pH 8.0) overnight at 4 °C and then adjusted to a concentration of 1 μg μl^− 1^.

To ascertain the presence or absence of small plasmids, DNA was prepared and examined by agarose gel electrophoresis as described previously (Chakrabarty *et al.*, 2010).

For the PCR assay (below), for sequenced isolates the DNA prepared for sequencing was used; for other isolates, cells were harvested by centrifugation from overnight liquid cultures, washed twice with sterile water and DNA isolated using a QIAamp DNA Mini kit (Qiagen).

### PCR assays

To assay for the presence of the 212 kb tandem duplication, forward primer T679RIGHT (5′-AGAACCTGTTCACGATCTCCTGAGC-3′) and reverse primer T679LEFT2 (5′-TTGGGGATTCGTGATTGGAGATGG-3′) (Salzberg *et al.*, 2008) were used to amplify 1154 bp across the duplication junction. As a control, forward primer B1275 (5′-GCCTGGAAAGACAGCCTGAT-3′), which anneals 5′ of the tandem duplication in the reference genome, and T679LEFT2 were used to amplify 1657 bp spanning the 5′ border. Amplification was carried out with an initial denaturation at 95 °C for 3 min, and 35 cycles of 30 s at 95 °C, 30 s at 58 °C and 2 min at 72 °C, with a final elongation step of 5 min at 72 °C.

To amplify *tal7b* and *tal8b*, primers B1281 (5′-GTCCGAAGAACGCAATACGC-3′) and B1282 (5′-GACCTTGGAGAGCACGTTCA-3′), which anneal outside of the coding sequence, were used. Amplification was carried out using a touchdown PCR protocol with an initial denaturation at 95 °C for 3 min, and 26 cycles of 30 s at 95 °C, 30 s at 68–55 °C, stepping down by 0.5 °C each cycle, and 4.5 min at 72 °C, and then 10 cycles of 30 s at 95 °C, 30 s at 55 °C and 4.5 min at 72 °C, with a final elongation step of 10 min at 72 °C.

All reactions were carried out using DreamTaq polymerase (Thermo Fisher Scientific) and using ∼3–5 ng template DNA.

### PacBio sequencing

DNA library preparation and sequencing was performed according to the manufacturer's instructions. In short, 3–7 μg extracted, high-quality, genomic DNA from each isolate was verified using Qubit analysis to quantify the mass of dsDNA present. After quantification, each sample was diluted to 150 μl using Qiagen elution buffer at 33 μg μl^− 1^. The 150 μl aliquots were individually pipetted into the top chambers of Covaris G-tube spin columns and sheared gently for 60 s at 4500 r.p.m. using an Eppendorf 5424 benchtop centrifuge. Once complete, the spin columns were flipped after verifying that all DNA was now in the lower chamber. Then, the column was spun for another 60 s at 4500 r.p.m. to further shear the DNA and place the aliquot back into the upper chamber, resulting in a ∼20 000 bp DNA shear, verified using a DNA 12000 Agilent Bioanalyzer gel chip (Agilent). The sheared DNA isolates were then repurified using a 0.45 ×  AMPure XP purification step (0.45 ×  AMPure beads added, by volume, to each DNA sample dissolved in 200 μl EB, vortexed for 10 min at 2000 r.p.m., followed by two washes with 70 % alcohol and finally diluted in EB). This AMPure XP purification step assures removal of any small fragment and/or biological contaminant.

After purification, ∼3.5 μg of each of the purified and sheared samples were taken into DNA damage and end repair. Briefly, the DNA fragments were repaired using DNA Damage Repair solution (1 ×  DNA Damage Repair Buffer, 1 ×  NAD^+^, 1 mM ATP high, 0.1 mM dNTP and 1 ×  DNA Damage Repair Mix) with a volume of 21.1 μl and incubated at 37 °C for 20 min. DNA ends were repaired next by adding 1 ×  End Repair Mix to the solution, which was incubated at 25 °C for 5 min, followed by the second 0.45 ×  Ampure XP purification step. Next, 0.75 μM Blunt Adaptor was added to the DNA, followed by 1 ×  template Prep Buffer, 0.05 mM ATP low and T4 ligase at 0.75 U μl^− 1^ to ligate (final volume of 47.5 μl) the SMRTbell adapters to the DNA fragments. This solution was incubated at 25 °C overnight, followed by a 65 °C, 10 min ligase denaturation step. After ligation, the library was treated with an exonuclease cocktail to remove unligated DNA fragments using a solution of 1.81 U Exo III 18 μl^− 1^ and 0.18 U Exo VII μl^− 1^, and then incubated at 37 °C for 1 h. Two additional 0.45 ×  Ampure XP purifications steps were performed to remove < 2000 bp DNA and organic contaminants.

Upon completion of library construction, samples were validated as ∼20 kb using another Agilent DNA 12000 gel chip. All isolate libraries were sufficient for additional size selection to remove any SMRTbells < 7000 bp. This step was conducted using Sage Science Blue Pippin 0.75 % agarose cassettes to select library in the range of 7000–50 000 bp. This selection is necessary to narrow the library distribution and maximize the SMRTbell subread-length for the best *de novo* assembly possible. Without selection, smaller 2000–7000 bp molecules will dominate the zero-mode waveguide loading distribution, decreasing the subread-length. Also note that any plasmids < 7000 bp will not be seen in the size-selected sequencing data, but this was validated as discussed previously. Between 11 and 23 % of the input libraries eluted from the agarose cassette and was available for sequencing. For all cases, this yield was sufficient to proceed to primer annealing and DNA sequencing on the PacBio RS II machine. Size selection was confirmed by Bio-Analysis and the mass was quantified using the aforementioned Qubit assay.

Primer was then annealed to the size-selected SMRTbell with the full-length libraries (80 °C for 2 min 30 s followed by decreasing the temperature by 0.1 °C s^− 1^ to 25 °C). The polymerase–template complex was then bound to the P5 enzyme using a ratio of 10 : 1 polymerase to SMRTbell at 0.5 nM for 4 h at 30 °C and then held at 4 °C until ready for magbead loading, prior to sequencing. The magnetic bead-loading step was conducted at 4 °C for 60 min per manufacturer's guidelines. The magbead-loaded, polymerase-bound, SMRTbell libraries were placed onto the RS II machine at a sequencing concentration of 75 pM and configured for a 180 min continuous sequencing run.

Sequencing was conducted to >150 ×  coverage by using four to six SMRT cells per strain. Two cells each for CFBP7342 and PXO86 used the P4-C2 sequencing enzyme and chemistry combination, whilst the rest used XL-C2. In all datasets, read-length distribution showed a fat tail, with 20 % of coverage after adaptor removal contained in subreads ≥ 15 000 bp. Downstream analyses were designed for this distribution and may not work well for lower quality datasets. The technology has subsequently improved such that, at the time of this writing, equivalent coverage can be obtained using one or two SMRT cells.

### Assembly overview

As our initial attempts at whole-genome assembly using the hgap assembler (Chin *et al.*, 2013) included in the PacBio software package SMRTAnalysis 2.0 failed for BLS256 (see Results), we took the approach of first carrying out local assemblies of reads containing *tal* genes and then using those assemblies to seed the whole-genome assembly (Fig. 1 and following sections), which was successful. During the course of our study, an upgrade to the hgap software was released (hgap 3.0) that resolved the BLS256 genome without local *tal* gene assembly. However, we found subsequently that for some genomes, the local *tal* gene assembly was still required and we recommend it regardless for validation of any assembly. The data and analysis presented in this paper are based on the assemblies made using the hgap assembler in SMRTAnalysis 2.0 combined with our local *tal* gene assembly toolkit. Details of the results of assembly using hgap 3.0 are presented in the Discussion.

### Assembly of *tal* gene sequences

For each strain, a list of raw reads for *tal* gene regions was generated by using blasr (Chaisson & Tesler, 2012) to align reads to the BLS256 *tal* gene sequences, following the PacBio hgap Whitelisting protocol (PacBio, 2013a). Next, a modification of the RS_PreAssembler protocol included in SMRTAnalysis 2.0 was run on these reads. In this modification, which we designated the RS_PreAssembler_TALs protocol, the ‘whiteList’ parameter for the filtering step was set to the *tal* gene read list. The minimum read-length cut-off was set to 4000, the seed read-length cut-off was set to 16 000 to ensure that short-read to long-read alignments used for correction would be long enough to be unambiguous and the maxLCPLength was set to 14, as recommended for data using the XL-C2 enzyme and chemistry (PacBio, 2013b). Specifically, the blasr options string was changed to ‘-minReadLength 4000 -maxScore -1000 -bestn 24 -maxLCPLength 14 -nCandidates 24’.

After preassembly, corrected reads were trimmed to estimated QV50 windows and filtered to those ≥ 4000 bp using the SMRTAnalysis 2.0 trimFastqByQVWindow.py utility. Based on comparison with the reference genomes, these reads are typically 97 % accurate. Reads were assembled using the Minimo assembler of amos 3.1.0 (Treangen *et al.*, 2011), using NUCmer 3.1 (Kurtz *et al.*, 2004) for the overlap step, for all 16 combinations of a 500, 1000, 2000 and 3000 minimum overlap length, and 91, 93, 95 and 97 minimum overlap per cent identity. Contig sets generated by each of these assemblies were polished separately with the RS_Resequencing protocol included in SMRTAnalysis 2.0. This protocol aligns reads to the assembled regions and uses the Quiver algorithm to call the consensus, regularly achieving 99.999 % accuracy in regions with ≥ 60 ×  coverage (Chin *et al.*, 2013). For this, read filtering settings were set to those used for preassembly, the ‘Place Repeats Randomly’ option was unchecked and all other settings were left at defaults.

RVD sequences were determined from the 16 polished *tal* gene assemblies using a consensus approach. For each contig across all polished assemblies, encoded TAL effector CRRs were extracted and split into RVD sequences by conserved boundaries. Inspecting a sorted list of unique RVD sequences and the number of times they were encountered in the 16 assemblies (e.g. File S1, available in the online Supplementary Material), sequences ending in frameshifts or other anomalies that were prefixes of other sequences that occurred more often were discarded. The resulting list was retained as the correct RVD sequences. As an additional measure in case any *tal* genes were incompletely assembled before polishing, assemblies of the polished contigs in each set were carried out, again with Minimo, and the RVD sequence consensus process repeated. In all cases the results were identical.

This workflow for assembly of *tal* genes and extraction of encoded RVD sequences, which we have named the pbx toolkit, is automated and available on GitHub (https://github.com/boglab/pbx). The only required input is the path to a folder containing bas.h5 and bax.h5 files of raw sequence reads. Additional options allow specifying the sequences to use for identifying *tal* gene reads and the conserved repeat boundaries to use for RVD sequence determination. This enables the workflow to be easily adapted for use with other *Xanthomonas* genomes.

### Whole-genome assembly

To choose a *tal* gene assembly to seed the whole-genome assembly, first, polished assemblies were identified that contained a *tal* gene for each consensus RVD sequence, in the fewest contigs, with few or no duplicate RVD sequences. Coverage graphs for these assemblies that were generated during the RS_Resequencing protocol were then inspected to find the assembly with no or the fewest spurious contigs (peak coverage < 10 or interior areas of no coverage). This assembly, with any spurious contigs removed, was chosen to anchor the whole-genome assembly as described below. For BLS256, this was the 1000 bp overlap, 91 % identity assembly, for PXO99A the 3000 bp overlap, 93 % identity assembly, for PXO86 the 3000 bp overlap, 97 % identity assembly and for CFBP7342 the 3000 bp overlap, 95 % identity assembly.

For the PXO99A assembly, the FASTQ file of the sequences in the chosen *tal* gene assembly was combined with the trimmed, error-corrected *tal* gene reads and the trimmed, error-corrected non-*tal* gene reads for assembly using the Celera assembler (version 7.0; SVN revision 4334) (Myers *et al.*, 2000). Prior to assembly, the contig for the short version of *tal7b* and the corrected reads that produced it were removed. For the BLS256, PXO86 and CFBP7342 assemblies, one of the Minimo assemblies done during RVD sequence determination with the chosen *tal* gene assembly merged a few additional regions and this assembly was combined with the error-corrected read sets instead. For BLS256 this was the 2000 bp overlap, 97 % identity assembly, for PXO86 the 3000 bp overlap, 97 % identity assembly and for CFBP7342 the 3000 bp overlap, 95 % identity assembly.

To choose overlap settings for Celera, the assembler was run through the unitigger stage for all 16 combinations of a 500, 1000, 2000 and 3000 minimum overlap length (set by the ovlMinLen setting), and 94, 95, 96 and 97 minimum overlap per cent identity (set by the utgErrorRate and utgGraphErrorRate settings). Other parameters used were unitigger = bogart, ovlErrorRate = 0.06, utgErrorLimit = 4.5 cnsErrorRate = 0.25 and cgwErrorRate = 0.25, which are the defaults used by hgap. The Celera documentation (http://wgs-assembler.sourceforge.net/wiki/index.php/RunCA) details the effects of these settings. For each overlapper run, a visualization of the best read-overlap graph was generated as described (Chin, 2014). A stringent overlap settings combination that still produced a circular or linear overlap graph with a single connected component was chosen; for all strains this was the 1000 bp minimum overlap, 97 % overlap identity combination. Celera was then run again until the end, using the new overlap settings combination and the parameters just described. For all strains except BLS256, the resulting assembly was a single contig or a single large contig with a few smaller contigs. In all cases where smaller contigs were present, the smaller contigs were regions already represented in the larger contig and these small contigs were discarded. For BLS256, multiple contigs resulted, however, these had sufficient overlap for subsequent assembly using Minimo.

To determine whether the key to resolving the BLS256 genome when using SMRTAnalysis 2.0 was the local *tal* gene assembly or adjusting the Celera parameters, we took the corrected read set for BLS256 that had been used for an hgap assembly and reassembled it using Celera across the different parameter configurations. This parameter sweep produced assemblies with five, seven or nine contigs, indicating that the separate, local assembly of *tal* gene reads was the key.

To finish the full assemblies, the RS_Resequencing protocol from SMRTAnalysis 2.0 was run with the default settings, except for the ‘Place Repeats Randomly’ setting, which was unchecked. The resulting consensus sequence was then run through the RS_Resequencing protocol again with the same settings. The consensus sequence of this run was then circularized by splitting it in half at an arbitrary location away from any *tal* gene region and assembling the fragments with Minimo. If the fragments failed to combine back into a single contig due to non-overlapping ends, error-corrected long reads from the whole-genome assembly read set that Celera placed at the edges of the original contig were identified and added to Minimo. In all cases this enabled circularization. The assembly for each strain was then rotated and flipped to match the start position and strand of the start of the most closely related reference sequence: BLS256 for CFBP7342 and PXO99A for PXO86. The RS_Resequencing protocol was then run again with the earlier settings to produce the final assembly.

### Additional finishing step for PXO86 and CFBP7342

The 5′ ends of some *tal* genes have a homopolymer of nine guanines. The initial finished assemblies for PXO86 and CFBP7342 each had frameshifts in this area in a few cases. Homopolymers of this length are a known weak point of the Quiver consensus algorithm used by the RS_Resequencing protocol (Alexander, 2013). The newer P4-C2 chemistry used by two of the SMRT cells each for PXO86 and CFBP7342 achieves higher consensus accuracy using Quiver with P4-C2 tuned parameters than earlier chemistries using the C2 tuned parameters included in SMRTAnalysis 2.0 (Alexander, 2013). Therefore, to improve accuracy in these homopolymer regions, SMRTAnalysis 2.2 was installed and we reran the RS_Resequencing on the finished assembly of each strain using only the P4-C2 SMRT cell data. This eliminated both frameshifts at this spot in PXO86, and all but one in CFBP7342 (*tal11g*), and these updated sequences are the ones submitted to GenBank.

### Validation of assemblies

The PBHoney structural variant finder (part of PBSuite 14.7.14) was run for all finished assemblies to identify possible structural variants or mis-assemblies. We considered only the ouput of the ‘tails’ method. Reads were mapped with blasr using the settings string ‘-nCandidates 15 -sdpTupleSize 6 -minPctIdentity 75 -affineAlign’ as recommended by the PBHoney README. The ‘tails’ program was run with settings ‘-B 1000 -b 6 -z 6 -v -q 1’.

### Data access

The PacBio assemblies for BLS256 and PXO99A have been deposited in GenBank as updates to the original accessions, numbers CP003057.1 and CP000967.1, respectively. The raw sequence data for these assemblies have been deposited in the Sequence Read Archive under accession numbers SRX502906 and SRX502899, respectively. The assemblies for CFBP7342 and PXO86 have been deposited in GenBank under accession numbers CP007221.1 and CP007166.1, respectively. The raw sequence data for these assemblies have been deposited in the Sequence Read Archive under accession numbers SRX502893 and SRX463048, respectively. Raw data from the PXO99A-L and PXO99 RS I sequencing runs have been deposited the Sequence Read Archive under accession numbers SRX1053794 and SRX1053696, respectively. Raw data as bas.h5/bax.h5 files for any of these are available from the authors.

## Results

### Accurate capture of *tal* genes by localized assembly

To assess the ability of PacBio sequencing to accurately capture *tal* genes in *X. oryzae*, we obtained ∼200 ×  coverage in PacBio continuous long reads using 20 kb libraries constructed from stored aliquots of the genomic DNAs originally isolated for the BLS256 and PXO99A Sanger reference genomes. Using the hgap assembler (Chin *et al.*, 2013) included in SMRTAnalysis 2.0, we obtained a complete, gap-free assembly for PXO99A, but only fragmented assemblies for BLS256, none with fewer than seven contigs and all with breakpoints within *tal* gene regions. We therefore attempted local assembly of just the *tal* gene regions using a custom assembly pipeline that combines components of the hgap workflow with the Minimo (Treangen *et al.*, 2011) assembler (Fig. 1). Using this approach for either genome, we were able not only to assemble all *tal* genes, but to identify and correct errors and omissions in those genes in the respective reference genomes (see below).

### Correction of a frameshift within *tal2b* of BLS256

*tal2b* is one of eight TAL effector genes in the *tal2* locus of BLS256, two of which are labelled as pseudogenes in the reference assembly: *tal2b* because of a 1 bp insertion causing a frameshift within the CRR, and *tal2h* because of large deletions in the 5′ and 3′ ends of the coding sequence. After assembling the *tal* genes in BLS256 using our pipeline, we found that whilst the *tal2h* deletions are well supported by the PacBio data, the polished assemblies do not display a frameshift in *tal2b*. To investigate, we revisited the reference assembly in the NCBI Trace Archive and found that there are four reads for this location: two with wide, unresolved peaks that were interpreted by the base caller as containing an extra base, causing the frameshift, but two others with sharply defined peaks displaying no extra base (File S2). We conclude that the frameshift insertion is an error in the reference assembly due to incorrect base calling.

### Discovery in PXO99A of a version of *tal7b* or *tal8b* with an internal deletion

**Fig. 2.**
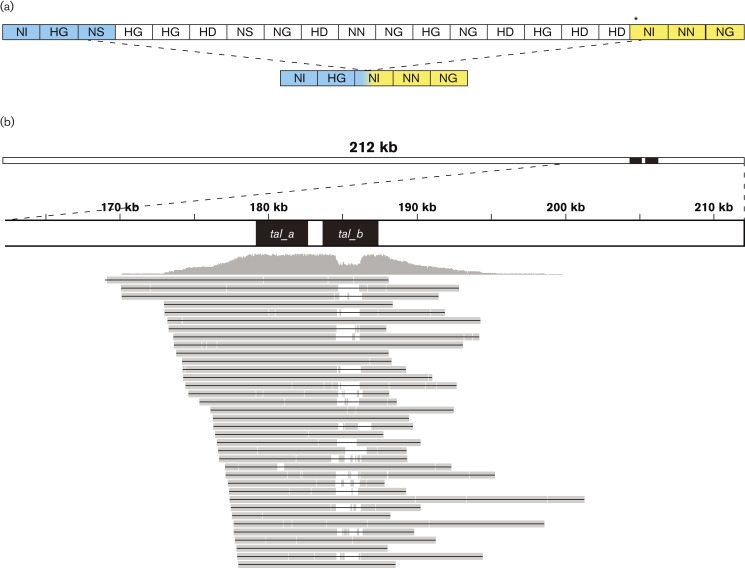
An allele of *tal7b* or *tal8b* of PXO99A with a deletion in the central repeat region. (a) Schematic of the central repeat regions of the full-length gene (top) and the short allele showing the RVDs encoded by each repeat. The RVDs of the short allele match the first two and last three RVDs of the full-length gene. However, whilst the third-from-last repeat of the full-length gene uniquely encodes a leucine at position 3 (asterisk), that repeat of the short allele encodes the standard proline. Therefore, the short allele was likely created by a deletion within the full-length gene resulting from recombination (dotted lines) between the third repeat and the third-from-last repeat of the full-length gene, after codon 3 and before codon 13 of those repeats (colour gradient). (b) A representative selection of the ∼60 PXO99A-s PacBio reads >10 kb that span the *tal* locus within the 212 kb duplication. The schematic at top shows the duplicated DNA collapsed to single copy with an enlarged view (dotted lines) of the last 50 kb, containing the *tal* locus. The two *tal* genes, *tal7a* and *tal7b* or *tal8a* and *tal8b*, are indicated by filled rectangles labelled *tal_a* and *tal_b*. Shown below is a plot of the number of the reads that align at each position and, below that, schematics showing the alignment for each read. Gaps (white space) in roughly half the alignments correspond to the internal deletion at *tal7b* or *tal8b*. Reads were aligned using blasr (Chaisson & Tesler, 2012) and the alignments displayed using igv (Robinson *et al.*, 2011; Thorvaldsdóttir *et al.*, 2013).

The PXO99A reference genome contains a remarkable (for its size) 212 087 bp, near-perfect, tandem duplication, in which the two copies of the 212 087 bp sequence differ by only a single base pair within an IS element. The duplication includes a locus with two *tal* genes, *tal7a* and *tal7b* in the first copy, and *tal8a* and *tal8b* in the second. *tal7a* (and the identical *tal8a*) has 18 repeats (including the truncated last repeat) in its CRR and *tal7b* (and its duplicate, *tal8b*) has 20. In the localized assembly of the PacBio data for all of the *tal* genes in PXO99A, we discovered a contig spanning the *tal* locus within the duplication but displaying in place of *tal7b* (or *tal8b*) a *tal* gene with only five repeats in its CRR. The encoded RVDs of the gene match the first two and last three encoded RVDs of *tal7b* (and *tal8b*) (Fig. 2). However, the third to last repeat of *tal7b* (and of *tal8b*) encodes a leucine at position 3 that is unique to that repeat within the gene, whilst the third repeat of the gene with five repeats encodes the standard proline at that position, suggesting that the five-repeat gene resulted from a deletion within *tal7b* or *tal8b* caused by recombination between the third repeats from each end (Fig. 2).

To confirm the internal deletion, we first checked the NCBI Trace Archive for the reference assembly. As each internal *tal* gene repeat is ∼100 bp, five repeats are short enough to be spanned by an 800 bp Sanger read and indeed we found 10 reads in the Trace Archive that each align across the entire repeat region of the five-repeat gene. Next, we aligned the raw PacBio reads to the *tal* gene sequences in the PXO99A reference genome and identified 30 reads >10 kb containing a *tal* gene with approximately five repeats. When aligned to the PXO99A reference genome, all of these reads mapped unambiguously to the *tal* gene region within the 212 kb duplicated sequence, with a gap in the CRR of *tal7b* (or *tal8b*) consistent with the internal deletion (Fig. 2). This coverage is similar to what we observed for the junction of the duplication itself (36 reads >10 kb; see below). Finally, we carried out Southern blot analysis of the PXO99A DNA using a *tal* gene-specific probe and observed a band corresponding exactly to the five-repeat gene (File S3).

The deletion confirmed, we next tried to determine whether it occurs in *tal7b* or in *tal8b*. As the distance from *tal7b* or *tal8b* to the end of the duplicated 212 kb sequence is ∼24 kb and our dataset contains nearly 750 subreads >24 kb in length, we checked whether there were any subreads that would span the distance and reveal which copy of the duplication the deletion is in. One 27 kb subread reaches from 1 kb into the second copy of the 212 kb sequence back to *tal7b*, but falls just short of the CRR of the gene. We were therefore unable to determine from the sequence data alone whether the deletion is there or at *tal8b*.

### Whole-genome assemblies for BLS256 and PXO99A

Returning to the BLS256 PacBio dataset, we ran the hgap pre-processing protocol on the non-*tal* reads to generate a set of error-corrected long reads, of mean length 18 163 bp, for the rest of the genome. We reasoned that if we combined these reads with the error-corrected *tal* gene reads, and then added the high-quality *tal* gene region assemblies from our combined hgap and Minimo pipeline, the assembler would be better equipped to assemble the entire genome. Using this nested assembly method, we obtained a single contig for the entire BLS256 genome, which after post-processing differs from the reference in only 13 bases, all indels, one of which is the *tal2b* frameshift correction described earlier. (Note that 13 differences out of 4.8 Mbp is 99.9997 % agreement.)

Whilst the hgap assembler was already able to produce a single contig assembly for PXO99A, we wanted to check whether the method we developed for BLS256, an Xoc genome, could also be applied to Xoo genomes such as PXO99A. After removing the contig for the truncated copy of *tal7b* we captured during localized assembly, and the reads that produced it, we were able to generate an assembly for PXO99A that differs from the reference by 10 bases, all indels. Both the automated protocol and our method collapsed the 212 kb tandem duplication into a single copy.

A schematic of our overall assembly pipeline is given in Fig. 1. We have made an automated workflow for the local assembly, called the ‘pbx toolkit’, available on GitHub (https://github.com/boglab/pbx). The PacBio assemblies for BLS256 and PXO99A have been deposited in GenBank as updates to the original accession numbers NC_017267 and NC_010717.

### Evolution of *tal* gene content captured *in vitro*: segregation of *tal7b* and *tal8b* with reversion of the PXO99A 212 kb duplication

**Fig. 3.**
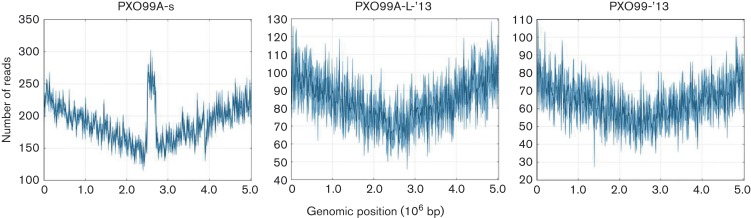
Coverage across the PXO99A reference genome from PacBio sequencing of PXO99A-s, PXO99A-L-’13 and PXO99-’13 DNAs. For each dataset, PacBio read count (*y*-axis) is plotted against genomic position (*x*-axis) of the Sanger sequenced genome (Salzberg *et al.*, 2008) with the 212 000 bp tandem repeat collapsed. Plots were generated by SMRTAnalysis 2.2 with number of regions set to 500.

The PacBio data support the presence of the 212 kb tandem duplication in the accession of PXO99A represented by the Sanger reference sequence, hereafter referred to as accession PXO99A-s. When all reads are mapped to the reference, 89 reads span the duplication junction and 36 of these are >10 kb. When all reads are mapped to the reference genome with the duplication collapsed into one copy, that sequence has nearly twice as much coverage as the surrounding area (Fig. 3).

We previously noted that the near-perfect identity of the PXO99A-s duplication suggests a very recent origin (Salzberg *et al.*, 2008). The accession PXO99A-s was made by author A. J. B. in 2005 from a single-colony culture of an earlier PXO99A accession, provided by F. White (now at the University of Florida). The culture was split to make a stock for the accession and to prepare the genomic DNA used for the original Sanger sequencing and for the subsequent PacBio sequencing presented here. The original PXO99A accession, predating the White accession, was made in 1991 by author J. E. L. from a single colony grown under 5-azacytidine selection from her accession of the field isolate PXO99. To specify the original PXO99A accession, we refer to it herein as PXO99A-L. Prior to our attempts at complete genome sequencing using PacBio technology, we had carried out two PacBio RS I sequencing runs each to compare PXO99 and PXO99A-L draft sequences and methylation patterns. We took advantage of those data to probe the origin of the duplication. For the RS I sequencing, DNA had been prepared from a single-colony culture of each accession grown in March 2013. These DNA preparations are hereafter specified by the suffix ‘-’13'. After mapping the reads to the reference genome with the duplication collapsed, we saw no elevated coverage for that region in the PXO99-‘13 or the PXO99A-L-’13 data (Fig. 3). Further, mapping to the full reference genome, we found no reads spanning the duplication junction.

**Fig. 4.**
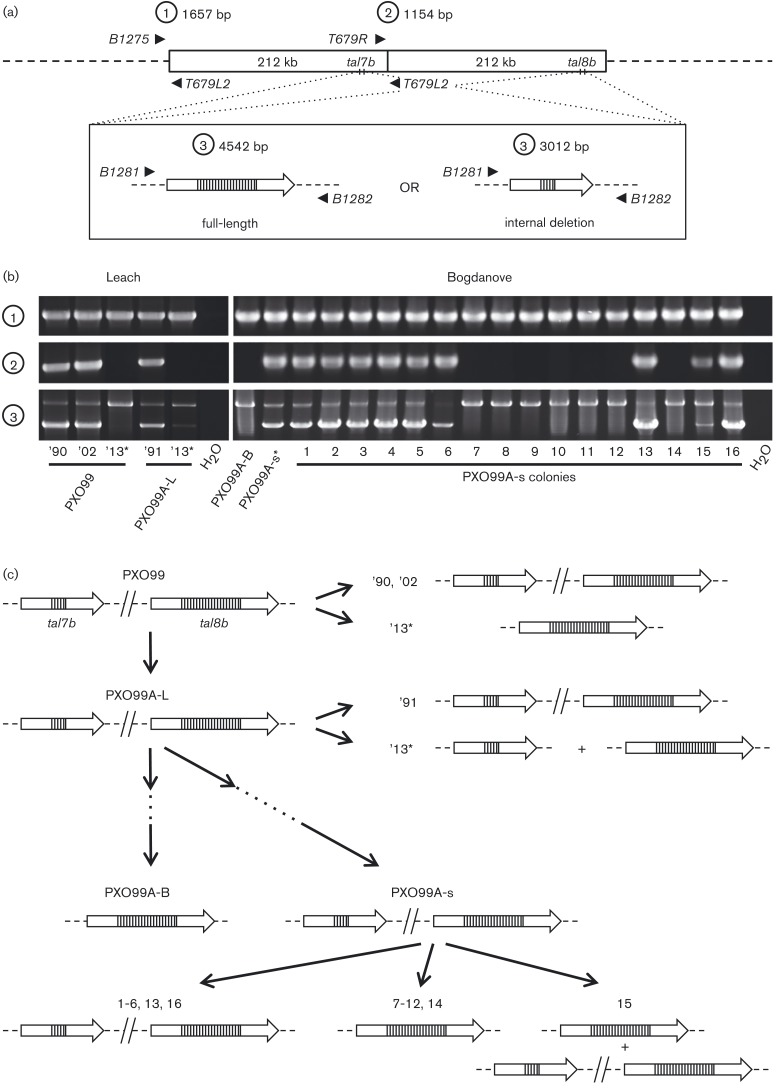
Reversion of the 212 kb tandem duplication and segregation of *tal7b* and *tal8b*. (a) Schematic of (top) the 212 kb duplication in the PXO99A reference genome (corresponding to the accession PXO99A-s) showing the location of the *tal7b* and *tal8b* genes, and (bottom) the full-length gene as well as the short allele, with an internal deletion, that was detected in the PacBio data for PXO99A-s. Circled numbers mark sets of PCR primers (labelled, black triangles) used to amplify (1) the 5′ border of the duplication with the rest of the genome, (2) the duplication junction, and (3) the *tal7b* and *tal8b* genes; the expected product sizes in each case are given in base pairs. Note that the reverse primer of set 1 and set 2 is the same. (b) Results of PCR amplification using primer sets 1, 2 and 3, and each of several PXO99- and PXO99A-derived templates. The templates are DNAs prepared in the years indicated from single-colony cultures of the original PXO99 accession and the original PXO99A accession (PXO99A-L) made in the Leach laboratory, DNA prepared in 2014 from a heavy streak of a 2002 PXO99A accession called PXO99A-B made in the Bogdanove laboratory, an aliquot of the Sanger and PacBio sequenced PXO99A-s DNA, DNAs prepared from 16 single-colony cultures grown from the PXO99A-s accession, and water as a control. Asterisks denote DNAs that were PacBio sequenced. (c) A model based on the PCR results above showing the inferred, predominant genotype of (in bold) the original PXO99 accession, the original PXO99A accession PXO99A-L, the 2002 PXO99A accession PXO99A-B and the PXO99A-s accession. Line arrows show the relationships of the accessions (from parent to derivative), with dashed arrows indicating an uncertain number of intermediate accessions. Also shown are the inferred phenotypes present in DNA prepared from single-colony cultures of PXO99, PXO99A-L and PXO99A-s, labelled by year or by colony number as in (b). Genotypes are illustrated as in (a) by one or two block arrows, representing either the short *tal7b* gene or the full-length *tal8b* gene, on a dashed horizontal line, with diagonal lines indicating presence of the duplication. The model shows *tal7b* as the short allele based on its relative infrequency in revertants (see text).

Next, we performed PCR using primers that amplify the duplication junction (Salzberg *et al.*, 2008) on samples of each of the DNAs used for PacBio sequencing, i.e. PXO99-’13, PXO99A-L-’13 and PXO99A-s. We included samples of additional, earlier preparations of PXO99 and PXO99A-L DNA, also made from single-colony cultures of the original accessions. These DNA preparations are hereafter specified, by year they were made, as, PXO99-’90, PXO99-’02 and PXO99A-L-’91. We also included DNA from a culture grown from a heavy streak of a 2002 accession of PXO99A, specified here as PXO99A-B, which was made by author A. J. B. from a single colony provided by F. White at that time. Finally, we included DNA from 16 distinct, single-colony cultures grown from the PXO99A-s accession. As a positive control for the PCR, we carried out a separate reaction for each template using the reverse primer in conjunction with a distinct forward primer corresponding to a sequence just prior to the duplication. The results, shown in Fig. 4, confirm the absence of the duplication in the sequenced PXO99-’13 and PXO99A-L-’13 DNA preparations (and its presence in the sequenced PXO99A-s DNA). Surprisingly, however, the PXO99-’90, PXO99-’02 and PXO99A-L-’91 DNA samples all show a band for the duplication junction, revealing that the duplication was present in the original PXO99 accession and persisted through the 5-azacytidine selection that gave rise to PXO99A-L, but was lost due to recombination between the two copies (Roth *et al.*, 1996) in the culturing that gave rise to PXO99A-L-’13, and likely in the culturing that gave rise to PXO99-’13 (absence from PXO99-’13 could alternatively be explained by heterogeneity in the original PXO99 accession, either from loss or gain of the duplication during creation of the accession). DNA from the 2002 PXO99A-B DNA and seven of the 16 PXO99A-s single-colony cultures also failed to yield a band for the duplication junction. Unless PXO99A-B and the PXO99A-L-’13 culture derived from a clonal subpopulation of the original PXO99A-L accession that had lost the duplication, these observations point to at least two more instances of loss, one in the culturing that led to PXO99A-B and one (or more) in the culturing that led to the seven PXO99A-s colonies.

The multiple and possibly many instances of reversion we observed among the samples described above prompted us to assay those same samples for the presence of the *tal7b* or *tal8b* internal deletion, in order to probe the origin of the deletion and attempt again to determine whether it is in *tal7b* or in *tal8b*. We carried out PCR on each sample using primers corresponding to unique sequences flanking both *tal7b* and *tal8b* (Fig. 4). Every sample that shows the duplication yielded a large band corresponding to the full-length gene and a small one corresponding to the short allele. Purification and sequencing of the smaller band (from the PXO99A-s sample) revealed a perfect match to the PacBio contig and corresponding Sanger reads that show the deletion. Thus, the deletion also dates back at least to the original PXO99 accession.

PXO99-’13, PXO99A-B and each of the seven PXO99A-s single-colony samples that likewise showed no evidence of the duplication yielded only the larger band that corresponds to the full-length gene. These samples represent at least two, probably three and possibly more independent reversions.

Interestingly, PXO99A-L-’13, despite showing no evidence for the duplication either in the PacBio data or by PCR, yielded both the large and the small bands for the *tal7b* and *tal8b* amplification. The small band is just visible, quite faint relative to the large band and to the corresponding small band in each sample that shows the duplication, which is much brighter in those samples than the accompanying large band. Sequencing of the purified small band confirmed its identity. We interpret the PCR result therefore as evidence that two distinct reversion events took place independently at early but different times during the PXO99A-L-’13 culture, leading to undetectable amounts of the original genotype and disproportionate amounts of the two revertant genotypes, with the genotype bearing the full-length gene predominant. Indeed, none of the 125 PacBio reads for PXO99A-L-’13 that overlaps the gene displays the short allele, consistent with its being barely detectable by PCR.

The results for PXO99A-s colony 15 provide evidence of yet another instance of reversion and segregation in culture, in this case, as with PXO99A-L-’13, leading to heterogeneity in the DNA sample. Bands for the duplication junction and both alleles of the *tal* gene are each present, but the junction band and the short allele band are both faint. This pattern suggests that the duplication was lost at some point to introduce a subpopulation carrying only the full-length allele.

Altogether, the PCR results for the duplication junction and the *tal* gene internal deletion identify a minimum of five reversion events leading to retention of the full-length gene and one the short allele (Fig. 4c). When the two copies of a particular gene within a duplication are different, they segregate among revertants according to their position within the duplication (Roth *et al.*, 1996). *tal7b* and *tal8b* start at position 183 654 within the duplicated 212 087 bp, so ∼87 % of recombination events leading to loss of the duplication would be expected to occur before this position and therefore retain *tal8b*, with the remaining ∼13 % retaining *tal7b*. The 5 : 1 ratio we observed almost perfectly matches this expectation and indicates that the short allele is in *tal7b*, in the first copy of the duplication. The differentiation of *tal7b* and *tal8b* that occurred with the 212 kb duplication in PXO99, and the frequency with which reversion and segregation appears to take place *in vitro* underscore the strong potential for evolution of *tal* effector gene content in the field.

All references hereafter to the PXO99A genome in comparison with other strains refer to the PXO99A-s reference genome with the 212 kb tandem duplication collapsed and bearing the full-length gene (*tal8b*), unless otherwise specified.

### Disruption of a restriction modification system explains the 5-azacytidine resistance and improved transformability of PXO99A

To investigate possible differences between PXO99 and PXO99A-L that could explain the 5-azacytidine resistance of PXO99A-L, we used Quiver to call the consensus after mapping all reads ≥ 2000 bp for each read set to the PXO99A reference genome. Whilst dnadiff (Kurtz *et al.*, 2004) reported only SNPs between the PXO99A-L consensus sequence and the reference, for PXO99 the consensus had a 40 bp deletion spanning bases 1 393 461–1 393 500. This region corresponds to a sharp drop in the read-count graphs in Fig. 3, which can be indicative of a mis-assembly. After extracting the reads for this region from the PXO99 dataset, assembling them with hgap and comparing with the reference, we found that PXO99A carries an insertion of IS element IS*Xoo13* in a gene (not annotated in the reference) that aligns with several *Not*I and *Eag*I restriction endonuclease gene homologues in the NCBI database. This gene is immediately downstream of locus PXO_04735, which encodes the likely corresponding modification enzyme, DNA (cytosine-5)-methyltransferase PliMCI. Inactivation of the restriction endonuclease gene could explain the improved transformability of PXO99A relative to PXO99 that was the objective of the 5-azacytidine selection.

### Whole-genome assemblies of CFBP7342 and PXO86

Moving to the genomes previously not sequenced, as with BLS256 and PXO99A-s we generated ∼200 ×  coverage in PacBio reads for CFBP7342 and PXO86, and assembled the data following the same ensemble approach.

**Fig. 5.**
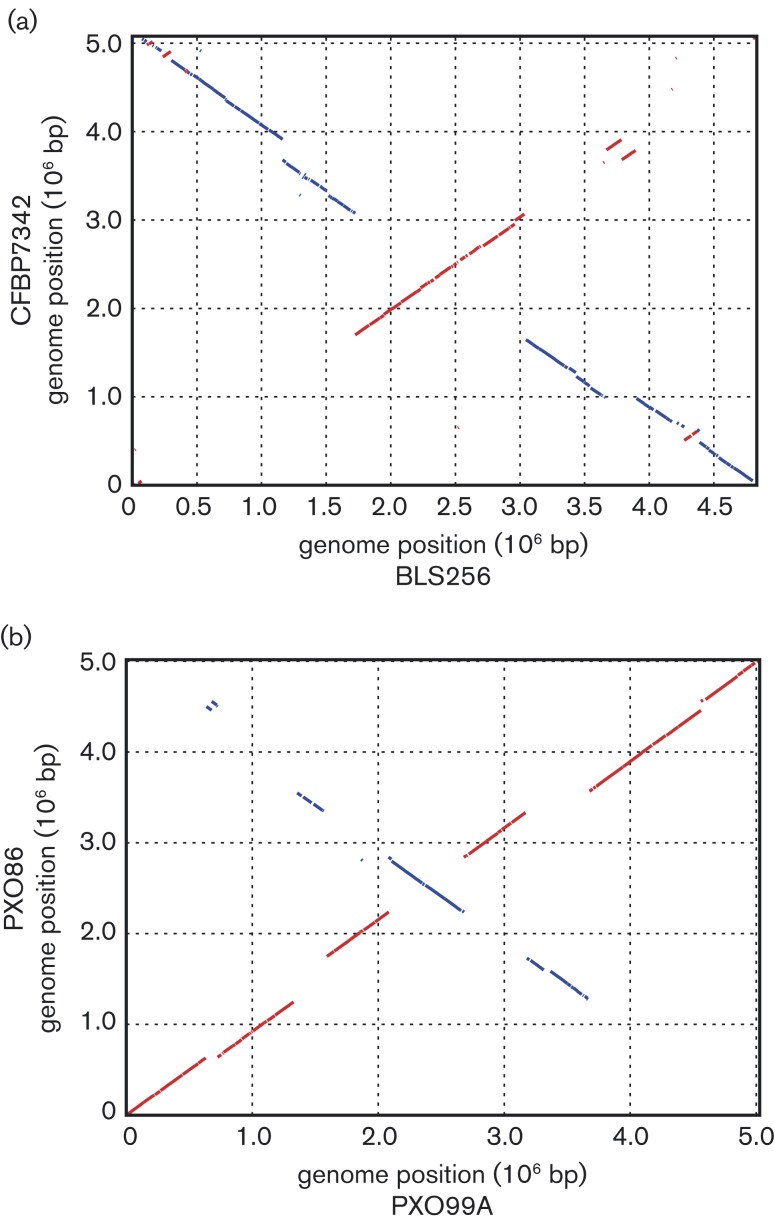
Alignments of the Xoc genomes and of the Xoo genomes. (a) The CFBP7342 genome versus the BLS256 genome. (b) The PXO86 genome versus the PXO99A genome (with the 212 kb duplication in PXO99A collapsed). In each plot, regions are coloured red if they occur on the same strand in both genomes and coloured blue if on opposite strands. Plots were created using MUMmer (Kurtz *et al.*, 2004).

The CFBP7342 genome consists of a single chromosome of 5 080 102 bp, 248 363 bp larger than the BLS256 genome. We confirmed the absence of any small plasmids that might have been excluded during size selection for the PacBio sequencing (see Methods) using the plasmid preparation protocol described by Chakrabarty *et al.* (2010), with *Xanthomonas campestris* pv. *euvesicatoria* strain 85-10 as a positive control (data not shown). A one-to-one mapping of the CFBP7342 and BLS256 genomes according to dnadiff requires 333 alignments with a mean length of 13 520 bp and a mean identity of 99.23 %. Several inversions are visible along the main diagonal in a whole-genome alignment plot (Fig. 5a). Regions in BLS256 that do not align to CFBP7342 total 143 580 bp (2.97 % of the genome); regions in CFBP7342 that do not align to BLS256 total 298 047 bp (5.87 % of the genome) and include several prophages (File S4).

The PXO86 genome is 5 016 623 bp, 11 365 bp shorter than the PXO99A genome. It also consists of a single chromosome, with no plasmids. A one-to-one mapping to the PXO99A genome requires 256 alignments with a mean length of 18 728 bp and a mean identity of 99.59 %. Only three major inversions are visible along the main diagonal in a whole-genome alignment plot (Fig. 5b). The decreased fragmentation and higher similarity of alignments of these strains compared to the Xoc alignment is consistent with the shorter geographical distance of their isolation sites. Regions in PXO99A that do not align to PXO86 total 106 613 bp (2.12 % of the genome) and include two prophages; regions in PXO86 that do not align to PXO99A total 62,326 bp (1.24 % of the genome) and include several restriction modification systems, among them the *Xor*II methyltransferase and endonuclease (Choi & Leach, 1994a) (File S5).

The PacBio assemblies for CFBP7342 and PXO86 have been deposited in GenBank under accession numbers CP007221.1 and CP007166.1.

### *tal* genes of CFBP7342

**Fig. 6.**
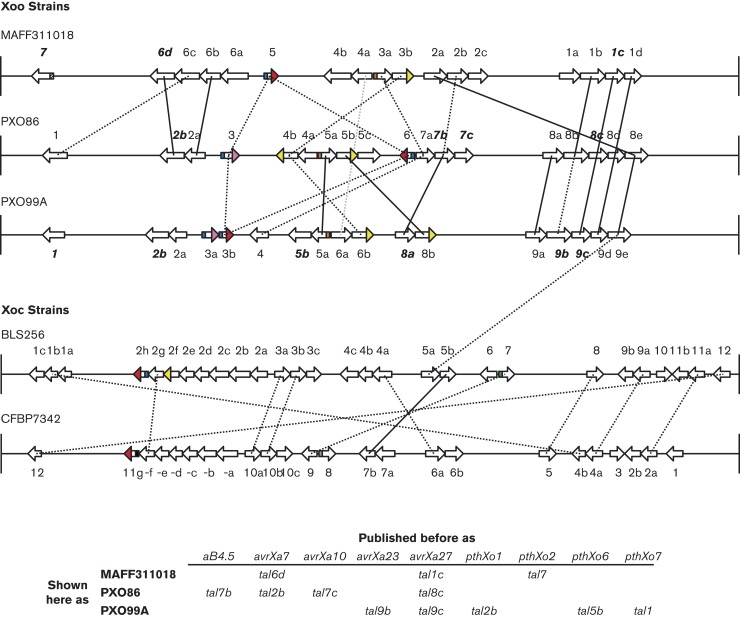
Relationships among *tal* genes in sequenced *X. oryzae* strains. Genes are represented by their coding sequences as arrows, divided into three regions: the 5′ end and CRR, which together make up the shaft of the arrow, and the 3′ end, shown as the arrowhead. The *tal* gene regions are magnified relative to the rest of the genome, but intergenic regions and *tal* gene sizes relative to each other are to scale within each strain. A solid line between two *tal* genes indicates that their CRRs encode identical RVD sequences and a dashed line indicates they encode nearly identical RVD sequences with no more than three total RVD substitutions or additional 3′ repeats in one relative to the other. The 5′ and 3′ regions are colour coded: white, typical 864 bp 5′ end and 861 bp 3′ end (Xoo) or 837 bp 3′ end (Xoc); hatched fill, one codon insertion in 5′ end (unique to MAFF311018 *tal7*); blue, multiple deletions in 5′ end; red, 129 bp 3′ end variant; orange, five-codon deletion in 5′ end; yellow, 11-codon duplication in 3′ end; pink, 837 bp 3′ end more typical of an Xoc *tal* gene, but with a premature stop codon resulting from a nucleotide substitution; green, one-codon insertion in 5′ end (unique to BLS256 *tal7*); black, transposon insertion in 5′ end (unique to CFBP7342 *tal11g*); grey, five-codon duplication in 5′ end (unique to CFBP7342 *tal8*). MAFF311018 *tal6c* has an atypical-length repeat (Richter *et al.*, 2014) not present in the closely related *tal1* gene of PXO86. Gene names follow the scheme of Salzberg *et al.* (2008). Those in bold italics have alternative names in the literature and these are given at bottom. The location of the five-codon duplication in the 5′ end of CFBP7342 *tal8* (grey) is at the same position within the 5′ end as the transposon insertion in CFBP7342 *tal11g* (black). The five-codon deletion in the 5′ end (orange) within the Xoo strains is also at this location. The 212 kb duplication in PXO99A is collapsed, showing the *tal* cluster with the full-length allele, labelled as *tal8b*. Note that the MAFF311018 genes are renumbered relative to our previous study (Salzberg *et al.*, 2008), as we now consider the gene previously named *tal3* to be the first gene (*tal2a*) of the *tal2* cluster.

CFBP7342 has 24 *tal* genes organized into 12 loci (Fig. 6). Six loci contain one gene, four loci contain two genes, one locus contains three genes and one locus contains seven genes. The genes were named according to our previously described scheme (Salzberg *et al.*, 2008).

**Fig. 7.**
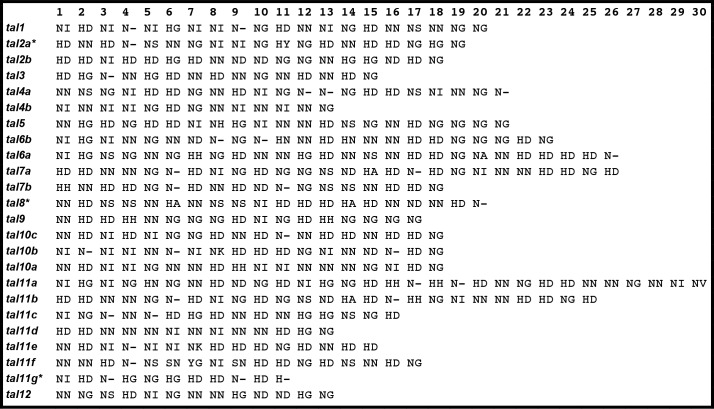
RVD sequences of TAL effectors encoded in the CFBP7342 genome. A dash indicates a missing residue 13. An asterisk indicates an unusual feature, as follows: *tal2a*, a frameshift in the 5′ end; *tal8*, a five-codon duplication in the 5′ end; *tal11g*, a frameshift followed by an IS element insertion in 5′ end and a 129 bp 3′ end.

The RVD sequences encoded in the CRRs of the CFBP7342 *tal* genes are shown in Fig. 7. None of the CRRs contains any atypical-length (‘aberrant’) repeats (Richter *et al.*, 2014). *tal7b* has an identical RVD sequence to *tal5b* of BLS256 and is the only *tal* gene of CFBP7342 identical to a BLS256 *tal* gene in this way. Several other CFBP7342 *tal* genes encode RVD sequences nearly identical to those of BLS256 *tal* genes and likely have similar targeting specificities (Fig. 6). Among these is *tal11f*, which is similar to BLS256 *tal2g*. Tal2g is a virulence factor that targets the rice bacterial leaf streak *S* gene *OsSULTR3;6* (Cernadas *et al.*, 2014). Alignment of the Tal11f RVD sequence with the *OsSULTR3;6* promoter using the same scoring function used in the Tal2g study (Doyle *et al.*, 2012) predicts that it binds at the same site as Tal2g (not shown).

CFBP7342 contains two structurally abnormal *tal* genes. The *tal8* gene contains a perfect tandem duplication of bases 244–258 (File S6). The *tal11g* gene contains a 129 bp 3′ end (the coding sequence downstream of the repeat region) resulting from a 688 bp internal deletion and later a premature stop codon (File S7). As shown in Fig. 6, *tal* genes with this feature have been detected in all strains of *X. oryzae* with finished genomes, including the Xoo strains. It is also present in several Chinese Xoc isolates (Ji *et al.*, 2014), in which, as in BLS256, it is followed by an IS element similar to IS*1403* (Lee & Chiu, 1998). Genes in which the 129 bp end occurs nearly exclusively also show multiple deletions in the 5′ end (File S8). CFBP7342 *tal11g* is an exception, having a 5′ end that is full-length but carries a frameshift mutation at the homopolymer 88 bp after the start of the gene, and an IS element 152 bp downstream of that (File S9). This IS element is identical at 1202 of 1204 positions to the IS element at the 3′ end.

### *tal* genes of PXO86

PXO86 has 18 *tal* genes organized into eight loci (Fig. 6). Three loci contain one gene, two loci contain two genes, two loci contain three genes and one locus contains five genes. Genes with other names in the literature are *tal2b* (*avrXa7*), *tal7b* (*aB4.5*) and *tal7c* (*avrXa10*). The sequences of *avrXa7* and *avrXa10* reported here differ from those reported previously (Hopkins *et al.*, 1992; Yang *et al.*, 2000), but Sanger resequencing of the original clones confirmed that those reported here are correct.

**Fig. 8.**
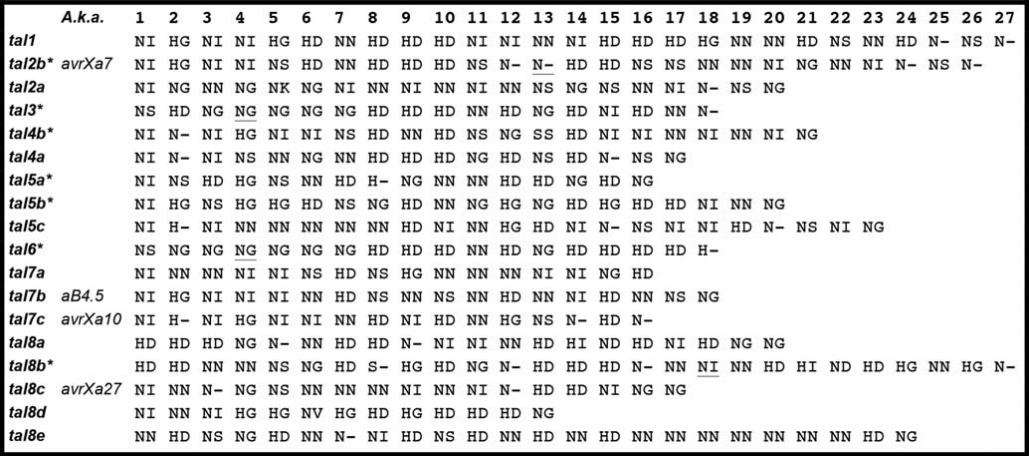
RVD sequences of TAL effectors encoded in the PXO86 genome. A dash indicates a missing residue 13. An asterisk indicates an unusual feature or additional information, as follows: *tal2b* (*avrXa7)*, repeat 13 is of atypical length (encodes 40 aa); *tal3*, multiple deletions in the 5′ end, premature stop in the 3′ end and repeat 4 is of atypical length (encodes 28 aa); *tal4b*, an 11 codon duplication in the 3′ end; *tal5a*, a five-codon deletion in 5′ end; *tal5b*, an 11-codon duplication in the 3′ end; *tal6*, multiple deletions in the 5′ end, a 129 bp 3′ end and repeat 4 is of atypical length (encodes 28 aa); *tal8b*, this gene is a nearly identical, functional allele of *avrXa23* (Wang *et al.*, 2014) and repeat 18 is of atypical length (encodes 42 aa). RVDs in repeats of atypical length are underlined. A.k.a., also known as.

The RVD sequences encoded in the CRRs of the PXO86 *tal* genes are shown in Fig. 8. Some of the CRRs include atypical-length repeats (Fig. 8). Several of the RVD sequences are identical to the RVD sequences encoded in *tal* genes in PXO99A and MAFF311018 (Fig. 6). Seven genes are shared with PXO99A and six are shared with MAFF311018. Three genes are shared across all three strains: *tal8c*, *tal8d* and *tal8e*. The *tal8c* orthologue in PXO99A is *avrXa27*. Several other PXO86 *tal* genes encode RVD sequences nearly identical to those found in other strains and likely have similar targeting specificities. Among these is *tal8b*, which is almost identical to the recently described *avrXa23* of PXO99A (Wang *et al.*, 2014).

The overall organization of the *tal* gene loci and context of the genes themselves is similar across all three Xoo strains with notable exceptions (Fig. 6). Note that throughout this report, we number the MAFF311018 *tal* genes differently from before (Salzberg *et al.*, 2008) because here we include the former *tal3* as part of the *tal2* cluster. The *tal6c* gene of MAFF311018 is part of a cluster of genes separated by a conserved spacer but is similar to *tal1* of PXO86, which occupies its own locus. *tal8e* of PXO86 is part of a cluster of five genes separated by the conserved spacer and is identical to *tal9e* of PXO99A and *tal2a* of MAFF311018; however, as previously noted (Salzberg *et al.*, 2008), *tal2a* of MAFF311018 is flanked by IS elements. Similarly, *tal7a* of PXO86, *tal4* of PXO99A and *tal3a* of MAFF311018 are all nearly identical to one another by RVD sequence, but, unlike *tal3a*, *tal7a* and *tal4* are flanked by IS elements. Also, *tal3a* of MAFF311018 has a five-codon deletion in the 5′ end, and PXO86 and PXO99A each have a *tal* gene with this feature, but it is not the one similar by RVD sequence to *tal3a*. Finally, the *tal6* locus of PXO86 is uniquely positioned among the Xoo strains, the result of a duplication of a ∼13.5 kb region around and including *tal3*.

### Complex *tal* gene relationships across strains highlighted by the 13.5 kb duplication in PXO86

The duplications containing *tal3* and *tal6* in PXO86 span base pairs 2 017 468–2 031 766 (hereafter referred to as repeat R1) and in reverse orientation 2 804 924–2 819 594 (hereafter referred to as repeat R2), respectively. R1 and R2 show 99 % identity. Both start with a copy of IS*Xo8*; however, in R2 this IS element is interrupted after 206 bp by an insertion of IS*1112b* (Ryba-White *et al.*, 2005) that is not present in R1. The similarity then continues until the 3′ end of *tal3* in R1 and *tal6* in R2. Here, *tal3* has a nucleotide substitution that causes a premature stop codon, resulting in a 552 bp 3′ end, whilst *tal6* has the 129 bp 3′ end variant (File S7). The sequence after the *tal3* stop codon continues to match a typical *tal* gene 3′ end (i.e. coding sequence), interestingly with the next in-frame stop codon at 837 bp, typical of Xoc *tal* genes. After the end of *tal3* and *tal6*, sequence similarity of R1 and R2 carries on for another few thousand bases, but the exact end of the duplication is not clear: sequence identity tapers off within a region of conserved hypothetical proteins.

Several observations suggest that the 13.5 kb duplication occurred before divergence of the lineages that produced PXO86 and PXO99A and is likely the event that gave rise to *tal3a* and *tal3b* of PXO99A. The RVD sequences of *tal3* and *tal6* in PXO86 are nearly identical to each other and to *tal3b* in PXO99A, and all have an identical, atypical-length repeat in their CRR. *tal3a* in PXO99A has 5′ and 3′ ends nearly identical to those of *tal3* of PXO86, and the atypical-length repeat, although the repeat region itself appears to be recombinant, with the atypical-length repeat shifted slightly in position relative to *tal3b* and the PXO86 genes. Also, in PXO99A, although *tal3a* and *tal3b* are clustered together, they are separated by 1936 bp rather than the 990 bp typical of other clusters, and a duplication of the region following *tal3b* is present elsewhere in the genome with 98 % identity. Incidentally, *tal5* in MAFF311018 is nearly identical throughout to *tal6* of PXO86 and is not duplicated in that genome.

As mentioned previously, the 129 bp 3′ end variant found in *tal6* is present in all strains of *X. oryzae* sequenced to date across both pathovars, whilst the 552 bp sequence occurs only in PXO86 *tal3* and PXO99A *tal3a*, which share a common origin, making it likely the 129 bp sequence is older. Based on the sequences surrounding the 129 bp 3′ end in PXO99A, this variant was speculated to have been horizontally transferred to Xoo from Xoc (Bogdanove *et al.*, 2011). The origin of the 552 bp 3′ end, however, is unclear. A search of the NCBI blast database (Zhang *et al.*, 2000) shows a perfect match to the *arp3* gene cloned from Xoo strain PXO339 (annotated as ‘putative avirulence protein Avrxa5 gene’) (Liang *et al.*, 2004), except that *arp3* does not have the premature stop codon at 552 bp. Furthermore, rather than having an Xoc-like stop codon like the additional one at 837 bp in PXO86 *tal3* and PXO99A *tal3a*, *arp3* continues until the position of the typical Xoo stop codon at 861 bp. Downstream sequence is not available, but it seems likely to be a typical Xoo spacer sequence, in contrast to the sequences downstream of PXO86 *tal3* and PXO99A *tal3a*, which as noted are more typical of Xoc *tal* genes (File S7). At the 5′ end, *arp3* shares the deletions found in PXO86 *tal3* and PXO99A *tal3a* and is identical to them elsewhere in this region at all but one position (File S8). The *arp3* gene has a markedly shorter CRR than PXO86 *tal3* and PXO99A *tal3a*. Thus, it seems likely that a recombination between an ancestor of PXO86 *tal3* and an *arp3*-like gene contributed to the differentiation of the copies of the 13.5 kb duplication.

### Prophages in CFBP7342

Using PBHoney (English *et al.*, 2014), we identified 42 reads showing structural variation at the borders of the region spanning 1 229 857–1 269 017 bp in the CFBP7342 genome. Of a mean 278 ×  coverage spanning this region, two reads show it deleted from the genome, whilst 40 reads indicate the region is circular. The annotation of CFBP7342 indicates several phage-related genes in this area and the phast (Zhou *et al.*, 2011) web tool identifies this region as an intact prophage, with an attachment site in a tRNA-Lys gene. phast reports the most similar phage to be the recently characterized Smp131 of *Stenotrophomonas maltophilia* (Lee *et al.*, 2014), with 27 genes in common. As reported in that study, Smp131 is similar to prophages identified in sequenced Xoo genomes PXO99A, MAFF311018 and KACC10331, which also have attachment sites in tRNAs. Interestingly, phast did not identify any similar prophages in PXO86 or BLS256.

PBHoney identified an additional six reads corresponding to a similar event in the region of the genome spanning 1 646 843–1 689 199 bp. Of a mean 229 ×  coverage of this region, one read shows a deletion from the genome, whilst five reads indicate the region is circular. phast also identifies this region as intact prophage, with 25 genes in common with Smp131, although based on the PBHoney result it incorrectly identifies the boundaries and attachment site. The attachment site of this prophage is in a ribosomal protein S12 methylthiotransferase gene.

phast identified three other possible intact prophages in CFBP7342. Whilst we did not find any reads supporting their replication in our dataset, all occur in regions that MUMmer (Kurtz *et al.*, 2004) cannot align to the BLS256 genome. The most likely element spans base pairs 2 955 650–2 995 168 with an attachment site in a tRNA-Val gene and has 10 genes in common with the Xfas53 phage of *Xylella fastidiosa* (Summer *et al.*, 2010). The others have no identifiable attachment site: one spans base pairs 491 860–507 360 and has five genes in common with the KS9 phage of *Burkholderia pyrrocinia* (Lynch *et al.*, 2010), and the other spans base pairs 687 159–721 147, with only a transposase in common with 10 phages in the phast database.

## Discussion

In this study, we demonstrated that PacBio sequencing is effective for generating *de novo*, whole-genome assemblies for *Xanthomonas* that accurately capture the *tal* genes in a non-cost prohibitive, moderately high-throughput way. We created an automated workflow, the pbx toolkit, that takes an ensemble approach and generates local assemblies of *tal* gene regions for integration into and or validation of the whole-genome assembly. Using this workflow, we successfully assembled *de novo* the genomes of one strain each of the two *X. oryzae* pathovars, Xoc strain BLS256 and Xoo strain PXO99A, that had been previously completed by Sanger sequencing, and corrected errors and omissions in those references. These include a base miscall that when corrected changes a *tal* pseudogene to a real gene and a previously unrecognized internal deletion in a *tal* gene located in a large tandem duplication that likely renders that copy non-functional. By PCR analysis of multiple accessions of the strain with the tandem duplication, we found that it reverts frequently, resulting in segregation of the two alleles of the *tal* gene. This *in vitro* observation illustrates the strong potential for evolution of *tal* gene content in the field. Sequencing and comparing the genomes of an additional strain of each pathovar, Xoc strain CFBP7342 and Xoo strain PXO86, we discovered further evidence of plasticity of *tal* gene content, including a highly dynamic overall genome structure and complex *tal* gene relationships within and across pathovars (Figs. 5 and 6) that suggest horizontal transfer, recombination, duplication and selection (see File S10 for a detailed discussion). In light of this plasticity, and as TAL effectors play determinative roles in many of the important plant diseases caused by *Xanthomonas* spp. and are important as customizable DNA targeting proteins, the ability to capture *tal* gene sequences in their genomic context across different strains is extraordinarily enabling. Inventory of TAL effectors across populations will aid in identifying key host targets, developing broad-spectrum and durable disease control, understanding TAL effector evolution, and probing variation to improve utility in biotechnology.

Given the determinative roles TAL effectors play as virulence and as avirulence factors, the mutability of the genes that encode them is daunting. In our study of the 212 kb duplication and the *tal7b* and *tal8b* genes, we observed *tal* genotypic shifts in the absence of any selection from a host. Under selection by an *R* gene [either a dominant, ‘executor’ type or an *S* gene variant that escapes activation (Bogdanove *et al.*, 2010)], the genetic variation potential of *X. oryzae tal* genes could be expected to result in a rapid change in pathotype (pathogen race) that overcomes that resistance. Indeed, historically, resistance-breaking populations have appeared relatively rapidly following the deployment of bacterial blight *R* genes, and in studied cases this has been shown to be due to evolution in *tal* gene content (Antony *et al.*, 2010; Mew & Vera Cruz, 1985; Vera Cruz *et al.*, 2000; R. Sundaram, G. Laha and A. J. Bogdanove, unpublished).

Strategic development and deployment of resistant germplasm requires surveying the pathotypes present. As typing pathogen isolates by using disease assays on host varieties with different *R* genes is time and labour intensive, breeders and epidemiologists have increasingly turned to molecular typing methods, such as MLST, VNTR analysis and even draft (short-read) genome sequencing (Hajri *et al.*, 2012; Mishra *et al.*, 2013; Poulin *et al.*, 2014; Triplett *et al.*, 2011; Wonni *et al.*, 2014; Zhao *et al.*, 2012). The potential for rapid evolution of new TAL effector specificities and for horizontal transfer of *tal* genes renders these approaches insufficient. Capturing *tal* gene content is essential.

Beyond typing, analysis of *tal* genes at the population level also can identify conservation and correlation pointing to specific TAL effectors as candidate virulence or avirulence factors, or to particular variants as functionally relevant. An intriguing example of the latter is the 129 bp 3′ end variant present in *tal2h* and *tal11f* of the Xoc strains BLS256 and CFBP7342, respectively, and *tal5*, *tal6* and *tal3b* of the Xoo strains MAFF311018, PXO86 and PXO99A, respectively. With the exception of *tal11f*, which has a transposon insertion in the 5′ end, the genes each display an intact ORF up to the early stop. The early stop truncates each protein prior to its activation domain. Conservation of this 3′ end variant suggests that the genes that carry it may encode functional proteins that do something other than activate a host gene. Each of the *tal* ORFs with the 3′ end variant also shows two short, internal, in-frame deletions in the 5′ end, but, as these occur downstream of the first 100 codons, they would not be expected necessarily to prevent transit of the encoded protein into host cells via the type III secretion system (Mudgett *et al.*, 2000; Szurek *et al.*, 2002). This observation and the near identity of the RVD sequences of the three truncated Xoo TAL effectors suggests that those TAL effectors indeed target a host DNA sequence. Although the RVDs are different in the truncated Xoc TAL effectors, it is important to note in this context that even TAL effectors with different specificities may target sequences in the same gene, for a similar outcome.

Typing by *tal* gene content and carrying out population-level functional and evolutionary analyses of the kind just alluded to will become increasingly feasible as improvements to the SMRT technology increase the number of reads per cell and reduce cost. Other long-read technologies that emerge, including nanopore sequencing (Oxford Nanopore Technologies) will likely also be effective and can be expected to contribute to reduced cost. A particular advantage of long-read technology even over Sanger sequencing is that most *tal* gene regions are fully spanned by one or more individual reads that also provide significant genomic context for accurate assembly. Also, the read depth we achieved with PacBio sequencing allowed us to detect structural variants and subpopulations of template DNA, such as the *tal7b* internal deletion in PXO99A and the replicating phage genomes in CFBP7342.

The already relatively low cost of PacBio sequencing enabled us to compare three accessions in the PXO99A lineage: PXO99, PXO99A-L and PXO99A-s. This comparison yielded the mutation that converted PXO99 into PXO99A, and, in combination with PCR analysis of these and several other PXO99A accessions and derivatives, revealed that the 212 kb deletion dates at least back to PXO99 and has undergone several independent reversions, which in turn enabled us to map the *tal7b* internal deletion. These cross-accession comparisons highlight the caveat that a reference genome for a strain is a working hypothesis. Recipients of PXO99 or PXO99A should note that their accession is as likely as not to lack the duplication, and if it does, it is likely but not certain to have retained the full-length *tal8b* rather than the shorter *tal7b* gene. Of course, individual accessions may differ from the reference in yet other ways. As costs of long-read technologies drop even more, one could envision routine confirmation of accession or working stock genotypes by complete genome sequencing.

During the course of our study, an upgrade to hgap (hgap 3.0) was released. hgap 3.0 uses different error-correction and consensus algorithms than hgap 2.0 from SMRTAnalysis 2.0. To assess whether hgap 3.0 improves the assembly process for *Xanthomonas* genomes, we reassembled all four datasets (PXO99A-s, PXO86, BLS256 and CFBP7342). The hgap 3.0 settings used and the assembly steps carried out are provided in File S11. Without separate local assembly of *tal* gene reads, hgap 3.0 generated complete (single contig) assemblies for BLS256, PXO86 and CFBP7342 that each differ by ≤ 10 SNPs from the corresponding assemblies we report here, each SNP being an indel and none being in a *tal* gene. For PXO99A, hgap 3.0 produced an assembly with four contigs, representing the full chromosome with the 212 kb duplication collapsed, the junction of the 212 kb duplication and two contigs for the *tal7b* or *tal8b* locus with the internal deletion. Whilst for these genomes the hgap 3.0 assemblies agreed well with those we generated using hgap 2.0 with local *tal* gene assembly using the pbx toolkit, for some genomes we have found that the results do not agree. Specifically, sequencing 10 additional Xoc genomes (Wilkins *et al.*, 2015), we found that for two hgap 3.0 partially collapsed or expanded the largest cluster of *tal* genes. This was detected because very long reads mapping to this region indicated the presence of additional or fewer *tal* genes in the cluster, respectively, matching the pbx toolkit local assembly results for those genomes. Thus, although hgap 3.0 alone may often yield correct *Xanthomonas* whole-genome assemblies, validation and, if needed, correction based on local *tal* gene assembly and inspection of available very long reads spanning *tal* gene loci is recommended. For other repeat-rich organisms, including those harbouring *tal* gene homologues, the local assembly approach would likely also be useful. The pbx toolkit could be adapted readily to evaluate hgap genome assemblies for such organisms. A template to extract reads containing such repeats for local assembly and a set of sequences for pattern matching to later extract the assembled repeat sequences for analysis would be the primary requirements.

The hgap 3.0 results and our results overall highlight the rapid improvements being made in assembly methods for PacBio and other sequence data, but also illustrate that each new method may still require empirically determined user input to close some genomes. We agree with Koren *et al.* (2014) that an ensemble approach that seeks consensus among assemblies generated using different methods increases confidence in the accuracy of the assembly. For genomes harbouring multiple *tal* genes in particular, incorporating local, subgenomic assemblies is likely to remain important. Also, although newer chemistry and Quiver parameters achieve higher consensus accuracy at long homopolymers than previously (see Methods), apparent frameshifts at such locations should be examined carefully, taking integrity of the downstream sequence into consideration in interpreting whether a frameshift is real. In all cases, we strongly advocate release of raw data in bas.h5/bax.h5 format to enable independent validation of any new genome assembly and additional analysis as new tools and methods are developed.

## Supplementary Data

78 Supplementary Data

4521 Supplementary Data

164 Supplementary Data

54 Supplementary Data

69 Supplementary Data

16 Supplementary Data

17 Supplementary Data

17 Supplementary Data

29 Supplementary Data

99 Supplementary Data

75 Supplementary Data
