# Supplementary material for: Single molecule real-time sequencing of Xanthomonas oryzae genomes reveals a dynamic structure and complex TAL (transcription activator-like) effector gene relationships
Source: Microb Genom. 2015 Oct 30;1(4):e000032. doi: 10.1099/mgen.0.000032 (PMC4853030; doi:10.1099/mgen.0.000032)
Supplement: Supplementary file 1 — Supplementary Data [file mgen-01-32-s001.pdf]

Supplementary Material for

SMRT SEQUENCING OF *XANTHOMONAS ORYZAE* GENOMES REVEALS A DYNAMIC STRUCTURE AND COMPLEX TAL EFFECTOR GENE RELATIONSHIPS

Nicholas J. Booher[1], Sara C. D. Carpenter[1], Robert P. Sebra[2], Li Wang[1], Steven L. Salzberg[3], Jan E. Leach[4], and Adam J. Bogdanove[1]*
Address: [1] Plant Pathology and Plant-Microbe Biology Section, School of Integrative Plant Science, Cornell University, Ithaca, NY 14853 USA; [2] Icahn Institute for Genomics and Multiscale Biology and Department of Genetics & Genomic Sciences, Icahn School of Medicine at Mount Sinai, New York, NY 10029 USA; [3] Departments of Biomedical Engineering, Computer Science, and Biostatistics and Center for Computational Biology, Johns Hopkins University, Baltimore, MD 21205 USA; [4] Bioagricultural Sciences and Pest Management, Colorado State University, Ft. Collins, CO 80523 USA
***Corresponding author: ajb7@cornell.edu**

File S1. Representative output from the *tal* gene RVD sequence exporter for a set of assemblies of the BLS256 *tal* genes. Sequences are listed in alphabetical order with the number of occurrences on the left to facilitate picking maximal length sequences. An exclamation point indicates a repeat of atypical size, hash indicates the exporter could not find the conserved residues flanking the RVDs, and "[]->" indicates a likely frameshift as judged by lack of conserved residues and by sequence length.

```
   19 []->
    6 HD []->
    2 HD #
    2 HD HD []->
    4 HD HD HD HD HD []->
    1 HD HD HD HD HD NG HD []->
   23 HD HD HD HD HD NG HD NN HD ND NG NG NN HG ND ND HD NG
   12 HD HD HD HD HD NG HD NN HD NG HG NN HD N* NG NG
   12 HD HD NC HD NC NG HD HH HD NI NG N* NS N* HD HD NS NI HG HD NG
    1 HD HD NG NI HG HD HG []->
    1 HD HD NG NI HG HD HG NI HD []->
    1 HD HD NG NI HG HD HG NI HD NN HD HG HG HG []->
   10 HD HD NG NI HG HD HG NI HD NN HD HG HG HG NG NI NG
    8 HD HD NN NN NG N* HD NI NG HD NG NS HD HA ND N* ND NN HD NN NN HD HD N*
NN NG HD
    4 HD HG HD []->
   18 HD HG HD HG N* NN NG HD NN HD NG NG NN HD N* NG NG
    1 HD NN HD []->
   32 HD NN HD N* NS NN HG NI NI NS NI NG HD NN HD HD NG HG NG
   12 HH NN HD HD NG N* HD NN HD ND N* NG NS NS NN HD HD NG
    4 NI []->
```

```
     1 NI HG []->
    12 NI HG NI N* HN NG NN ND HD NG HD NI HG HG HD NN HD HH N* HD NN NN NG HD
NN NN NG NN NI NG
    12 NI HG N* NN N* HD HG HD NN HD NK HG NN NN NG HD
    11 NI NG NI NG NN NG NN NG HD NN NN HG HD NN NS NN HD HD NG NA NN HD HD HD
HD N*
     2 NI NN []->
    24 NI NN HN NN NI! NG HD NN HD HG HD HG HG HD HD NG
    24 NI N* NI NI NN N* NI NK HD HD HD NG NI NN HD NN HD NG
     2 NI NN NI []->
    12 NI NN NI NI NG HD NG NN NI NN NI NN HD
    13 NI NN NI NI NI NI N* NI NG NN NN NN NI NS NG NG
     5 NN []->
    20 NN HD HD HH NN NG NS NG HD NI NG HD HH NG NG NG HA NG
    20 NN HD NI HD NN NG NG HD NN HD N* NN HD HD HD NG
     6 NN HD NI NI []->
    16 NN HD NI NI NG NN NN HD NN NI NI NN NN NN NG NI HD NG
    10 NN HD NI NI NN HA NN NS NS NI HD HA HA HA HD HD HD HA HD N*
     4 NN HD NI NK HD []->
     2 NN HD NI NK HD HD []->
     4 NN HD NI NK HD HD HD NG NI []->
     2 NN HD NI NK HD HD HD NG NI NN HD!
     2 NN HD NI NK HD HD HD NG NI NN HD HD!
    26 NN HD NI NK HD HD HD NG NI NN HD HD NG
    18 NN HD NS NG HD NN HD NI HD NN HD NN HD NN HD NN NN NN NN NN NN NN HD NG
    22 NN NG HD NG HD HD NI NH HG NI NN NN HD NS NG NN HD NG NG N*
     1 NN NN ND N* NS NS []->
    14 NN NN ND N* NS NS YG NI SN HD HD NG HD NS NN HD NG
    16 NN NS NG NI HD HD NG NN HD NI NG N* N* NG HD HD NS NI NN NG NG
    26 NS HD NI N* NI HG NI NI N* NG HD NN NI NG HD NN NS NN NG NN
    14 NS HG NI NG NS NN ND N* NG N* HN NN HD NS NI NN HD HD NG NG HG HD NG
    19 NS HG NI NG NS NN NG NN NG HD NN HD HD NG
     1 NS ND HG HG []->
     1 NS ND HG HG HG []->
    22 NS ND HG HG HG NG! HG HG HD HD HD NN NN HD HG HH H* H* NN HD H*
```
