# Supplementary material for: Single molecule real-time sequencing of Xanthomonas oryzae genomes reveals a dynamic structure and complex TAL (transcription activator-like) effector gene relationships
Source: Microb Genom. 2015 Oct 30;1(4):e000032. doi: 10.1099/mgen.0.000032 (PMC4853030; doi:10.1099/mgen.0.000032)

Supplementary Material
for

SMRT SEQUENCING OF *XANTHOMONAS ORYZAE* GENOMES REVEALS A DYNAMIC STRUCTURE AND COMPLEX TAL EFFECTOR GENE RELATIONSHIPS

Nicholas J. Booher[1], Sara C. D. Carpenter[1], Robert P. Sebra[2], Li Wang[1], Steven L. Salzberg[3], Jan E. Leach[4], and Adam J. Bogdanove[1]*
Address: [1] Plant Pathology and Plant-Microbe Biology Section, School of Integrative Plant Science, Cornell University, Ithaca, NY 14853 USA; [2] Icahn Institute for Genomics and Multiscale Biology and Department of Genetics & Genomic Sciences, Icahn School of Medicine at Mount Sinai, New York, NY 10029 USA; [3] Departments of Biomedical Engineering, Computer Science, and Biostatistics and Center for Computational Biology, Johns Hopkins University, Baltimore, MD 21205 USA; [4] Bioagricultural Sciences and Pest Management, Colorado State University, Ft. Collins, CO 80523 USA
***Corresponding author: ajb7@cornell.edu**

File S3. Southern blot confirming the internal deletion in *tal7b* or *tal8b* of PXO99A. The blot was prepared using *Sph*I-digested genomic DNA of the sequenced PXO99A accession (PXO99A-s) and another accession of PXO99A that lacks the 212 kb duplication (PXO99A-B; see text). The blot was probed with the *Sph*I fragment of *avrXa7* using the AlkPhos Direct Labeling and Detection System with CDP-Star (GE Healthcare Life Sciences, Pittsburgh, PA) according to manufacturer's directions. Arrows mark the *Sph*I fragment of the full length *tal7b* or *tal8b* gene and that of the gene with the internal deletion. Note that the *Sph*I fragment of the full-length gene co-migrates with those of *tal6a* and *tal9a*, which have the same number of repeats.

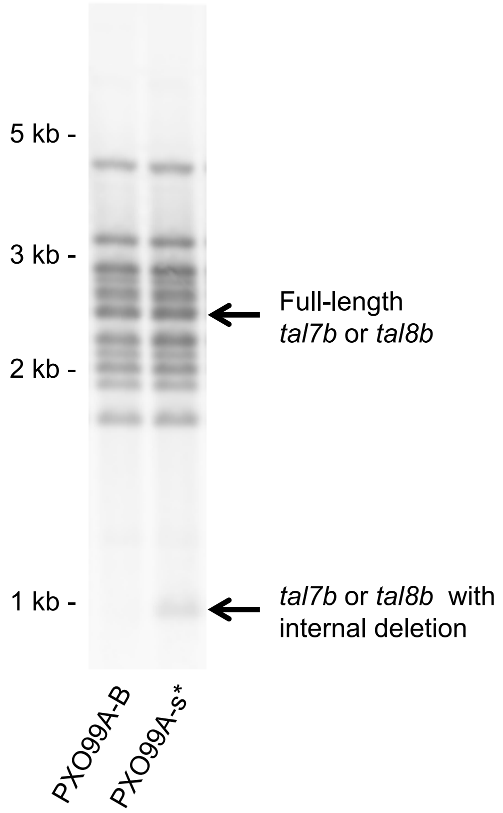

Supplement: Supplementary file 3 — Supplementary Data [file mgen-01-32-s003.pdf]
