# Supplementary material for: Single molecule real-time sequencing of Xanthomonas oryzae genomes reveals a dynamic structure and complex TAL (transcription activator-like) effector gene relationships
Source: Microb Genom. 2015 Oct 30;1(4):e000032. doi: 10.1099/mgen.0.000032 (PMC4853030; doi:10.1099/mgen.0.000032)
Supplement: Supplementary file 4 — Supplementary Data [file mgen-01-32-s004.pdf]

Supplementary Material
for

SMRT SEQUENCING OF *XANTHOMONAS ORYZAE* GENOMES REVEALS A DYNAMIC STRUCTURE AND COMPLEX TAL EFFECTOR GENE RELATIONSHIPS

Nicholas J. Booher[1], Sara C. D. Carpenter[1], Robert P. Sebra[2], Li Wang[1], Steven L. Salzberg[3], Jan E. Leach[4], and Adam J. Bogdanove[1]*
Address: [1] Plant Pathology and Plant-Microbe Biology Section, School of Integrative Plant Science, Cornell University, Ithaca, NY 14853 USA; [2] Icahn Institute for Genomics and Multiscale Biology and Department of Genetics & Genomic Sciences, Icahn School of Medicine at Mount Sinai, New York, NY 10029 USA; [3] Departments of Biomedical Engineering, Computer Science, and Biostatistics and Center for Computational Biology, Johns Hopkins University, Baltimore, MD 21205 USA; [4] Bioagricultural Sciences and Pest Management, Colorado State University, Ft. Collins, CO 80523 USA
***Corresponding author: ajb7@cornell.edu**

File S4. Genomic regions respectively unique to BLS256 or CFBP7342. Regions unique to each genome were determined by aligning the other genomes to it with Nucmer 3.1 (Kurtz *et al.*, 2004), using delta-filter (Kurtz *et al.*, 2004) to filter to a many-to-many alignment allowing rearrangements, getting the complement of the aligned regions using bedtools 2.17 (Quinlan & Hall, 2010), and filtering the results to those 100 bp or longer. The coordinates and length of each unique region are given, followed by the product name, coordinates, and strand of any annotated protein coding sequences overlapping the region.

Regions unique to BLS256 when compared to CFBP7342:

```
Region 0, 19236-19739 (503):

site-specific DNA methylase   19475     19751     +

Region 1, 27341-31250 (3909):

regulatory protein cII, putative   27404     28757     -
sensor kinase  29460     31011     -

Region 2, 35825-35932 (107):
Region 3, 36080-38975 (2895):

Hypothetical protein36319     37402     +
hypothetical protein38128     38275     -
```

conserved hypothetical protein38524 38704 +

Region 4, 40045-43479 (3434):

membrane-fusion protein, putative 40060 41329 +
colicin V secretion ABC transporter ATP-binding protein41325 43428 +

Region 5, 90402-90518 (116):
Region 6, 93234-93957 (723):

hypothetical protein93723 94419 -

Region 7, 110167-113330 (3163):

exodeoxyribonuclease V, gamma subunit 106769 110183 -
hemagglutinin 110535 111495 -
microcystin dependent protein 111517 111658 +
microcystin dependent protein 111654 111816 +
acetyltransferase 111820 112378 +
conserved hypothetical protein112396 112684 -
hypothetical protein112682 112865 +
ABC transporter ATP-binding protein113068 113863 +

Region 8, 125677-129127 (3450):

EF hand domain protein 126499 129109 -

Region 9, 175198-181065 (5867):

conserved hypothetical protein175377 176022 -
LasA 176193 177012 -
conserved hypothetical protein177183 177657 +
proline-betaine transporter 178015 179263 -
thioredoxin 179967 180240 -
AtsE 180260 180659 -
esterase 180728 180959 -
conserved hypothetical protein180955 181204 -

Region 10, 224827-225444 (617):

conserved hypothetical protein224951 225230 +

Region 11, 226965-227462 (497):
Region 12, 228534-228683 (149):
Region 13, 234362-234666 (304):

ISXo8 transposase, IS4 family 232966 234394 -

Region 14, 236435-236710 (275):
Region 15, 255746-255967 (221):
Region 16, 302192-302312 (120):
Region 17, 419848-420833 (985):

5'-nucleotidase419844   420804   -

Region 18, 513958-516758 (2800):

conserved hypothetical protein513965   516209   -

Region 19, 631103-631324 (221):

prolyl oligopeptidase family protein   630646   632083   +

Region 20, 718116-718660 (544):

Rhs element Vgr protein 717511   718720   +

Region 21, 1050490-1053031 (2541):

plasmid mobilization protein 1050487   1050730   -
MchC protein   1051341   1053039   +

Region 22, 1187384-1189609 (2225):

putative transcriptional regulator, TetR family   1187289
1188048   +
conserved hypothetical protein1188377   1188908   +
conserved hypothetical protein1188918   1189245   +
conserved hypothetical protein1189256   1189943   +

Region 23, 1190530-1191066 (536):

type I restriction enzyme StySJI specificity protein   1190290
1191655   -

Region 24, 1191175-1191629 (454):

type I restriction enzyme StySJI specificity protein   1190290
1191655   -

Region 25, 1204321-1204860 (539):

type I restriction-modification system, S subunit 1204180
1206484   +

Region 26, 1301199-1302901 (1702):

conserved hypothetical protein1301342 1302755 +
Rhs element Vgr protein 1302899 1305188 +

Region 27, 1316418-1318032 (1614):

EF hand domain protein 1317269 1320206 -

Region 28, 1353875-1358703 (4828):

Rhs element Vgr protein 1351213 1353973 +
conserved hypothetical protein1354243 1354969 +
hypothetical protein1354959 1355538 -
conserved hypothetical protein1354980 1357965 +

Region 29, 1359055-1361861 (2806):
Region 30, 1371454-1371670 (216):

hypothetical protein1371242 1371686 +

Region 31, 1379092-1379265 (173):
Region 32, 1509735-1512031 (2296):

conserved hypothetical protein1509363 1510104 +
conserved hypothetical protein1510498 1511047 +

Region 33, 1517485-1517886 (401):
Region 34, 1914113-1915206 (1093):

conserved hypothetical protein1912669 1915045 +

Region 35, 1939489-1939767 (278):

phospholipase A1, putative 1939187 1939793 -

Region 36, 1946125-1946258 (133):
Region 37, 2198819-2200005 (1186):

conserved hypothetical protein2199311 2199752 -

Region 38, 2201219-2201369 (150):

tannase 2201182 2202328 +

Region 39, 2230670-2231074 (404):

conserved hypothetical protein2228831 2230691 +

Region 40, 2281927-2282613 (686):

chemotaxis protein 2282401   2284663   -

Region 41, 2283724-2285256 (1532):

chemotaxis protein 2282401   2284663   -
methyl-accepting chemotaxis protein2285075   2287337   -

Region 42, 2302132-2304906 (2774):

chemotaxis protein 2301250   2303608   -
conserved hypothetical protein2303636   2304386   -
methyl-accepting chemotaxis protein2304713   2307494   -

Region 43, 2308101-2308593 (492):
Region 44, 2331042-2332403 (1361):
Region 45, 2332589-2333734 (1145):

conserved hypothetical protein2332554   2332896   +
conserved hypothetical protein2332988   2333309   +

Region 46, 2335088-2340818 (5730):

type III restriction-modification system methyltransferase
2335132   2337028   +
type III restriction enzyme   2337747   2340810   +

Region 47, 2342167-2343383 (1216):

conserved hypothetical protein2342194   2342860   -

Region 48, 2526749-2527849 (1100):

conserved hypothetical protein2527052   2527250   +
conserved hypothetical protein2527277   2528006   +

Region 49, 2532132-2532825 (693):
Region 50, 2533421-2534568 (1147):

2-oxoglutarate-dependent ethylene-succinate-forming enzyme
2533499   2534501   +

Region 51, 2536433-2537620 (1187):

conserved hypothetical protein2536480   2537167   -

Region 52, 2578616-2579266 (650):
Region 53, 2580348-2581031 (683):

ClpB 2580933 2583639 -

Region 54, 2593941-2595113 (1172):

conserved hypothetical protein2593867 2594713 +
conserved hypothetical protein2594752 2597599 +

Region 55, 2595429-2595770 (341):

conserved hypothetical protein2594752 2597599 +

Region 56, 2597305-2597945 (640):

conserved hypothetical protein2594752 2597599 +
conserved hypothetical protein2597704 2598631 +

Region 57, 2598670-2598888 (218):

conserved hypothetical protein2598703 2600710 +

Region 58, 2600415-2601089 (674):

conserved hypothetical protein2598703 2600710 +
conserved hypothetical protein2600718 2601750 +

Region 59, 2601789-2602009 (220):

conserved hypothetical protein2601822 2603838 +

Region 60, 2603566-2604166 (600):

conserved hypothetical protein2601822 2603838 +
conserved hypothetical protein2603952 2604864 +

Region 61, 2604903-2605121 (218):

conserved hypothetical protein2604936 2605404 +

Region 62, 2684401-2686860 (2459):

RND superfamily protein 2684411 2687480 -

Region 63, 2694733-2695641 (908):

integrase 2694811 2695657 +

Region 64, 2696703-2697889 (1186):
Region 65, 2705964-2713474 (7510):

colicin V secretion-processing ATP-binding protein CvaB2705981 2708171 -
putative secretion protein 2708187 2709483 -
membrane translocator, putative 2710291 2712937 -

Region 66, 2926060-2926356 (296):

hypothetical protein2926258 2926372 +

Region 67, 2927524-2928887 (1363):

hypothetical protein2928074 2928203 -
conserved hypothetical protein2928456 2929254 -

Region 68, 3041210-3041916 (706):

integral membrane protein3041343 3042072 -

Region 69, 3145120-3147278 (2158):

transposase 3145817 3146810 -

Region 70, 3325471-3325586 (115):

TonB-dependent receptor 3324490 3327202 +

Region 71, 3341588-3341999 (411):

hypothetical protein3341985 3342105 +

Region 72, 3342432-3344131 (1699):
Region 73, 3366273-3366776 (503):

site-specific DNA methylase 3366512 3366788 +

Region 74, 3370968-3371406 (438):

hypothetical protein3370261 3371452 +

Region 75, 3545233-3546569 (1336):

RhsD protein 3545175 3550020 -

Region 76, 3599119-3600297 (1178):

chemotaxis protein	3597892	3599647	+
conserved protein YuxK	3599798	3600233	-
conserved hypothetical protein3600219	3600609	-

Region 77, 3627016-3627927 (911):

RhsD protein	3624579	3627546	+

Region 78, 3652475-3653587 (1112):

site-specific recombinase, phage integrase family 3652405
3652987	+
phage-related protein	3652990	3653413	+

Region 79, 3665565-3668721 (3156):

carboxylesterase	3665536	3666361	-
tRNA-rRNA methyltransferase	3666625	3667687	-
hypothetical protein3667797	3667920	-
acetyltransferase	3667919	3668057	-
acetyltransferase	3668053	3668191	-
hypothetical protein3668146	3668311	+
hypothetical protein3668502	3668724	+

Region 80, 3784299-3784736 (437):

transcriptional regulator3783294	3785775	+

Region 81, 3917554-3918197 (643):

general secretion pathway protein D3917421	3919710	-

Region 82, 3996762-3997154 (392):
Region 83, 4034809-4035353 (544):

conserved hypothetical protein4034652	4035801	-

Region 84, 4039641-4048989 (9348):

ABC transporter permease 4038833	4040195	+
ABC transporter ATP-binding protein4040243	4040963	+
ABC transporter permease 4040974	4042276	+
ABC transporter permease 4042332	4043559	+
ABC transporter permease 4043601	4044930	+
ABC transporter permease 4044929	4046165	+
two-component system response regulator protein	4046274
4047618	+

two-component system sensor protein4047614   4048973   +

Region 85, 4132078-4133735 (1657):

methyltransferase   4131747   4133211   -
two-component system response regulator protein   4133355
4133736   +
conserved hypothetical protein4133729   4133972   -

Region 86, 4188613-4189278 (665):
Region 87, 4190442-4191906 (1464):

filamentous hemagglutinin4190875   4196920   -

Region 88, 4192803-4193730 (927):

filamentous hemagglutinin4190875   4196920   -

Region 89, 4197518-4199651 (2133):

conserved hypothetical protein4197642   4197906   +
hypothetical protein4197890   4198019   +
hypothetical protein4198324   4198441   -
filmentous hemagglutinin 4198459   4202431   -

Region 90, 4202533-4204552 (2019):

hypothetical protein4202401   4202539   +
filamentous hemagglutinin4203355   4211800   -

Region 91, 4212012-4214127 (2115):

conserved hypothetical protein4212138   4212417   +
hypothetical protein4212535   4212664   -
filamentous hemagglutinin4212941   4224182   -

Region 92, 4228407-4229090 (683):
Region 93, 4232262-4232421 (159):
Region 94, 4239270-4244001 (4731):

hypothetical protein4239264   4239378   -
hypothetical protein4239475   4239733   -
ABC transporter permease 4239752   4241000   -
ABC transporter permease 4240996   4242304   -
hypothetical protein4242316   4242907   -
conserved hypothetical protein4243085   4243493   -

Region 95, 4248891-4249637 (746):

conserved hypothetical protein4248884   4249673   -
permease 4249636   4250428   -

Region 96, 4328685-4329808 (1123):

conserved hypothetical protein4328409   4329603   +

Region 97, 4387730-4388552 (822):

acyltransferase4386863   4387991   -
integrase, catalytic region   4388001   4388595   -

Region 98, 4418747-4419060 (313):
Region 99, 4461780-4462135 (355):

tRNA-dihydrouridine synthase A4460826   4461831   -

Region 100, 4515601-4515865 (264):
Region 101, 4517350-4517653 (303):
Region 102, 4532459-4532854 (395):
Region 103, 4632012-4632372 (360):

hypothetical protein4632295   4632412   -

Region 104, 4652892-4654473 (1581):

phage integrase4653074   4653401   -
phage integrase4653553   4654312   -

Region 105, 4802079-4803058 (979):

restriction endonuclease 4802305   4803097   -

Regions unique to CFBP7342 when compared to BLS256:

Region 0, 91373-91537 (164):
Region 1, 196564-197408 (844):
Region 2, 216202-216550 (348):
Region 3, 221542-221668 (126):

hypothetical protein216558   225117   -

Region 4, 312662-313965 (1303):

methylamine utilization protein   312245   313292   -

Region 5, 314472-323524 (9052):

hypothetical protein314554   317770   +

Region 6, 326675-327261 (586):

hypothetical protein326849   327083   +

Region 7, 407086-409237 (2151):

hypothetical protein407999   409028   -
tRNA-dihydrouridine synthase A409179   410190   +

Region 8, 467258-467393 (135):
Region 9, 468555-468687 (132):
Region 10, 488073-499778 (11705):

hypothetical protein488049   488292   -
hypothetical protein488509   489028   -
hypothetical protein489042   489882   -
hypothetical protein490112   491135   -
hypothetical protein491539   491758   -
hypothetical protein491859   492210   -
tail fiber protein 492213   492666   -
hypothetical protein492731   493067   -
hypothetical protein493063   493465   -
head-tail adaptor protein493457   493790   -
hypothetical protein493786   494203   -
hypothetical protein494206   494431   -
capsid protein 494490   495735   -
peptidase S14 495800   496529   -
nucleoid-structuring protein H-NS 496494   497811   -
terminase 497810   499487   -
hypothetical protein499489   499870   -

Region 11, 500371-500871 (500):

holin   499970   500381   -
hypothetical protein500403   500679   -
hypothetical protein500675   501092   -

Region 12, 608444-608926 (482):
Region 13, 628987-632682 (3695):

hypothetical protein630208   630439   -
hypothetical protein630642   630831   -
hypothetical protein630897   631275   -
peptidase S24 631539   632232   -

Region 14, 632813-634786 (1973):

hypothetical protein632766   633012   +
hypothetical protein633008   633290   +
hypothetical protein633856   634156   +
DNA helicase   634152   635508   +

Region 15, 636672-637238 (566):

hypothetical protein636768   637236   +

Region 16, 641632-642756 (1124):

hypothetical protein639284   642122   +
hypothetical protein642146   642881   +

Region 17, 644761-646896 (2135):

hypothetical protein642907   645250   +
hypothetical protein645270   646008   +
hypothetical protein646172   647102   +

Region 18, 652574-653790 (1216):

hypothetical protein650733   653076   +
hypothetical protein653103   653808   +

Region 19, 654992-655241 (249):
Region 20, 678357-678937 (580):

hypothetical protein678358   678943   +

Region 21, 684963-686522 (1559):

hypothetical protein685069   685411   +
hypothetical protein685561   685858   +
hypothetical protein685899   686211   +
hypothetical protein686338   686554   +

Region 22, 707580-708822 (1242):
Region 23, 711200-712076 (876):

hypothetical protein711645   711999   +

Region 24, 850869-851401 (532):

hypothetical protein850420   851557   +

Region 25, 853208-854047 (839):

copper resistance protein CopB852742 853819 -
copper resistance protein CopA853815 855630 -

Region 26, 965232-965890 (658):

general secretion pathway protein GspD 963712 966022 +

Region 27, 983397-983525 (128):
Region 28, 1003723-1004082 (359):

hypothetical protein1003679 1003895 +

Region 29, 1017454-1018168 (714):
Region 30, 1020451-1021345 (894):
Region 31, 1100631-1103277 (2646):

type IV secretion protein Rhs 1097187 1101927 +
hypothetical protein1101907 1102318 +
hypothetical protein1102767 1103226 +

Region 32, 1109448-1111150 (1702):
Region 33, 1164137-1165231 (1094):
Region 34, 1166799-1167268 (469):

hypothetical protein1166847 1167417 +

Region 35, 1229902-1268972 (39070):

hypothetical protein1230332 1231022 +
hypothetical protein1231123 1231720 -
hypothetical protein1231716 1232658 -
peptidase S24 1232889 1233369 +
PAAR motif family protein1235467 1235869 +
hypothetical protein1235817 1236375 +
hypothetical protein1236355 1236697 +
adenine methyltransferase1238258 1238969 -
hypothetical protein1238967 1239156 +
Presumed portal vertex protein1239152 1240172 -
terminase 1240171 1241935 -
phage capsid scaffolding protein 1242077 1242920 +
capsid protein 1242966 1243983 +
terminase endonuclease subunit1243986 1244706 +
head completion/stabilization protein 1244805 1245273 +
tail protein 1245272 1245482 +
membrane protein 1245486 1245843 +
hypothetical protein1245835 1246111 +

lysozyme 1246110   1246749   +
hypothetical protein1246748   1247237   +
hypothetical protein1247640   1248090   +
baseplate assembly protein   1248171   1249062   +
tail protein   1249054   1249600   +
hypothetical protein1249609   1251115   +
tail fiber assembly protein   1251122   1251701   +
baseplate assembly protein   1251761   1252325   +
baseplate assembly protein   1252321   1252681   +
tail sheath protein 1252692   1253859   +
major tail tube protein 1253889   1254399   +
tail protein   1254443   1254746   +
P2 GpE family protein   1254754   1254868   +
tail protein   1254900   1257771   +
oxidoreductase 1257783   1258185   +
phage late control protein   1258181   1259171   +
hypothetical protein1260563   1261001   -
hypothetical protein1261072   1261330   +
hypothetical protein1261452   1261653   +
hypothetical protein1261663   1261942   +
hypothetical protein1261938   1262151   +
hypothetical protein1262159   1264856   +
hypothetical protein1265167   1265386   +
hypothetical protein1265382   1265661   +
hypothetical protein1265886   1266297   +
hypothetical protein1266458   1266734   +
hypothetical protein1266730   1266976   +
hypothetical protein1266972   1267245   +
hypothetical protein1267241   1267448   +
hypothetical protein1267444   1267666   +
integrase 1267665   1268850   +

Region 36, 1320573-1320725 (152):

chemotaxis protein CheY 1316458   1323532   +

Region 37, 1350033-1350436 (403):
Region 38, 1366414-1366568 (154):

TonB-dependent receptor 1364821   1367548   -

Region 39, 1546027-1546414 (387):
Region 40, 1646855-1650784 (3929):

transposase   1646939   1647341   +
transposase   1647358   1647745   +
ATP-dependent OLD family endonuclease   1647931   1649710   +

Region 41, 1652316-1689187 (36871):

hypothetical protein1652373 1652649 -
hypothetical protein1652914 1653139 -
hypothetical protein1653135 1653408 -
hypothetical protein1653569 1653977 -
hypothetical protein1654055 1654322 -
hypothetical protein1654318 1654597 -
hypothetical protein1654593 1654812 -
hypothetical protein1655135 1657832 -
hypothetical protein1657841 1658054 -
hypothetical protein1658050 1658329 -
hypothetical protein1658339 1658660 -
hypothetical protein1658991 1659429 +
phage late control protein 1660088 1661075 -
oxidoreductase 1661071 1661473 -
tail protein 1661485 1664356 -
P2 GpE family protein 1664388 1664502 -
tail protein 1664510 1664813 -
major tail tube protein 1664858 1665368 -
tail sheath protein 1665398 1666565 -
baseplate assembly protein 1666576 1666936 -
baseplate assembly protein 1666932 1667496 -
tail fiber assembly protein 1667556 1668135 -
hypothetical protein1668142 1669648 -
tail protein 1669657 1670203 -
baseplate assembly protein 1670195 1671086 -
hypothetical protein1672379 1672832 -
tail protein 1672819 1673239 -
hypothetical protein1673235 1673724 -
lysozyme 1673723 1674362 -
hypothetical protein1674361 1674637 -
membrane protein 1674629 1674986 -
tail protein 1674990 1675200 -
head completion/stabilization protein 1675199 1675667 -
terminase endonuclease subunit1675766 1676486 -
capsid protein 1676489 1677509 -
phage capsid scaffolding protein 1677555 1678398 -
terminase 1678540 1680304 +
Presumed portal vertex protein1680303 1681323 +
hypothetical protein1681319 1681508 -
adenine methyltransferase1681506 1682217 +
hypothetical protein1682325 1682562 +
hypothetical protein1683747 1684089 -
hypothetical protein1684069 1684627 -
PAAR motif family protein1684575 1684977 -
hypothetical protein1685788 1686019 -
hypothetical protein1686018 1686318 -

hypothetical protein1686563   1687085   -
integrase 1687430   1689221   -
ribosomal protein S12 methylthiotransferase 1689150   1690512
+

Region 42, 1921536-1921725 (189):

hypothetical protein1921516   1921756   -

Region 43, 1924760-1925852 (1092):

hypothetical protein1924602   1925334   -
hypothetical protein1925362   1928197   -

Region 44, 1928400-1929235 (835):

hypothetical protein1928193   1929123   -
type IV secretion protein Rhs 1929131   1931894   -

Region 45, 1929797-1929927 (130):

type IV secretion protein Rhs 1929131   1931894   -

Region 46, 1932002-1932282 (280):
Region 47, 2064015-2066491 (2476):

DNA-binding protein 2064108   2064303   -
DNA-binding protein 2064549   2065485   -
hypothetical protein2065706   2065955   +
hypothetical protein2066072   2066399   +

Region 48, 2067694-2068122 (428):

hypothetical protein2067673   2067946   -

Region 49, 2219836-2221901 (2065):
Region 50, 2222994-2227969 (4975):
Region 51, 2229404-2229679 (275):

transposase   2229643   2229916   +

Region 52, 2326340-2327375 (1035):
Region 53, 2332317-2332470 (153):

hypothetical protein2331845   2333033   +

Region 54, 2526370-2547037 (20667):

hypothetical protein2527369   2527642   -
replication protein 2527702   2528773   +
hypothetical protein2528881   2529172   +
hypothetical protein2529181   2529382   +
hypothetical protein2529410   2529635   +
hypothetical protein2531153   2531444   +
zonular occludens toxin 2531443   2532586   +
hypothetical protein2532674   2532968   +
hypothetical protein2534135   2534408   -
replication protein 2534468   2535539   +
hypothetical protein2535647   2535938   +
hypothetical protein2535947   2536148   +
hypothetical protein2536176   2536401   +
hypothetical protein2537919   2538210   +
zonular occludens toxin 2538209   2539352   +
hypothetical protein2539440   2539734   +
hypothetical protein2539902   2540286   -
replication protein 2541197   2542268   +
hypothetical protein2542376   2542667   +
hypothetical protein2542676   2542877   +
hypothetical protein2542905   2543130   +
hypothetical protein2544648   2544939   +
zonular occludens toxin 2544938   2546081   +
hypothetical protein2546169   2546445   +

Region 55, 2583625-2583785 (160):
Region 56, 2587875-2589386 (1511):

hypothetical protein2587839   2588046   -
hypothetical protein2588394   2589225   -
ATPase AAA2589288   2592012   -

Region 57, 2601444-2601574 (130):

type IV secretion protein Rhs 2599476   2602239   +

Region 58, 2602314-2604988 (2674):

hypothetical protein2602180   2603086   +
hypothetical protein2603105   2605973   +

Region 59, 2605679-2606332 (653):

hypothetical protein2603105   2605973   +
hypothetical protein2605984   2607010   +

Region 60, 2607016-2607319 (303):
Region 61, 2608032-2608684 (652):

hypothetical protein2608317   2609340   +

Region 62, 2610901-2611521 (620):

hypothetical protein2611199   2612219   +

Region 63, 2706334-2706454 (120):
Region 64, 2713347-2713468 (121):
Region 65, 2820275-2820526 (251):

hypothetical protein2820223   2820496   -

Region 66, 2904495-2909688 (5193):

chemotaxis protein CheY 2906615   2906960   +
histidine kinase   2907067   2909923   -

Region 67, 2955697-2995119 (39422):

hypothetical protein2955956   2956208   -
hypothetical protein2956352   2957081   -
hypothetical protein2957080   2957638   -
hypothetical protein2957706   2958921   -
hypothetical protein2959961   2962052   -
hypothetical protein2967612   2967924   -
hypothetical protein2967933   2968146   -
hypothetical protein2968157   2969366   -
hypothetical protein2969381   2970287   -
terminase 2972357   2973683   -
hypothetical protein2974461   2974944   +
hypothetical protein2974971   2975187   -
hypothetical protein2975183   2975510   -
hypothetical protein2975509   2975992   -
hypothetical protein2976183   2976633   -
hypothetical protein2976859   2977333   -
hypothetical protein2977329   2977569   -
hypothetical protein2977565   2977952   -
hypothetical protein2977944   2978145   -
hypothetical protein2978434   2978755   -
hypothetical protein2978751   2979195   -
hypothetical protein2979194   2979380   -
hypothetical protein2980440   2980779   -
hypothetical protein2981080   2981272   -
phosphoadenosine phosphosulfate reductase   2981442   2982390   -
hypothetical protein2982389   2982686   -
hypothetical protein2983803   2984091   -

hypothetical protein2984308   2984494   -
hypothetical protein2984699   2985215   +
hypothetical protein2985222   2985429   -
repressor 2985500   2986178   +
hypothetical protein2986221   2986512   +
hypothetical protein2987003   2987486   -
hypothetical protein2987497   2987698   -
hypothetical protein2987862   2988198   +
hypothetical protein2988284   2988650   +
hypothetical protein2989187   2989403   +
hypothetical protein2989399   2989609   +
hypothetical protein2989605   2989815   +
hypothetical protein2990277   2990475   +
hypothetical protein2990512   2991472   +
DNA recombinase2991480   2992395   +
recombinase   2992391   2993159   +
methyltransferase   2993298   2993814   +
hypothetical protein2993800   2994121   +
hypothetical protein2994195   2994753   -

Region 68, 3209229-3209355 (126):

transposase   3209197   3210148   +

Region 69, 3210155-3210300 (145):

integrase 3210294   3210552   -

Region 70, 3288944-3291362 (2418):

sulfatase modifying factor 1 3289085   3290048   +
hypothetical protein3290100   3291339   -

Region 71, 3292569-3295343 (2774):

hypothetical protein3292528   3293593   -
sulfatase modifying factor 1 3293651   3294611   +
hypothetical protein3294663   3295281   -

Region 72, 3296860-3302853 (5993):

sulfatase modifying factor 1 3298526   3299492   +
DNA repair protein 3299544   3302610   -
type IV secretion protein Rhs 3302596   3305536   -

Region 73, 3306051-3313998 (7947):

sulfatase modifying factor 1 3306192   3307149   +

sulfatase modifying factor 1 3309540   3310500   +
sulfatase modifying factor 1 3312991   3313951   +

Region 74, 3315312-3316063 (751):

hypothetical protein3315347   3316028   -

Region 75, 3317228-3319815 (2587):
Region 76, 3323762-3324154 (392):
Region 77, 3328346-3328598 (252):

hypothetical protein3328558   3328846   -

Region 78, 3459440-3459552 (112):
Region 79, 3484876-3486407 (1531):

calcium-binding protein 3482701   3485596   +

Region 80, 3491608-3491762 (154):

hypothetical protein3490786   3492220   +

Region 81, 3504568-3504722 (154):

hypothetical protein3503746   3505180   +

Region 82, 3519524-3520952 (1428):

hypothetical protein3519993   3520839   -
type VI secretion protein3520865   3521249   -

Region 83, 3528382-3532849 (4467):

hypothetical protein3528460   3529783   -
hypothetical protein3530283   3530763   -
hypothetical protein3530912   3531392   -
hypothetical protein3531415   3531880   -
hypothetical protein3531876   3532773   -

Region 84, 3534135-3536743 (2608):

hypothetical protein3534200   3535772   -

Region 85, 3537921-3538765 (844):

type IV secretion protein Rhs 3538668   3541485   -

Region 86, 3627387-3627893 (506):

type I restriction-modification system subunit S 3625762 3628033 -

Region 87, 3640585-3641027 (442):

hypothetical protein3640570 3641956 +

Region 88, 3641136-3641717 (581):

hypothetical protein3640570 3641956 +

Region 89, 3643514-3644251 (737):

integrase 3643821 3644508 +

Region 90, 3646099-3646849 (750):

hypothetical protein3646450 3646780 -

Region 91, 3717782-3718013 (231):
Region 92, 3834022-3834469 (447):

hypothetical protein3834171 3834756 -

Region 93, 4024509-4025303 (794):
Region 94, 4090930-4091419 (489):

xylanase 4090938 4092159 -

Region 95, 4183522-4183653 (131):
Region 96, 4350461-4354728 (4267):

hypothetical protein4350591 4351245 +
transposase 4351241 4353110 +
ATPase AAA4353096 4354002 +

Region 97, 4355932-4360365 (4433):

DNA methyltransferase 4356243 4357089 -
hypothetical protein4358563 4360288 -
lipoyl synthase4360326 4361373 -

Region 98, 4367799-4368197 (398):

hypothetical protein4367945 4368224 -

Region 99, 4369477-4369690 (213):

hypothetical protein4369443   4369626   -
hypothetical protein4369580   4370012   -

Region 100, 4370011-4371747 (1736):

hypothetical protein4369580   4370012   -

Region 101, 4374316-4381838 (7522):

hypothetical protein4374295   4374691   -
hypothetical protein4376126   4377212   -
hypothetical protein4378725   4379265   -
hypothetical protein4379801   4381346   -
hypothetical protein4381345   4382332   -

Region 102, 4685839-4686257 (418):
Region 103, 4688319-4688563 (244):
Region 104, 4739224-4739407 (183):
Region 105, 4807020-4807136 (116):
Region 106, 4808668-4809229 (561):
Region 107, 4812025-4812860 (835):

type IV secretion protein Rhs 4809365   4812128   +
hypothetical protein4812136   4813063   +

Region 108, 4815392-4816596 (1204):

hypothetical protein4813059   4815900   +

Region 109, 4818526-4819634 (1108):

hypothetical protein4816681   4819024   +

Region 110, 4821678-4822946 (1268):

hypothetical protein4819809   4822152   +
hypothetical protein4822180   4822927   +

Region 111, 4825635-4827252 (1617):

hypothetical protein4825466   4825934   -

Region 112, 4834417-4837541 (3124):
Region 113, 4838584-4838711 (127):
Region 114, 4839978-4840728 (750):

membrane protein   4838793   4840536   -

```
Region 115, 4911908-4912033 (125):
Region 116, 4947384-4949870 (2486):

endonuclease   4947411   4948284   -

Region 117, 4961836-4961936 (100):
Region 118, 4977437-4980513 (3076):

membrane protein    4977386   4978511   +
membrane protein    4978523   4979834   +
ABC transporter ATP-binding protein4980251   4981046   +

Region 119, 5018134-5018279 (145):

exodeoxyribonuclease V subunit gamma    5018262   5021676   +

Region 120, 5036348-5036795 (447):

DNA-binding protein 5036520   5036769   -
```

References

Kurtz, S., Phillippy, A., Delcher, A. L., Smoot, M., Shumway, M., Antonescu, C. & Salzberg, S. L. (2004). Versatile and open software for comparing large genomes. *Genome Biol* **5**.

Quinlan, A. R. & Hall, I. M. (2010). BEDTools: a flexible suite of utilities for comparing genomic features. *Bioinformatics* **26**, 841-842.
