# Supplementary material for: Single molecule real-time sequencing of Xanthomonas oryzae genomes reveals a dynamic structure and complex TAL (transcription activator-like) effector gene relationships
Source: Microb Genom. 2015 Oct 30;1(4):e000032. doi: 10.1099/mgen.0.000032 (PMC4853030; doi:10.1099/mgen.0.000032)
Supplement: Supplementary file 5 — Supplementary Data [file mgen-01-32-s005.pdf]

Supplementary Material
for

SMRT SEQUENCING OF *XANTHOMONAS ORYZAE* GENOMES REVEALS A DYNAMIC STRUCTURE AND COMPLEX TAL EFFECTOR GENE RELATIONSHIPS

Nicholas J. Booher[1], Sara C. D. Carpenter[1], Robert P. Sebra[2], Li Wang[1], Steven L. Salzberg[3], Jan E. Leach[4], and Adam J. Bogdanove[1]*
Address: [1] Plant Pathology and Plant-Microbe Biology Section, School of Integrative Plant Science, Cornell University, Ithaca, NY 14853 USA; [2] Icahn Institute for Genomics and Multiscale Biology and Department of Genetics & Genomic Sciences, Icahn School of Medicine at Mount Sinai, New York, NY 10029 USA; [3] Departments of Biomedical Engineering, Computer Science, and Biostatistics and Center for Computational Biology, Johns Hopkins University, Baltimore, MD 21205 USA; [4] Bioagricultural Sciences and Pest Management, Colorado State University, Ft. Collins, CO 80523 USA
***Corresponding author: ajb7@cornell.edu**

File S5. Genomic regions respectively unique in pairwise comparisons of PXO99A-s, PXO86, and MAFF311018. Regions unique to each genome were determined by aligning the other genomes to it with Nucmer 3.1 (Kurtz *et al.*, 2004), using delta-filter (Kurtz *et al.*, 2004) to filter to a many-to-many alignment allowing rearrangements, getting the complement of the aligned regions using bedtools 2.17 (Quinlan & Hall, 2010), and filtering the results to those 100 bp or longer. The coordinates and length of each unique region are given, followed by the product name, coordinates, and strand of any annotated protein coding sequences overlapping the region.

Regions unique to PXO99A-s when compared to PXO86:

```
Region 0, 227316-227451 (135):

conserved hypothetical protein226961    228638    +

Region 1, 267878-268519 (641):
Region 2, 269595-270117 (522):
Region 3, 569037-570900 (1863):
Region 4, 917036-918080 (1044):

LysM domain protein 917231    918506    +

Region 5, 1327712-1328273 (561):
```

lipoprotein, putative   1327675   1328635   -

Region 6, 1352348-1353933 (1585):
Region 7, 1391907-1393466 (1559):

DNA (cytosine-5)-methyltransferase PliMCI   1392095   1393283
     +

Region 8, 1394783-1396947 (2164):

conserved hypothetical protein1394830   1395505   +
transposase TnpA, ISL3 family 1395497   1396916   +
ISXoo2 transposase 1396895   1397951   -

Region 9, 1403480-1404121 (641):

conserved hypothetical protein1403274   1403832   +
carbon-nitrogen hydrolase family protein1403949   1404834   +

Region 10, 1475897-1477482 (1585):
Region 11, 1479681-1480665 (984):

RhsD protein   1477497   1480251   +

Region 12, 1556219-1558079 (1860):

RHS Repeat family   1552766   1557506   +
hypothetical protein1557642   1557897   +
RHS Repeat family   1558032   1559271   +

Region 13, 1567079-1567895 (816):

RhsD protein   1563622   1567909   +

Region 14, 1569058-1569984 (926):
Region 15, 1576004-1576235 (231):
Region 16, 1577861-1578011 (150):

conserved hypothetical protein1577773   1577902   -

Region 17, 1582520-1583619 (1099):

conserved hypothetical protein1582370   1582553   -
conserved hypothetical protein1583129   1585964   -

Region 18, 1854995-1855643 (648):
Region 19, 1877742-1878873 (1131):

conserved hypothetical protein1875771   1878114   +
conserved hypothetical protein1878748   1878877   +

Region 20, 2027648-2027835 (187):
Region 21, 2348349-2350434 (2085):

RhsD protein   2349017   2351069   +

Region 22, 2352438-2352883 (445):

RhsD protein   2351065   2352907   +

Region 23, 2365037-2372949 (7912):

hypothetical protein2365722   2365926   +
phage replication protein2366220   2367135   +
helix-destabilizing protein   2367239   2367539   +
hypothetical protein2367542   2367662   +
hypothetical protein2367902   2368142   +
phage-related protein   2368917   2369733   +
phage-related protein   2370051   2371236   +

Region 24, 2374772-2374972 (200):
Region 25, 2659415-2659623 (208):
Region 26, 2661759-2662733 (974):

Rhs family protein 2662288   2665315   -

Region 27, 2666576-2666923 (347):
Region 28, 2668006-2668325 (319):

hypothetical protein2667949   2668135   -

Region 29, 2696796-2697059 (263):
Region 30, 3053843-3054084 (241):

hypothetical protein3053865   3053979   +

Region 31, 3054306-3054460 (154):
Region 32, 3059151-3059292 (141):
Region 33, 3169064-3169553 (489):

conserved hypothetical protein3166718   3169553   +

Region 34, 3170378-3170999 (621):

conserved hypothetical protein3170806   3171133   +

Region 35, 3178361-3178950 (589):

conserved hypothetical protein3178944   3179652   +

Region 36, 3317329-3317762 (433):

conserved hypothetical protein3317674   3318283   -

Region 37, 3318466-3319060 (594):

conserved hypothetical protein3318308   3318608   -

Region 38, 3320223-3320721 (498):

conserved hypothetical protein3320233   3323068   -

Region 39, 3324289-3325284 (995):
Region 40, 3325496-3327111 (1615):

conserved hypothetical protein3325743   3327123   +

Region 41, 3328101-3337888 (9787):

DNA methylase 3327960   3328671   -
phage portal protein, pbsx family 3328859   3329885   -
phage terminase, ATPase subunit   3329881   3331483   -
phage capsid scaffolding protein (GPO) 3331787   3332630   +
phage major capsid protein, P2 family   3332676   3333693   +
phage-related terminase 3333696   3334416   +
phage head completion protein (GPL)3334515   3334983   +
phage Tail Protein X3334997   3335192   +
phage-related protein   3335196   3335553   +
phage-related protein   3335530   3335821   +
phage-related lytic enzyme   3335817   3336459   +
lipoprotein, putative   3336458   3336947   +
P2 phage tail completion protein R (GpR)3336943   3337363   +
phage virion morphogenesis protein 3337350   3337797   +

Region 42, 3338118-3359718 (21600):

conserved hypothetical protein3338288   3339458   +
phage-related baseplate assembly protein3339588   3340479   +
phage tail protein I3340507   3341017   +
phage-related protein   3341026   3342532   +
probable tail fiber assembly protein, putative   3342731
3343118   +
phage-related baseplate protein   3343178   3343742   +
phage-related baseplate protein   3343738   3344098   +

phage tail sheath protein3344109   3345276   +
phage major tail tube protein 3345306   3345816   +
phage tail protein E3345861   3346164   +
phage tail protein, P2 GpE family 3346172   3346286   +
phage-related tail protein   3346318   3349189   +
gpU 3349201   3349603   +
bacteriophage P2 gpD protein 3349599   3350586   +
phage-related protei3351193   3351625   -
hypothetical protein3351899   3352043   -
hypothetical protein3352140   3352278   +
N-acetylglucosamine-6-phosphate deacetylase 3352371   3352569
+
conserved hypothetical protein3352565   3352778   +
phage-related protein   3352867   3355471   +
hypothetical protein3355794   3356013   +
conserved hypothetical protein3356009   3356288   +
conserved hypothetical protein3356310   3356550   +
conserved hypothetical protein3356926   3357049   +
conserved hypothetical protein3357062   3357224   +
conserved hypothetical protein3357279   3357489   +
conserved hypothetical protein3357485   3357710   +
site-specific recombinase, phage integrase family 3358612
3359641   +

Region 43, 3433621-3434406 (785):
Region 44, 3504104-3504205 (101):
Region 45, 3614904-3615928 (1024):

conserved hypothetical protein3615059   3615938   -

Region 46, 3617000-3620076 (3076):

HsdS polypeptide, part of CfrA family   3616930   3617386   -
type I restriction enzyme EcoEI M protein   3618315   3619785
-
conserved hypothetical protein3619789   3620512   -

Region 47, 3621276-3623681 (2405):

type I restriction enzyme EcoAI R protein   3621211   3623593
-
plasmid stabilization system 3623671   3623980   -

Region 48, 3630780-3631221 (441):

type I restriction-modification system, S subunit 3630739
3632245   +

Region 49, 3631329-3632006 (677):

type I restriction-modification system, S subunit 3630739 3632245 +

Region 50, 3635312-3635964 (652):

conserved hypothetical protein3635669 3638168 -

Region 51, 3638829-3639466 (637):

conserved hypothetical protein3638169 3638913 -
conserved hypothetical protein3639209 3642071 -

Region 52, 3663727-3665273 (1546):

EF hand domain protein 3661564 3664459 +

Region 53, 3666659-3669552 (2893):

Rhs element Vgr protein 3665293 3666727 +
conserved hypothetical protein3666730 3668986 +
Rhs element Vgr protein 3669547 3671311 +

Region 54, 3706973-3708016 (1043):

conserved hypothetical protein3707228 3707369 +
conserved hypothetical protein, putative3707596 3708313 -

Region 55, 4281001-4281680 (679):

bacterioferritin 4280992 4281553 +

Region 56, 4282628-4282764 (136):
Region 57, 4287089-4287529 (440):

conserved hypothetical protein4286556 4287093 -

Region 58, 4560824-4560991 (167):
Region 59, 4576908-4577317 (409):
Region 60, 4578380-4580468 (2088):

filamentous haemagglutinin, N-terminal:Adhesin HecA 20-residue repeat x2 4578319 4580452 -

Region 61, 4581950-4592534 (10584):

hypothetical protein4581885 4581999 +

radical SAM domain protein   4584952   4585303   +
hypothetical gene   4586742   4587081   +
putative secretion protein   4588131   4589409   +
ABC transporter, ATP-binding protein   4589405   4591409   +
TPR repeat4591773   4592472   +

Region 62, 4593092-4594817 (1725):

integrase core domain protein 4592893   4593595   -
transposase IS34593703   4593997   +
putative transposase4594041   4594854   +

Region 63, 4595672-4595915 (243):
Region 64, 4597233-4597426 (193):

filamentous haemagglutinin   4597299   4599873   -

Region 65, 4621018-4621313 (295):

phosphoribosylamine--glycine ligase4621312   4622602   +

Region 66, 4997966-4998684 (718):
Region 67, 5002968-5004585 (1617):

transposase   5002306   5003038   -
prophage Lp2 protein 6   5003191   5004472   +
bleomycin resistance protein 5004572   5004953   -

Region 68, 5004773-5005139 (366):

bleomycin resistance protein 5004572   5004953   -
putative secreted protein5004959   5005313   -

Regions unique to PXO99A-s when compared to MAFF311018:

Region 0, 31166-32267 (1101):
Region 1, 33130-34546 (1416):

transposase   34517   34727   -

Region 2, 34785-39092 (4307):

conserved hypothetical protein34957   35620   -
LasA 35773   36568   -
hypothetical protein36545   36662   -
conserved hypothetical protein36723   37209   +
proline-betaine transporter   37567   38212   -
proline/betaine transporter   38212   38788   -

Region 3, 117837-119040 (1203):

Rhs family protein 117041 118055 +
ISXo8 transposase 118035 120438 +

Region 4, 137239-137705 (466):
Region 5, 138759-139126 (367):
Region 6, 139339-139702 (363):

conserved hypothetical protein139332 139536 -
hypothetical protein139599 139743 +

Region 7, 197057-197763 (706):

hypothetical protein197681 197795 +

Region 8, 322663-323548 (885):

RecF/RecN/SMC N terminal domain protein 321507 324162 -

Region 9, 914303-914756 (453):

conserved hypothetical protein913963 915043 +

Region 10, 1013381-1013601 (220):

conserved hypothetical protein1013313 1013622 +

Region 11, 1180275-1180814 (539):

two-component system sensor protein1180248 1180515 -
two-component system regulatory protein 1180677 1181292 -

Region 12, 1211325-1214784 (3459):

extracellular protease 1212054 1213419 +
hypothetical protein1213531 1213648 +
extracellular protease 1213777 1215460 +

Region 13, 1327712-1328273 (561):

lipoprotein, putative 1327675 1328635 -

Region 14, 1330893-1331133 (240):

ATPase involved in DNA repair 1330360 1330927 +

Region 15, 1391907-1393468 (1561):

DNA (cytosine-5)-methyltransferase PliMCI 1392095 1393283 +

Region 16, 1394783-1396947 (2164):

conserved hypothetical protein1394830 1395505 +
transposase TnpA, ISL3 family 1395497 1396916 +
ISXoo2 transposase 1396895 1397951 -

Region 17, 1502519-1503203 (684):

hypothetical protein1502715 1502835 +

Region 18, 1556205-1558079 (1874):

RHS Repeat family 1552766 1557506 +
hypothetical protein1557642 1557897 +
RHS Repeat family 1558032 1559271 +

Region 19, 1558996-1560054 (1058):

RHS Repeat family 1558032 1559271 +

Region 20, 1560273-1561255 (982):
Region 21, 1562561-1562873 (312):

transposase 1562601 1562886 +

Region 22, 1577728-1578925 (1197):

conserved hypothetical protein1577773 1577902 -

Region 23, 1582544-1583619 (1075):

conserved hypothetical protein1582370 1582553 -
conserved hypothetical protein1583129 1585964 -

Region 24, 1854942-1855645 (703):
Region 25, 1875166-1875595 (429):
Region 26, 1886072-1886236 (164):
Region 27, 2081447-2081900 (453):
Region 28, 2282355-2283261 (906):

transposase 2281546 2282419 +
hypothetical protein2282449 2282563 -
phosphinothricin N-acetyltransferase 2282619 2283132 +

Region 29, 2286267-2290625 (4358):

conserved hypothetical protein2286235   2286640   -
ATPase, AAA family 2287360   2288338   +
peptidase S8 and S53, subtilisin, kexin, sedolisin2288334
     2290563   +

Region 30, 2293610-2305751 (12141):

cointegrate resolution protein T   2293643   2294663   -
conserved hypothetical protein2294919   2296527   +
conserved hypothetical protein2296673   2297741   +
transcriptional regulator, ArsR family 2297806   2298085   +
arsenate reductase 2298081   2298612   +
arsenate reductase 2298622   2299036   +
arsenical resistance protein ArsH 2299032   2299758   +
arsenical membrane pump 2299776   2301081   +
cointegrase   2301718   2302687   +
transposase   2302696   2305657   +

Region 31, 2374772-2380266 (5494):

hypothetical protein2374987   2375713   +
hypothetical protein2375675   2377568   +
hypothetical protein2377695   2378598   +
hypothetical protein2379713   2379845   -
transposase   2380252   2380465   +

Region 32, 2380483-2380652 (169):
Region 33, 2381637-2382030 (393):
Region 34, 2494219-2494462 (243):

TonB-dependent outer membrane receptor 2491543   2494522   -

Region 35, 2666576-2666930 (354):
Region 36, 2668006-2669087 (1081):

hypothetical protein2667949   2668135   -
RHS Repeat family   2668626   2672664   -

Region 37, 2677253-2679598 (2345):

glutamate synthase domain 2   2673938   2677868   -

Region 38, 3051935-3052780 (845):

peptidase, S54 (rhomboid) family, putative   3051671   3052499

-

Region 39, 3053843-3054091 (248):

hypothetical protein3053865   3053979   +

Region 40, 3054306-3054460 (154):
Region 41, 3054610-3055886 (1276):

hypothetical protein3054726   3054840   -

Region 42, 3169064-3169549 (485):

conserved hypothetical protein3166718   3169553   +

Region 43, 3170375-3170958 (583):

conserved hypothetical protein3170806   3171133   +

Region 44, 3178359-3178950 (591):

conserved hypothetical protein3178944   3179652   +

Region 45, 3317329-3317762 (433):

conserved hypothetical protein3317674   3318283   -

Region 46, 3318480-3319058 (578):

conserved hypothetical protein3318308   3318608   -

Region 47, 3320223-3320724 (501):

conserved hypothetical protein3320233   3323068   -

Region 48, 3324284-3325284 (1000):
Region 49, 3325496-3327090 (1594):

conserved hypothetical protein3325743   3327123   +

Region 50, 3350519-3353867 (3348):

bacteriophage P2 gpD protein 3349599   3350586   +
phage-related protei3351193   3351625   -
hypothetical protein3351899   3352043   -
hypothetical protein3352140   3352278   +
N-acetylglucosamine-6-phosphate deacetylase 3352371   3352569
+

conserved hypothetical protein3352565   3352778   +
phage-related protein   3352867   3355471   +

Region 51, 3356377-3359718 (3341):

conserved hypothetical protein3356310   3356550   +
conserved hypothetical protein3356926   3357049   +
conserved hypothetical protein3357062   3357224   +
conserved hypothetical protein3357279   3357489   +
conserved hypothetical protein3357485   3357710   +
site-specific recombinase, phage integrase family 3358612
3359641   +

Region 52, 3614904-3615934 (1030):

conserved hypothetical protein3615059   3615938   -

Region 53, 3616991-3620076 (3085):

HsdS polypeptide, part of CfrA family   3616930   3617386   -
type I restriction enzyme EcoEI M protein   3618315   3619785
-
conserved hypothetical protein3619789   3620512   -

Region 54, 3621276-3623681 (2405):

type I restriction enzyme EcoAI R protein   3621211   3623593
-
plasmid stabilization system 3623671   3623980   -

Region 55, 3630770-3631221 (451):

type I restriction-modification system, S subunit 3630739
3632245   +

Region 56, 3631329-3632006 (677):

type I restriction-modification system, S subunit 3630739
3632245   +

Region 57, 3661463-3665273 (3810):

Rhs element Vgr protein 3658996   3661540   +
EF hand domain protein   3661564   3664459   +

Region 58, 3666653-3669552 (2899):

Rhs element Vgr protein 3665293   3666727   +

conserved hypothetical protein3666730   3668986   +
Rhs element Vgr protein 3669547   3671311   +

Region 59, 3919101-3919267 (166):
Region 60, 4307055-4308244 (1189):
Region 61, 4547099-4547564 (465):

conserved hypothetical protein4546919   4547114   -

Region 62, 4550264-4550829 (565):

methyltransferase   4550236   4550485   -
methyltransferase   4550496   4552311   -

Region 63, 4557966-4558391 (425):
Region 64, 4576694-4577322 (628):

Rhs family protein 4576675   4576870   -

Region 65, 4578379-4580468 (2089):

filamentous haemagglutinin, N-terminal:Adhesin HecA 20-residue repeat x2 4578319   4580452   -

Region 66, 4581951-4592534 (10583):

hypothetical protein4581885   4581999   +
radical SAM domain protein   4584952   4585303   +
hypothetical gene   4586742   4587081   +
putative secretion protein   4588131   4589409   +
ABC transporter, ATP-binding protein   4589405   4591409   +
TPR repeat4591773   4592472   +

Region 67, 4593092-4594817 (1725):

integrase core domain protein 4592893   4593595   -
transposase IS34593703   4593997   +
putative transposase4594041   4594854   +

Region 68, 4595391-4595915 (524):

filamentous haemagglutinin   4595388   4595544   -

Region 69, 4597233-4612974 (15741):

filamentous haemagglutinin   4597299   4599873   -
hypothetical protein4600150   4600282   +
filamentous haemagglutinin; haemagglutination activity domain

protein   4600321   4610902   -
outer membrane hemolysin activator protein   4611067   4612780   -

Region 70, 4614614-4615451 (837):

ice nucleation protein   4614611   4615442   -

Region 71, 4621018-4621313 (295):

phosphoribosylamine--glycine ligase4621312   4622602   +

Region 72, 4687577-4689034 (1457):

ribonucleoside-diphosphate reductase, beta subunit4689014   4690058   -

Region 73, 5002968-5004585 (1617):

transposase   5002306   5003038   -
prophage Lp2 protein 6   5003191   5004472   +
bleomycin resistance protein   5004572   5004953   -

Region 74, 5004773-5005139 (366):

bleomycin resistance protein   5004572   5004953   -
putative secreted protein5004959   5005313   -

Regions unique to PXO86 when compared to PXO99A-s:

Region 0, 265181-267451 (2270):

hypothetical protein265815   266358   +
hypothetical protein266354   267173   +

Region 1, 268507-269152 (645):

hypothetical protein268678   268858   -

Region 2, 567681-570005 (2324):

deoxyguanosinetriphosphate triphosphohydrolase   567825   569259   +
hypothetical protein569393   569975   +

Region 3, 571073-572386 (1313):

hypothetical protein571075   571174   -

hypothetical protein571225 571540 -
hypothetical protein571532 571832 +
hypothetical protein572218 572446 +

Region 4, 1097427-1098082 (655):

transposase 1097017 1098349 -

Region 5, 1247417-1247518 (101):
Region 6, 1252941-1256785 (3844):

type IV secretion protein Rhs 1250587 1253017 +
hypothetical protein1253031 1253913 +
hypothetical protein1253969 1254374 +
hypothetical protein1254397 1254877 +
hypothetical protein1255383 1256706 +

Region 7, 1273132-1274759 (1627):

hypothetical protein1273154 1273718 -
calcium-binding protein 1273879 1276921 -

Region 8, 1281145-1284130 (2985):

hypothetical protein1281147 1282227 -
hypothetical protein1282223 1283762 -
type IV secretion protein Rhs 1283761 1285999 -

Region 9, 1286213-1288011 (1798):

type IV secretion protein Rhs 1287753 1289322 -

Region 10, 1309979-1310563 (584):

hypothetical protein1307373 1310241 +
hypothetical protein1310245 1311265 +

Region 11, 1313887-1314417 (530):

type I restriction endonuclease StySPI subunit S 1313647 1314529 -

Region 12, 1314525-1314963 (438):

type I restriction endonuclease StySPI subunit S 1313647 1314529 -

Region 13, 1321766-1330185 (8419):

plasmid stabilization protein 1321763   1322072   +
restriction endonuclease 1322124   1323117   +
DNA methyltransferase   1323134   1324949   +
hypothetical protein1324962   1325394   +
restriction endonuclease subunit S 1325386   1326961   +
DEAD/DEAH box helicase   1326960   1330140   +

Region 14, 1360705-1361778 (1073):

outer protein P1360817   1362950   +

Region 15, 1415102-1416080 (978):

hypothetical protein1415221   1415527   +

Region 16, 1417141-1418753 (1612):
Region 17, 1729220-1729365 (145):
Region 18, 1732204-1733325 (1121):

hypothetical protein1729856   1732691   +
hypothetical protein1732712   1733465   +

Region 19, 1736424-1736665 (241):
Region 20, 1738182-1738372 (190):

hypothetical protein1738213   1738387   +

Region 21, 1739464-1740138 (674):

hypothetical protein1739519   1740266   +

Region 22, 2011776-2012669 (893):

ATPase domain protein   2011836   2012835   +

Region 23, 2015163-2015540 (377):

hypothetical protein2015214   2015331   -

Region 24, 2015741-2016449 (708):

hypothetical protein2015589   2016033   +
hypothetical protein2016029   2016446   +
hypothetical protein2016363   2017338   -

Region 25, 2036070-2036285 (215):
Region 26, 2626182-2627314 (1132):

Region 27, 2803830-2804914 (1084):

hypothetical protein2804429   2807264   -

Region 28, 3138308-3138714 (406):

beta-galactosidase 3137641   3138634   +

Region 29, 3337002-3337193 (191):

type IV secretion protein Rhs 3335190   3337044   -

Region 30, 3339354-3339881 (527):
Region 31, 3341048-3341388 (340):

hypothetical protein3341068   3341338   +

Region 32, 3347752-3347947 (195):

type IV secretion protein Rhs 3345485   3347798   -

Region 33, 3349011-3349999 (988):

type IV secretion protein Rhs 3348985   3353455   -

Region 34, 3554866-3557352 (2486):

hypothetical protein3554946   3557055   -

Region 35, 3558518-3561652 (3134):

hypothetical protein3559216   3559849   -
hypothetical protein3560213   3560405   +
hypothetical protein3560757   3561546   -

Region 36, 3563987-3565560 (1573):
Region 37, 3989625-3989798 (173):

hypothetical protein3988755   3990450   -

Region 38, 4506667-4516964 (10297):

endonuclease   4506811   4507609   -
DNA-methyltransferase   4507605   4508880   -
XorII very short patch repair endonuclease   4508960   4509371
-
hypothetical protein4509363   4510035   -
hypothetical protein4510683   4510842   -

hypothetical protein4510838	4511141	-
conjugal transfer protein TraH4511147	4512233	-
conjugal transfer protein TraG4512234	4512495	-
hypothetical protein4512491	4512971	-
type IV secretion protein VirB5	4512984	4513704	-
hypothetical protein4514054	4514300	-
hypothetical protein4514335	4515175	-
integrase 4515643	4516738	-

Region 39, 4575804-4575975 (171):
Region 40, 4577047-4577397 (350):

hemagglutinin	4576441	4579057	-

Region 41, 4600186-4602106 (1920):

ATPase	4600235	4601471	+
hypothetical protein4601470	4602085	+
phosphoribosylamine--glycine ligase4602105	4603395	+

Region 42, 4990249-4990973 (724):

hypothetical protein4990006	4990759	-
hypothetical protein4990755	4991463	-

Regions unique to PXO86 when compared to MAFF311018:

Region 0, 31168-32269 (1101):

hypothetical protein30988	32188	-

Region 1, 33132-34548 (1416):

hypothetical protein33100	33982	-
hypothetical protein34162	34357	-
transposase IS1479	34519	34723	-

Region 2, 34787-39094 (4307):

hypothetical protein34959	35595	-
peptidase 35775	36594	-
ATPase	36737	37211	+
hypothetical protein37431	37521	+

Region 3, 121459-122662 (1203):

type IV secretion protein Rhs 120663	121677	+
DDE endonuclease	121714	123691	+

Region 4, 140860-142048 (1188):

hypothetical protein141943   142087   +

Region 5, 200051-200759 (708):

hypothetical protein200104   200215   +
hypothetical protein200678   200792   +

Region 6, 265180-267441 (2261):

hypothetical protein265814   266357   +
hypothetical protein266353   267172   +

Region 7, 268510-269153 (643):

hypothetical protein268677   268857   -

Region 8, 321686-322048 (362):
Region 9, 323364-323891 (527):
Region 10, 567680-569996 (2316):

deoxyguanosinetriphosphate triphosphohydrolase   567824
569258   +
hypothetical protein569392   569974   +

Region 11, 571072-572385 (1313):

hypothetical protein571074   571173   -
hypothetical protein571224   571539   -
hypothetical protein571531   571831   +
hypothetical protein572217   572445   +

Region 12, 831930-832042 (112):

hypothetical protein831568   832648   +

Region 13, 834423-834876 (453):

hypothetical protein834083   835163   +

Region 14, 934253-934473 (220):

hypothetical protein933939   934494   +

Region 15, 1097420-1098629 (1209):

transposase   1097016   1098348   -
LuxR family transcriptional regulator   1098492   1099200   -

Region 16, 1130513-1133972 (3459):

peptidase S8   1131242   1132607   +
peptidase S8   1132950   1134648   +

Region 17, 1247415-1247516 (101):
Region 18, 1249119-1249319 (200):

DNA repair ATPase   1248586   1249153   +
sulfatase modifying factor 1 1249209   1250166   -

Region 19, 1265512-1265655 (143):

hypothetical protein1265066   1265681   +

Region 20, 1273130-1277021 (3891):

hypothetical protein1273152   1273716   -
calcium-binding protein 1273877   1276919   -
type IV secretion protein Rhs 1276943   1279481   -

Region 21, 1281143-1284124 (2981):

hypothetical protein1281145   1282225   -
hypothetical protein1282221   1283760   -
type IV secretion protein Rhs 1283759   1285997   -

Region 22, 1313885-1314412 (527):

type I restriction endonuclease StySPI subunit S 1313645
1314527   -

Region 23, 1314527-1314959 (432):
Region 24, 1325690-1326200 (510):

restriction endonuclease subunit S 1325384   1326959   +

Region 25, 1326350-1326959 (609):

restriction endonuclease subunit S 1325384   1326959   +
DEAD/DEAH box helicase   1326958   1330138   +

Region 26, 1360703-1361776 (1073):

outer protein P1360815   1362948   +

Region 27, 1415100-1416078 (978):

hypothetical protein1415219   1415525   +

Region 28, 1417140-1418751 (1611):
Region 29, 2011719-2012678 (959):

ATPase domain protein   2011835   2012834   +

Region 30, 2015161-2015396 (235):

hypothetical protein2015213   2015330   -

Region 31, 2032436-2032846 (410):

hypothetical protein2032289   2033024   +

Region 32, 2036076-2036289 (213):
Region 33, 2038932-2039096 (164):

hypothetical protein2038899   2039199   +

Region 34, 2236339-2236792 (453):

methylamine utilization protein   2236335   2236497   -

Region 35, 2238405-2240280 (1875):

hypothetical protein2238529   2238727   +
hypothetical protein2239982   2244059   +

Region 36, 2248885-2249671 (786):

type IV secretion protein Rhs 2244088   2249371   +

Region 37, 2415873-2416116 (243):

membrane protein   2415812   2418791   +

Region 38, 2525577-2525963 (386):
Region 39, 2526946-2527118 (172):
Region 40, 2527334-2532625 (5291):

transposase   2527134   2527338   -
hypothetical protein2527760   2527886   +
hypothetical protein2528003   2528162   +
hypothetical protein2529001   2529754   -

hypothetical protein2530031   2531924   -
hypothetical protein2531886   2532612   -

Region 41, 2597125-2609266 (12141):

transposase IS630   2596897   2597161   -
transposase Tn5044 2597218   2600179   -
integrase 2600188   2601157   -
sulfurtransferase   2601174   2601705   -
arylsulfatase 2601794   2603099   -
NADPH-dependent FMN reductase 2603117   2603843   -
arsenate reductase 2603839   2604253   -
ArsR family transcriptional regulator   2604263   2604794   -
ArsR family transcriptional regulator   2604790   2605138   -
conjugal transfer protein2606348   2607929   -
transposase   2608212   2609232   +

Region 42, 2612246-2616610 (4364):

peptidase S8 and S53, subtilisin, kexin, sedolisin2612304   2614533   -
ATPase   2614529   2615693   -
hypothetical protein2616227   2616632   +

Region 43, 2619608-2620513 (905):

phosphinothricin acetyltransferase 2619735   2620254   -
DDE endonuclease   2620448   2621321   -

Region 44, 2799604-2799727 (123):
Region 45, 2800786-2801794 (1008):

hypothetical protein2800723   2801281   -
hypothetical protein2801303   2803646   -

Region 46, 3215654-3216695 (1041):
Region 47, 3217752-3218971 (1219):
Region 48, 3337002-3337187 (185):

type IV secretion protein Rhs 3335190   3337044   -

Region 49, 3339351-3339881 (530):
Region 50, 3341057-3341386 (329):

hypothetical protein3341068   3341338   +

Region 51, 3347756-3347941 (185):

type IV secretion protein Rhs 3345485   3347798   -

Region 52, 3349004-3349999 (995):

type IV secretion protein Rhs 3348985   3353455   -

Region 53, 3354205-3354517 (312):

transposase   3354191   3354587   -

Region 54, 3355823-3357861 (2038):

hypothetical protein3356043   3356619   -
type IV secretion protein Rhs 3356622   3361317   -

Region 55, 3410884-3411571 (687):

hypothetical protein3410958   3411453   -

Region 56, 3812567-3812733 (166):
Region 57, 4202141-4203330 (1189):
Region 58, 4443966-4444431 (465):

hypothetical protein4443780   4443975   -

Region 59, 4447983-4448548 (565):
Region 60, 4455686-4456108 (422):

hypothetical protein4455601   4455931   +

Region 61, 4506674-4516971 (10297):

endonuclease   4506812   4507610   -
DNA-methyltransferase   4507606   4508881   -
XorII very short patch repair endonuclease   4508961   4509372   -
hypothetical protein4509364   4510036   -
hypothetical protein4510684   4510843   -
hypothetical protein4510839   4511142   -
conjugal transfer protein TraH4511148   4512234   -
conjugal transfer protein TraG4512235   4512496   -
hypothetical protein4512492   4512972   -
type IV secretion protein VirB5   4512985   4513705   -
hypothetical protein4514055   4514301   -
hypothetical protein4514336   4515176   -
integrase 4515644   4516739   -

Region 62, 4573037-4573245 (208):

hypothetical protein4573012 4573207 -

Region 63, 4575811-4575989 (178):
Region 64, 4577053-4592164 (15111):

hemagglutinin 4576442 4579058 -
hypothetical protein4579146 4579239 -
hemagglutinin 4579506 4590231 -
membrane protein 4590252 4591995 -

Region 65, 4593792-4594628 (836):

ice nucleation protein 4593780 4594611 -

Region 66, 4668394-4669851 (1457):

hypothetical protein4668593 4669085 -
hypothetical protein4669084 4669783 -
ribonucleotide-diphosphate reductase subunit beta 4669825 4670869 -

Regions unique to MAFF311018 when compared to PXO99A-s:

Region 0, 113109-113251 (142):
Region 1, 216039-216281 (242):

conserved hypothetical protein215452 217144 +

Region 2, 483227-484150 (923):

ribonucleoside-diphosphate reductase beta chain 482202 483246 +
hypothetical protein483296 484136 +
conserved hypothetical protein484149 484644 +

Region 3, 550442-552362 (1920):

conserved hypothetical protein550462 551077 -
conserved hypothetical protein551076 552312 -
bifunctional purine biosynthesis protein552361 553945 -

Region 4, 580555-580655 (100):

conserved hypothetical protein580337 580814 -

Region 5, 634590-635574 (984):

conserved hypothetical protein634633 634798 +
putative Zn-dependent alcohol dehydrogenase 634882 635569
-

Region 6, 882009-882395 (386):

hypothetical protein882181 882655 +

Region 7, 1072335-1073012 (677):

conserved hypothetical protein1072350 1072989 -

Region 8, 1074184-1074931 (747):

conserved hypothetical protein1074161 1074581 -
hypothetical protein1074577 1074793 -

Region 9, 1076417-1076729 (312):

hypothetical protein1076372 1076708 -

Region 10, 1461096-1461982 (886):

hypothetical protein1460468 1461119 +
hypothetical protein1461045 1461996 +

Region 11, 1463154-1463829 (675):

hypothetical protein1463189 1463681 +
VGR-related protein 1463690 1466114 +

Region 12, 1466164-1468656 (2492):

conserved hypothetical protein1466110 1467058 +
hypothetical protein1467050 1467401 +
conserved hypothetical protein1468153 1468600 +

Region 13, 1469488-1472607 (3119):

conserved hypothetical protein1469696 1470368 +
conserved hypothetical protein1470417 1472526 +
VGR-related protein 1472547 1474449 +

Region 14, 1595555-1596238 (683):

putative Rhs-related protein 1596199 1597708 +

Region 15, 1618823-1619807 (984):

conserved hypothetical protein1619086   1619383   +

Region 16, 1711773-1716531 (4758):

hypothetical protein1712100   1712505   +
conserved hypothetical protein1712510   1712660   +
pseudouridylate synthase 1712949   1713660   -
conserved hypothetical protein1713894   1714101   +
conserved hypothetical protein1714304   1714733   +
conserved hypothetical protein1714736   1715297   +
membrane transport protein   1715894   1716467   -

Region 17, 1723728-1726904 (3176):

phage-related integrase 1723803   1724988   -
conserved hypothetical protein1724987   1725248   -
conserved hypothetical protein1725205   1725412   -
conserved hypothetical protein1725408   1725681   -
conserved hypothetical protein1725919   1726195   -
conserved hypothetical protein1726187   1726343   -
conserved hypothetical protein1726356   1726767   -

Region 18, 1729399-1732816 (3417):

phage-related protein   1727794   1730491   -
conserved hypothetical protein1730500   1730713   -
hypothetical protein1730709   1730988   -
conserved hypothetical protein1730998   1731319   -
phage-related protein   1731650   1732088   +
phage-related tail protein   1732748   1733735   -

Region 19, 1758441-1761268 (2827):

polymerase V subunit1759263   1760550   -
conserved hypothetical protein1760777   1761113   +

Region 20, 1836091-1838660 (2569):

conserved hypothetical protein1836656   1837775   +
conserved hypothetical protein1837771   1838443   +

Region 21, 1838874-1838995 (121):
Region 22, 1915133-1915539 (406):

conserved hypothetical protein1915212   1916019   -

Region 23, 1970105-1970736 (631):

conserved hypothetical protein1970101   1970752   -

Region 24, 2170655-2171004 (349):

conserved hypothetical protein2170618   2170966   +

Region 25, 2370527-2376274 (5747):

TrbP protein   2370590   2371238   -
phage-related protein   2371239   2372427   -
conserved hypothetical protein2372426   2372756   -
phage-related protein   2372755   2374204   -
phage-related protein   2374298   2374529   -
V protein 2374747   2375044   -
replication initiation protein2375040   2376081   -
hypothetical protein2376233   2376446   -

Region 26, 2376634-2377152 (518):

18.2K protein 2376716   2377388   +

Region 27, 2378982-2381000 (2018):

hypothetical protein2379596   2379788   +
hypothetical protein2380020   2380488   +
hypothetical protein2380401   2380914   -

Region 28, 2381823-2382347 (524):
Region 29, 2448753-2449886 (1133):

hypothetical protein2448780   2449662   -
hypothetical protein2449786   2449954   +

Region 30, 2984365-2985455 (1090):

conserved hypothetical protein2984964   2987307   -

Region 31, 2987503-2988593 (1090):

hypothetical protein2987331   2988084   -
conserved hypothetical protein2988105   2990448   -

Region 32, 2991166-2991733 (567):

hypothetical protein2990472   2991207   -
conserved hypothetical protein2991235   2994070   -

Region 33, 3007524-3008234 (710):

hypothetical protein3006633 3007608 +
conserved hypothetical protein3007525 3007942 -
conserved hypothetical protein3007938 3008190 -

Region 34, 3008435-3010021 (1586):

conserved hypothetical protein3008627 3009263 +

Region 35, 3011185-3012531 (1346):

hypothetical protein3011169 3011778 -
hypothetical protein3011774 3012089 -

Region 36, 3293898-3294967 (1069):

hypothetical protein3293721 3294459 -
conserved hypothetical protein3294489 3296832 -

Region 37, 3297009-3298127 (1118):

conserved hypothetical protein3297624 3300678 -

Region 38, 3300951-3301096 (145):
Region 39, 3695000-3700214 (5214):

type I restriction-modification system endonuclease 3695044 3698224 -
hypothetical protein3698223 3698898 -
conserved hypothetical protein3698897 3699644 -
hypothetical protein3699640 3700174 -

Region 40, 3701386-3701611 (225):
Region 41, 3702777-3707093 (4316):

conserved hypothetical protein3702758 3703472 -
nucleotidyltransferase 3703464 3703899 -
type I restriction system adenine methylase 3703909 3705724 -
conserved hypothetical protein3705741 3706734 -
conserved hypothetical protein3706786 3707095 -

Region 42, 3713885-3714315 (430):

specificity determinant for hsdM and hsdR 3713869 3715222 +

Region 43, 3714423-3714983 (560):

specificity determinant for hsdM and hsdR 3713869 3715222 +

Region 44, 3718295-3718817 (522):

hypothetical protein3717605 3718442 -

Region 45, 3725781-3726365 (584):

conserved hypothetical protein3725078 3726098 -
hypothetical protein3726102 3728046 -

Region 46, 3731623-3732251 (628):

conserved hypothetical protein3730944 3731970 -
conserved hypothetical protein3731973 3734841 -

Region 47, 3754206-3756004 (1798):

putative VGR-related protein 3751745 3754463 +
putative VGR-related protein 3755995 3757966 +

Region 48, 3769483-3774632 (5149):

conserved hypothetical protein3769561 3770884 -
conserved hypothetical protein3771390 3771870 -
conserved hypothetical protein3771893 3772358 -
conserved hypothetical protein3772674 3773154 -
conserved hypothetical protein3773177 3773642 -
conserved hypothetical protein3773638 3774535 -
putative VGR-related protein 3774534 3777351 -

Region 49, 4214633-4217872 (3239):

restriction endonuclease homolog R.XphI 4214731 4215826 -
methyltransferase homolog M.XphI 4215822 4217613 -

Region 50, 4231764-4232217 (453):

hypothetical protein4231476 4232556 -

Region 51, 4903040-4903304 (264):

conserved hypothetical protein4902738 4903095 +

Region 52, 4913841-4914565 (724):

hypothetical protein4914347   4915055   -

Regions unique to MAFF311018 when compared to PXO86:

Region 0, 113108-113251 (143):
Region 1, 483227-484150 (923):

ribonucleoside-diphosphate reductase beta chain   482202
483246   +
hypothetical protein483296   484136   +
conserved hypothetical protein484149   484644   +

Region 2, 572565-572728 (163):
Region 3, 580555-580655 (100):

conserved hypothetical protein580337   580814   -

Region 4, 634590-635574 (984):

conserved hypothetical protein634633   634798   +
putative Zn-dependent alcohol dehydrogenase 634882   635569
-

Region 5, 882009-882395 (386):

hypothetical protein882181   882655   +

Region 6, 903209-903655 (446):

hypothetical protein903650   904187   +

Region 7, 905812-905944 (132):

ISXoo15 transposase 905921   906884   +

Region 8, 906892-907570 (678):

bacterioferritin   907017   907578   -

Region 9, 1072335-1073012 (677):

conserved hypothetical protein1072350   1072989   -

Region 10, 1074181-1074933 (752):

conserved hypothetical protein1074161   1074581   -
hypothetical protein1074577   1074793   -

Region 11, 1076416-1076729 (313):

hypothetical protein1076372   1076708   -

Region 12, 1427414-1428457 (1043):

conserved hypothetical protein1427176   1427833   +
hypothetical protein1427885   1428440   -
DNA-3-methyladenine glycosylase I 1428456   1429029   -

Region 13, 1517127-1517763 (636):

conserved hypothetical protein1516891   1517479   +

Region 14, 1589553-1591138 (1585):

putative Rhs-related protein 1589425   1593916   +

Region 15, 1595557-1596238 (681):

putative Rhs-related protein 1596199   1597708   +

Region 16, 1597138-1598122 (984):

putative Rhs-related protein 1596199   1597708   +
hypothetical protein1597713   1598082   +

Region 17, 1618823-1619807 (984):

conserved hypothetical protein1619086   1619383   +

Region 18, 1673679-1675424 (1745):

conserved hypothetical protein1670222   1674806   +

Region 19, 1711773-1716533 (4760):

hypothetical protein1712100   1712505   +
conserved hypothetical protein1712510   1712660   +
pseudouridylate synthase 1712949   1713660   -
conserved hypothetical protein1713894   1714101   +
conserved hypothetical protein1714304   1714733   +
conserved hypothetical protein1714736   1715297   +
membrane transport protein   1715894   1716467   -

Region 20, 1723728-1745221 (21493):

phage-related integrase 1723803   1724988   -
conserved hypothetical protein1724987   1725248   -
conserved hypothetical protein1725205   1725412   -
conserved hypothetical protein1725408   1725681   -
conserved hypothetical protein1725919   1726195   -
conserved hypothetical protein1726187   1726343   -
conserved hypothetical protein1726356   1726767   -
conserved hypothetical protein1726992   1727271   -
hypothetical protein1727267   1727486   -
phage-related protein   1727794   1730491   -
conserved hypothetical protein1730500   1730713   -
hypothetical protein1730709   1730988   -
conserved hypothetical protein1730998   1731319   -
phage-related protein   1731650   1732088   +
phage-related tail protein   1732748   1733735   -
phage-related tail protein   1733731   1734133   -
phage-related tail protein   1734145   1737016   -
phage-related protein   1737048   1737162   -
phage-related tail protein   1737170   1737473   -
phage-related tail protein   1737518   1738028   -
phage-related tail protein   1738058   1739225   -
phage-related baseplate protein   1739236   1739596   -
phage-related baseplate protein   1739592   1740156   -
hypothetical protein1740216   1740795   -
hypothetical protein1740802   1742308   -
phage-related tail protein   1742317   1742863   -
phage-related baseplate assembly protein1742855   1743746   -
conserved hypothetical protein1743876   1745046   -

Region 21, 1745447-1755228 (9781):

phage-related tail protein   1745537   1745984   -
phage-related tail protein   1745971   1746391   -
conserved hypothetical protein1746387   1746876   -
phage-related lytic enzyme   1746875   1747514   -
phage-related protein   1747513   1747789   -
phage-related protein   1747781   1748138   -
phage-related tail protein   1748142   1748352   -
phage-related capsid completion protein 1748351   1748819   -
phage-related terminase 1748918   1749638   -
phage-related major capsid protein 1749641   1750658   -
phage-related capsid scaffold protein   1750704   1751547   -
phage-related terminase 1751668   1753453   +
phage-related capsid packaging protein 1753452   1754475   +
site-specific DNA-methyltransferase1754663   1755365   +

Region 22, 1755674-1756392 (718):

conserved hypothetical protein1755431   1756178   -
conserved hypothetical protein1756174   1756882   -

Region 23, 1758441-1761268 (2827):

polymerase V subunit1759263   1760550   -
conserved hypothetical protein1760777   1761113   +

Region 24, 1832676-1832817 (141):

hypothetical protein1832552   1832855   +

Region 25, 1836091-1838655 (2564):

conserved hypothetical protein1836656   1837775   +
conserved hypothetical protein1837771   1838443   +

Region 26, 1838880-1839224 (344):

ISXoo13 transposase 1839214   1840474   -

Region 27, 1970105-1970736 (631):

conserved hypothetical protein1970101   1970752   -

Region 28, 2170655-2171004 (349):

conserved hypothetical protein2170618   2170966   +

Region 29, 2195178-2195444 (266):
Region 30, 2362043-2369955 (7912):

phage-related protein   2361980   2362403   -
phage-related protein   2363067   2364141   +
phage-related protein   2364245   2364545   +
hypothetical protein2364908   2365148   +
phage-related protein   2365284   2366739   +
phage-related protein   2367057   2368242   +
hypothetical protein2368530   2368932   +
hypothetical protein2368871   2369186   -

Region 31, 2370311-2377137 (6826):

hypothetical protein2370035   2370419   +
TrbP protein   2370590   2371238   -
phage-related protein   2371239   2372427   -
conserved hypothetical protein2372426   2372756   -
phage-related protein   2372755   2374204   -

phage-related protein 2374298 2374529 -
V protein 2374747 2375044 -
replication initiation protein2375040 2376081 -
hypothetical protein2376233 2376446 -
hypothetical protein2376445 2376631 -
18.2K protein 2376716 2377388 +

Region 32, 2378982-2380996 (2014):

hypothetical protein2379596 2379788 +
hypothetical protein2380020 2380488 +
hypothetical protein2380401 2380914 -

Region 33, 2381824-2382347 (523):
Region 34, 2394766-2394962 (196):
Region 35, 2396023-2396206 (183):
Region 36, 2400506-2402581 (2075):

RhsD protein 2398397 2402498 -

Region 37, 2765236-2765444 (208):
Region 38, 2768734-2769712 (978):

conserved hypothetical protein2769267 2774103 -

Region 39, 2835395-2835589 (194):
Region 40, 2981257-2982213 (956):

conserved hypothetical protein2981840 2984183 -

Region 41, 2990614-2990815 (201):

hypothetical protein2990472 2991207 -

Region 42, 2991164-2991734 (570):

hypothetical protein2990472 2991207 -
conserved hypothetical protein2991235 2994070 -

Region 43, 3008578-3010019 (1441):

conserved hypothetical protein3008627 3009263 +

Region 44, 3011186-3012542 (1356):

hypothetical protein3011169 3011778 -
hypothetical protein3011774 3012089 -

Region 45, 3293881-3294967 (1086):

hypothetical protein3293721   3294459   -
conserved hypothetical protein3294489   3296832   -

Region 46, 3510363-3511154 (791):

conserved hypothetical protein3510415   3511591   -

Region 47, 3579083-3579189 (106):
Region 48, 3698224-3700207 (1983):

hypothetical protein3698223   3698898   -
conserved hypothetical protein3698897   3699644   -
hypothetical protein3699640   3700174   -

Region 49, 3702777-3703167 (390):

conserved hypothetical protein3702758   3703472   -

Region 50, 3713898-3714311 (413):

specificity determinant for hsdM and hsdR   3713869   3715222
+

Region 51, 3714426-3714983 (557):

specificity determinant for hsdM and hsdR   3713869   3715222
+

Region 52, 3718304-3718815 (511):

hypothetical protein3717605   3718442   -

Region 53, 3719882-3719985 (103):

hypothetical protein3719839   3722278   -

Region 54, 3722849-3723486 (637):

conserved hypothetical protein3722189   3723221   -
hypothetical protein3723229   3725167   -

Region 55, 3728632-3729284 (652):

conserved hypothetical protein3727957   3728980   -
hypothetical protein3728989   3730960   -

```
Region 56, 3731631-3732269 (638):

conserved hypothetical protein3730944   3731970   -
conserved hypothetical protein3731973   3734841   -

Region 57, 4214633-4217872 (3239):

restriction endonuclease homolog R.XphI 4214731   4215826   -
methyltransferase homolog M.XphI   4215822   4217613   -

Region 58, 4227360-4228411 (1051):

conserved hypothetical protein4226933   4228208   -

Region 59, 4232084-4232195 (111):

hypothetical protein4231476   4232556   -

Region 60, 4517548-4519406 (1858):

conserved hypothetical protein4517580   4518315   -
conserved hypothetical protein4518311   4519205   -

Region 61, 4815304-4816015 (711):

putative type III effector HopPtoH like protein   4815531
     4815999   -

Region 62, 4817185-4817632 (447):
Region 63, 4903040-4903304 (264):

conserved hypothetical protein4902738   4903095   +
```

References

Kurtz, S., Phillippy, A., Delcher, A. L., Smoot, M., Shumway, M., Antonescu, C. & Salzberg, S. L. (2004). Versatile and open software for comparing large genomes. *Genome Biol* **5**.

Quinlan, A. R. & Hall, I. M. (2010). BEDTools: a flexible suite of utilities for comparing genomic features. *Bioinformatics* **26**, 841-842.
