# Supplementary material for: Single molecule real-time sequencing of Xanthomonas oryzae genomes reveals a dynamic structure and complex TAL (transcription activator-like) effector gene relationships
Source: Microb Genom. 2015 Oct 30;1(4):e000032. doi: 10.1099/mgen.0.000032 (PMC4853030; doi:10.1099/mgen.0.000032)
Supplement: Supplementary file 6 — Supplementary Data [file mgen-01-32-s006.pdf]

# Supplementary Material
# for

# SMRT SEQUENCING OF *XANTHOMONAS ORYZAE* GENOMES REVEALS A DYNAMIC STRUCTURE AND COMPLEX TAL EFFECTOR GENE RELATIONSHIPS

**Nicholas J. Booher[1], Sara C. D. Carpenter[1], Robert P. Sebra[2], Li Wang[1], Steven L. Salzberg[3], Jan E. Leach[4], and Adam J. Bogdanove[1]***
**Address: [1] Plant Pathology and Plant-Microbe Biology Section, School of Integrative Plant Science, Cornell University, Ithaca, NY 14853 USA; [2] Icahn Institute for Genomics and Multiscale Biology and Department of Genetics & Genomic Sciences, Icahn School of Medicine at Mount Sinai, New York, NY 10029 USA; [3] Departments of Biomedical Engineering, Computer Science, and Biostatistics and Center for Computational Biology, Johns Hopkins University, Baltimore, MD 21205 USA; [4] Bioagricultural Sciences and Pest Management, Colorado State University, Ft. Collins, CO 80523 USA**
***Corresponding author: ajb7@cornell.edu**

**File S6. Alignment of *tal* gene 5' ends that have five codon deviations.** Sequences of the 5' ends of the archetypal *X. euvesicatoria avrBs3* gene, the BLS256 *tal2g* gene (representing Xoc *tal* genes), and *avrXa27* (representing Xoo *tal* genes) are shown for comparison. The sequence duplicated in CFBP7342 *tal8* is highlighted in aqua, and the second copy is highlighted in magenta. These sequences are preceded by a variant (yellow) that is present also in the representative *tal* genes including *avrXa27*, but absent from the other three Xoo *tal* genes. Genbank accessions for sequences included in the alignment are X16130.1 for *avrBs3*, CP003057.1 for BLS256, CP000967.1 for PXO99A, CP007166.1 for PXO86, CP007221.1 for CFBP7342, and AP008229.1 for MAFF311018.

```
avrBs3                  ATGGATCCCATTCGTTCGCGCACACCAAGTCCTGCCCGCGAGCTTCTGCCCGGACCCCAA 60
bls256_tal2g            ATGGATCCCATTCGTCCGCGCAGGCCAAGTCCTGCCCGCGAGCCTCTGCCCGGACCCCAA 60
avrXa27                 ATGGATCCCATTCGTTCGCGCACGCCAAGTCCTGCCCGCGAGCTTCTGCCCGGACCCCAA 60
pxo99a_5a               ATGGATCCCATTCGTTCGCGTACGCCAAGTCCTGCCCGCGAGCTTCTGCCCGGACCCCAA 60
pxo86_tal5a             ATGGATCCCATTCGTTCGCGTACGCCAAGTCCTGCCCGCGAGCTTCTGCCCGGACCCCAA 60
maff311018_tal3a        ATGGATCCCATTCGTTCGCGTACGCCAAGTCCTGCCCGCGAGCTTCTGCCCGGACCCCAA 60
cfbp7342_tal8           ATGGATCCCATTCGTCCGCGCAGGCCAAGTCCTGCCCGCGAGCTTCTGCCCGGACCCCAA 60
                        *************** **** *  ******************* ****************

avrBs3                  CCCGATGGGGTTCAGCCGACTGCAGATCGTGGGGTGTCTCCGCCTGCCGGCGGCCCCCTG 120
bls256_tal2g            CCGGATGGGGTTCAGCCGACTGCAGATCGGGGGGTGTCTGCGCCTGCTGGCGGCCCTCTG 120
avrXa27                 CCGGATAGGGTTCAGCCGACTGCAGATCGGGGGGGGGCTCCGCCTGCTGGCGGCCCCCTG 120
pxo99a_5a               CCGGATAGGGTTCAGCCGACTGCAGATCGGGGGGGGGCTCCGCCTGCTGGCGGCCCCCTG 120
pxo86_tal5a             CCGGATAGGGTTCAGCCGACTGCAGATCGGGGGGGGGCTCCGCCTGCTGGCGGCCCCCTG 120
maff311018_tal3a        CCGGATAGGGTTCAGCCGACTGCAGATCGGGGGGGGGCTCCGCCTGCTGGCGGCCCCCTG 120
cfbp7342_tal8           CCGGATGGGGTTCAGCCGACTGCAGATCGTGGGGTGTCTGCGCCTGCTGGCAGCCCTCTG 120
                        ** *** ********************** **** * ** ******* *** **** ***

avrBs3                  GATGGCTTGCCCGCTCGGCGGACGATGTCCCGGACCCGGCTGCCATCTCCCCCTGCCCCC 180
bls256_tal2g            GATGGTTTGCCCGCTCGGCGGACGATGTCCCGGACCCGGCTGCCATCTCCCCCTGCGCCC 180
avrXa27                 GATGGCTTGCCCGCTCGGCGGACGATGTCCCGGACCCGGCTGCCATCTCCCCCTGCGCCC 180
pxo99a_5a               GATGGCTTGCCCGCTCGGCGGACGATGTCCCGGACCCGGCTGCCATCTCCCCCTGCGCCC 180
pxo86_tal5a             GATGGCTTGCCCGCTCGGCGGACGATGTCCCGGACCCGGCTGCCATCTCCCCCTGCGCCC 180
maff311018_tal3a        GATGGCTTGCCCGCTCGGCGGACGATGTCCCGGACCCGGCTGCCATCTCCCCCTGCGCCC 180
cfbp7342_tal8           GATGGCTTGCCCGCTCGGCGGACGATGTCCCGGACCCGGCTGCCATCTCCCCCTGCGCCC 180
                        ***** ************************************************** ***

avrBs3                  TCACCTGCGTTCTCGGCGGGCAGCTTCAGTGACCTGTTACGTCAGTTCGATCCGTCACTT 240
```

```
bls256_tal2g        TCGCCTGCGTTCTCGGCGGGCAGCTTCAGCGATCTGCTCCGTCCGTTCGATCCGTCGCTT 240
avrXa27             TCGCCTGCGTTCTCGGCGGGCAGCTTCAACGATCTGCTCCGTCAGTTCGATCCGTCGCTT 240
pxo99a_5a           TCGCCTGCGTTCCCGGCGGGCAGCTTCAGCGATCTACTCCGTCAAGTC------------ 228
pxo86_tal5a         TCGCCTGCGTTCCCGGCGGGCAGCTTCAGCGATCTACTCCGTCAAGTC------------ 228
maff311018_tal3a    TCGCCTGCGTTCCCGGCGGGCAGCTTCAGCGATCTGCTCCGTCAAGTC------------ 228
cfbp7342_tal8       TCGCCTGCGTTCTCGGCGGGCAGCTTCAGCGATCTGCTCCGTCCGTTCGATCCGTCGCTT 240
                    ** ********* ***************  ** **   * ****   **            

avrBs3              TTTAATACATCGCTTTTT---------------GATTCATTGCCTCCCTTCGGCGCTCAC 285
bls256_tal2g        CTTGATACATCGCTTCTT---------------GATTCGATGCCTGCCGTCGGCACGCCG 285
avrXa27             CTTGATACATCGCTTCTT---------------GATTCGATGCCTGCCGTCGGCACGCCG 285
pxo99a_5a           ---GATACATCGCTTCTT---------------GATTCGATGCCTGCCGTCGGCACGCCG 270
pxo86_tal5a         ---GATACATCGCTTCTT---------------GATTCGATGCCTGCCGTCGGCACGCCG 270
maff311018_tal3a    ---GATACATCGCTTCTT---------------GATTCGATGCCTGCCGTCGGCACGCCG 270
cfbp7342_tal8       CTTGATACATCGCTTCTTGATACATCGCTTCTTGATTCGATGCCTGCCGTCGGCACGCCG 300
                        *********** **               *****  ***** ** ***** * *  

avrBs3              CATACAGAGGCTGCCACAGGCGAGTGGGATGAGGTGCAATCGGGTCTGCGGGCAGCCGAC 345
bls256_tal2g        CATACAGCGGCTGCCCCAGCAGAGTGGGATGAGGCGCAATCGGGTCTGCGTGCAGCCGAT 345
avrXa27             CATACAGCGGCTGCCCCAGCAGAGTGGGATGAGGTGCAATCGGGTCTGCGTGCAGCCGAT 345
pxo99a_5a           CATACAGCGGCTGCCCCAGCAGAGTGGGATGAGATGCAATCGGGTCTGCGTGCAGCCGAT 330
pxo86_tal5a         CATACAGCGGCTGCCCCAGCAGAGTGGGATGAGATGCAATCGGGTCTGCGTGCAGCCGAT 330
maff311018_tal3a    CATACAGCGGCTGCCCCAGCAGAGTGGGATGAGATGCAATCGGGTCTGCGTGCAGCCGAT 330
cfbp7342_tal8       CATACAGCGGCTGCCCCAGCAGAGTGGGATGAGGTGCAATCGGCTCTGCGTGCAGCCGAT 360
                    ******* ******* ***  ************  ******** ****** ******** 

avrBs3              GCCCCCCCACCCACCATGCGCGTGGCTGTCACTGCCGCGCGGCCGCCGCGCGCCAAGCCG 405
bls256_tal2g        GACCCGCCACCCACCGTGCGTGTCGCTGTCACTGCCGCGCGGCCGCCGCGCGCCAAGCCG 405
avrXa27             GACCCGCCACCCACCGTGCGTGTCGCTGTCACTGCCGCGCGGCCGCCGCGCGCCAAGCCG 405
pxo99a_5a           GACCCGCCACCCACCGTGCGTGTCGCTGTCACTGCCGCGCGGCCGCCGCGCGCCAAGCCG 390
pxo86_tal5a         GACCCGCCACCCACCGTGCGTGTCGCTGTCACTGCCGCGCGGCCGCCGCGCGCCAAGCCG 390
maff311018_tal3a    GACCCGCCACCCACCGTGCGTGTCGCTGTCACTGCCGCGCGGCCGCCGCGCGCCAAGCCG 390
cfbp7342_tal8       GACCCGCCACCCACCGTGCGTGTCGCTGTCACTGCCGCGCGGCCGCCGCGCGCCAAGCCG 420
                    * *** ********* **** ** ************************************

avrBs3              GCGCCGCGACGACGTGCTGCGCAACCCTCCGACGCTTCGCCGGCCGCGCAGGTGGATCTA 465
bls256_tal2g        GCCCCGCGACGGCGTGCGGCGCAACCCTCCGACGCTTCGCCGGCCGCGCAGGTGGATCTA 465
avrXa27             GCCCCGCGACGGCGTGCGGCGCAACCCTCCGACGCTTCGCCGGCCGCGCAGGTGGATCTA 465
pxo99a_5a           GCCCCGCGACGGCGTGCGGCGCAACCCTCCGACGCTTCGCCGGCCGCGCAGGTGGATCTA 450
pxo86_tal5a         GCCCCGCGACGGCGTGCGGCGCAACCCTCCGACGCTTCGCCGGCCGCGCAGGTGGATCTA 450
maff311018_tal3a    GCCCCGCGACGGCGTGCGGCGCAACCCTCCGACGCTTCGCCGGCCGCGCAGGTGGATCTA 450
cfbp7342_tal8       GCCCCGCGACGGCGTGCTGCGCAACCCTCCGACGCTTCGCCGGCCGCGCAGGTGGATCTA 480
                    ** ******** ***** ******************************************

avrBs3              CGCACGCTCGGCTACAGCCAGCAGCAACAGGAGAAGATCAAACCGAAGGTTCGTTCGACA 525
bls256_tal2g        AGCACGCTCGGCTACAGTCAGCAGCAGCAAGAGAAGATCAAACCGAATGTGCGTTCGACA 525
avrXa27             CGCACGCTCGGCTACAGTCAGCAGCAGCAAGAGAAGATCAAATCGAAGGTGCGTTCGACA 525
pxo99a_5a           CGCACGCTCGGCTACAGTCAGCAGCAGCAAGAGAAGATCAAACCGAAGGTGCGTTCGACA 510
pxo86_tal5a         CGCACGCTCGGCTACAGTCAGCAGCAGCAAGAGAAGATCAAACCGAAGGTGCGTTCGACA 510
maff311018_tal3a    CGCACGCTCGGCTACAGTCAGCAGCAGCAAGAGAAGATCAAACCGAAGGTGCGTTCGACA 510
cfbp7342_tal8       CGCACGCTCGGCTACAGTCAGCAGCAGCAAGAGAAGATCGAACCGAATGTGCGTTCGACA 540
                     **************** ******** ** ********* ** **** ** *********

avrBs3              GTGGCGCAGCACCACGAGGCACTGGTCGGCCATGGGTTTACACACGCGCACATCGTTGCG 585
bls256_tal2g        GTGGCGCAGCACCACGAGGCACTGGTGGGCCATGGGTTTACACACGCGCACATCGTTGCG 585
avrXa27             GTGGCGCAGCACCACGAGGCACTGGTGGGCCATGGGTTTACACACGCGCACATCGTTGCG 585
pxo99a_5a           GTGGCGCAGCACCACGAGGCACTGGTGGGCCATGGGTTTACACACGCGCACATCGTTGCG 570
pxo86_tal5a         GTGGCGCAGCACCACGAGGCACTGGTGGGCCATGGGTTTACACACGCGCACATCGTTGCG 570
maff311018_tal3a    GTGGCGCAGCACCACGAGGCACTGGTGGGCCATGGGTTTACACACGCGCACATCGTTGCG 570
cfbp7342_tal8       GTGGCGCAGCACCACGAGGCACTGGTGGGCCATGGGTTTACACACGCGCACATCGTTGCG 600
                    ************************** *********************************

avrBs3              CTCAGCCAACACCCGGCAGCGTTAGGGACCGTCGCTGTCAAGTATCAGGACATGATCGCA 645
```

```
bls256_tal2g          CTCAGCCAACACCCGGCAGCGTTAGGGACCGTTGCTGTCACGTATCAGCACATAATCACG 645
avrXa27               CTCAGCCAACACCCGGCAGCGTTAGGGACCGTCGCTGTCAAGTATCAGCACATAATCACG 645
pxo99a_5a             CTCAGCCAACACCCGGCAGCGTTAGGGACCGTTGCTGTCACGTATCAGGACATAATCAGG 630
pxo86_tal5a           CTCAGCCAACACCCGGCAGCGTTAGGGACCGTTGCTGTCACGTATCAGGACATAATCAGG 630
maff311018_tal3a      CTCAGCCAACACCCGGCAGCGTTAGGGACCGTCGCTGTCAAGTATCAGCACATAATCACG 630
cfbp7342_tal8         CTCAGCCAACACCCGGCAGCGTTAGGGACCGTCGCTGTCACGTATCAGCACATAATCACG 660
                      ******************************** ******* ******* **** ***

avrBs3                GCGTTGCCAGAGGCGACACACGAAGCGATCGTTGGCGTCGGCAAACAGTGGTCCGGCGCA 705
bls256_tal2g          GCGTTGCCAGAGGCGACACACGAAGACATCGTTGGCGTCGGCAAACAGTGGTCCGGCGCA 705
avrXa27               GCGTTGCCAGAGGCGACACACGAAGACATCGTTGGCGTCGGCAAACAGTGGTCCGGCGCA 705
pxo99a_5a             GCGTTGCCAGAGGCGACACACGAAGACATCGTTGGCGTCGGCAAACAGTGGTCCGGCGCA 690
pxo86_tal5a           GCGTTGCCAGAGGCGACACACGAAGACATCGTTGGCGTCGGCAAACAGTGGTCCGGCGCA 690
maff311018_tal3a      GCGTTGCCAGAGGCGACACACGAAGACATCGTTGGCGTCGGCAAACAGTGGTCCGGCGCA 690
cfbp7342_tal8         GCGTTGCCAGAGGCGACACGCGAAGACATCGTTGGCGTCGGCAAACAGTGGTCCGGCGCA 720
                      ******************* *****  *********************************

avrBs3                CGCGCTCTGGAGGCCTTGCTCACGGTGGCGGGAGAGTTGAGAGGTCCACCGTTACAGTTG 765
bls256_tal2g          CGCGCCCTGGAGGCCTTGCTCGCGGATGCGGGGGAGTTGAGAGGTCCGCCGTTACAGTTG 765
avrXa27               CGCGCCCTGGAGGCCTTGCTCACGAAGGCGGGGGAGTTGAGAGGTCCGCCGTTACAGTTG 765
pxo99a_5a             CGCGCCCTGGAGGCCTTGCTCACGGAGGCGGGGGAGTTGAGAGGTCCGCCGTTACAGTTG 750
pxo86_tal5a           CGCGCCCTGGAGGCCTTGCTCACGGAGGCGGGGGAGTTGAGAGGTCCGCCGTTACAGTTG 750
maff311018_tal3a      CGCGCCCTGGAGGCCTTGCTCACGAAGGCGGGGGAGTTGAGAGGTCCGCCGTTACAGTTG 750
cfbp7342_tal8         CGCGCCCTGGAGGCCTTGCTCACGAAGGCGGGGGAGTTGAGAGGTCCACCGTTACAGTTG 780
                      ***** *************** **   ***** ************** ************

avrBs3                GACACAGGCCAACTTCTCAAGATTGCAAAACGTGGCGGCGTGACCGCAGTGGAGGCAGTG 825
bls256_tal2g          GACACAGGCCAACTTCTCAAGATTGCAAAACGTGGCGGCGTGACCGCAGTGGAGGCAGTG 825
avrXa27               GACACAGGCCAACTTCTCAAGATTGCAAAACGTGGCGGCGTGACCGCAGTGGAGGCAGTG 825
pxo99a_5a             GACACAGGCCAACTTCTCAAGATTGCAAAACGTGGCGGCGTGACCGCAGTGGAGGCAGTG 810
pxo86_tal5a           GACACAGGCCAACTTCTCAAGATTGCAAAACGTGGCGGCGTGACCGCAGTGGAGGCAGTG 810
maff311018_tal3a      GACACAGGCCAACTTCTCAAGATTGCAAAACGTGGCGGCGTGACCGCAGTGGAGGCAGTG 810
cfbp7342_tal8         GACACAGGCCAACTTGTGAAGATTGCAAAACGTGGCGGCGTGACCGCAGTGGAGGCAGTG 840
                      *************** * ******************************************

avrBs3                CATGCATGGCGCAATGCACTGACGGGTGCCCCCCTGAAC 864
bls256_tal2g          CATGCATCGCGCAATGCACTGACGGGTGCCCCCCTGAAC 864
avrXa27               CATGCATCGCGCAATGCACTGACGGGTGCCCCCCTGAAC 864
pxo99a_5a             CATGCATGGCGCAATGCACTGACGGGTGCCCCCCTGAAC 849
pxo86_tal5a           CATGCATGGCGCAATGCACTGACGGGTGCCCCCCTGAAC 849
maff311018_tal3a      CATGCATGGCGCAATGCACTGACGGGTGCCCCCCTGAAC 849
cfbp7342_tal8         CATGCATGGCGCAATGCACTGACGGGTGCCCCCCTGAAC 879
                      ******* *******************************
```
