# Supplementary material for: Single molecule real-time sequencing of Xanthomonas oryzae genomes reveals a dynamic structure and complex TAL (transcription activator-like) effector gene relationships
Source: Microb Genom. 2015 Oct 30;1(4):e000032. doi: 10.1099/mgen.0.000032 (PMC4853030; doi:10.1099/mgen.0.000032)
Supplement: Supplementary file 7 — Supplementary Data [file mgen-01-32-s007.pdf]

Supplementary Material
for

SMRT SEQUENCING OF *XANTHOMONAS ORYZAE* GENOMES REVEALS A DYNAMIC STRUCTURE AND COMPLEX TAL EFFECTOR GENE RELATIONSHIPS

Nicholas J. Booher[1], Sara C. D. Carpenter[1], Robert P. Sebra[2], Li Wang[1], Steven L. Salzberg[3], Jan E. Leach[4], and Adam J. Bogdanove[1]*
Address: [1] Plant Pathology and Plant-Microbe Biology Section, School of Integrative Plant Science, Cornell University, Ithaca, NY 14853 USA; [2] Icahn Institute for Genomics and Multiscale Biology and Department of Genetics & Genomic Sciences, Icahn School of Medicine at Mount Sinai, New York, NY 10029 USA; [3] Departments of Biomedical Engineering, Computer Science, and Biostatistics and Center for Computational Biology, Johns Hopkins University, Baltimore, MD 21205 USA; [4] Bioagricultural Sciences and Pest Management, Colorado State University, Ft. Collins, CO 80523 USA
***Corresponding author: ajb7@cornell.edu**

File S7. Alignment of *tal* gene 3' ends related to the 129 bp 3' end variant. Sequences of the 3' ends of the archetypal *X. euvesicatoria avrBs3* gene, the BLS256 *tal2g* gene (representing Xoc *tal* genes), and *avrXa27* (representing Xoo *tal* genes) are shown for comparison. The stop codon of each gene is highlighted. Genbank accessions for sequences included in the alignment are given in the legend to File S6, plus AY377126.2 for *arp3*, KF460460.1 for RS105, and KF447887.1 for JSB2-24.

```
avrBs3              AGCATTGTTGCCCAGTTATCTCGCCCTGATCCGGCGTTGGCCGCGTTGACCAACGACCAC 60
bls256_tal2g        AGCATTGTTGCCCAGTTATCTCGCCCTGATCCGGCGTTGGCCGCGTTGACCAACGACCAC 60
avrXa27             AGCATTGTTGCCCAGTTATCTCGCCCTGATCCGGCGTTGGCCGCGTTGACCAACGACCAC 60
arp3                AGCATTGTTGCCCAGTTATCTCGCCCTGATCCGGCGTTGGCCGCGTTGACCAACGACCAC 60
pxo99a_tal3a        AGCATTGTTGCCCAGTTATCTCGCCCTGATCCGGCGTTGGCCGCGTTGACCAACGACCAC 60
pxo86_tal3          AGCATTGTTGCCCAGTTATCTCGCCCTGATCCGGCGTTGGCCGCGTTGACCAACGACCAC 60
bls256_tal2h        AGCATTGTTGCCCAGTTATCTCGCCGTGATCCGGCGTTGGCCGCGTTGACCAAGGACCAA 60
rs105_tal           AGCATTGTTGCCCAGTTATCTCGCCGTGATCCGGCGTTGGCCGCGTTGACCAAGGACCAA 60
jsb2-24_tal         AGCATTGTTGCCCAGTTATCTCGCCGTGATCCGGCGTTGGCCGCGTTGACCAAGGACCAA 60
cfpb7342_tal11g     AGCATTGTTGCCCAGTTATCTCGCCGTGATCCGGCGTTGGCCGCGTTGACCAACGACCAA 60
pxo86_tal6          AGCATTGTTGCCCAGTTATCTCGCCGTGATCCGGCGTTGGCCGCGTTGACCAACGACCAA 60
pxo99a_tal3b        AGCATTGTTGCCCAGTTATCTCGCCGTGATCCGGCGTTGGCCGCGTTGACCAACGACCAA 60
maff311018_tal5     AGCATTGTTGCCCAGTTATCTCGCCGTGATCCGGCGTTGGCCGCGTTGACCAACGACCAA 60
                    ************************* *************************** ***** 

avrBs3              CTCGTCGCCTTGGCCTGCCTCGGCGGACGTCCTGCGCTGGATGCAGTGAAAAAGGGATTG 120
bls256_tal2g        CTCGTCGCCTTGGCCTGCCTCGGCGGGCGTCCTGCCCTGGATGCAGTGAAAAAGGGATTG 120
avrXa27             CTCGTCGCCTTGGCCTGCCTCGGCGGACGTCCTGCCCTGGATGCAGTGAAAAAGGGATTG 120
arp3                CTCGTCGCCTTGGCCTGCCTCGGCGGACGTCCTGCCCTGGATGCAGTGAAAAAGGGATTG 120
pxo99a_tal3a        CTCGTCGCCTTGGCCTGCCTCGGCGGACGTCCTGCCCTGGATGCAGTGAAAAAGGGATTG 120
pxo86_tal3          CTCGTCGCCTTGGCCTGCCTCGGCGGACGTCCTGCCCTGGATGCAGTGAAAAAGGGATTG 120
bls256_tal2h        CTCGTCGCCTTGGCCTGCTTCGGCGGACGTCCTGCC------------------------ 96
rs105_tal           CTCGTCGCCTTGGCCTGCTTCGGCGGACGTCCTGCC------------------------ 96
jsb2-24_tal         CTCGTCGCCTTGGCCTGCTTCGGCGGACGTCCTGCC------------------------ 96
cfpb7342_tal11g     CTCGTCGCCTTGGCCTGCCTCGGCGGACGTCCTGCC------------------------ 96
pxo86_tal6          CTCGTCGCCTTGGCCTGCCTCGGCGGACGTCCTGCC------------------------ 96
pxo99a_tal3b        CTCGTCGCCTTGGCCTGCCTCGGCGGACGTCCTGCC------------------------ 96
maff311018_tal5     CTCGTCGCCTTGGCCTGCCTCGGCGGACGTCCTGCC------------------------ 96
                    ***************** ******* ********

avrBs3              CCGCACGCGCCGGCCTTGATCAAAAGAACCAATCGCCGTATTCCCGAACGCACATCCCAT 180
```

**bls256_tal2g**	CCGCACGCGCCGGAATTCATCAGAAGAGTCAATCGCCGTATTGCCGAACGCACGTCCCAT	180
**avrXa27**	CCGCACGCGCCGGAATTGATCAGAAGAATCAATCGCCGTATTCCCGAACGCACGTCCCAT	180
arp3	CCGCACGCGCCGGAATTGATCAGAAGAGTCAATAGCCGTATTGGCGAACGCACGTCCCAT	180
pxo99a_tal3a	CCGCACGCGCCGGAATTGATCAGAAGAGTCAATAGCCGTATTGGCGAACGCACGTCCCAT	180
pxo86_tal3	CCGCACGCGCCGGAATTGATCAGAAGAGTCAATAGCCGTATTGGCGAACGCACGTCCCAT	180
bls256_tal2h	--	
rs105_tal	--	
jsb2-24_tal	--	
cfpb7342_tal11g	--	
pxo86_tal6	--	
pxo99a_tal3b	--	
maff311018_tal5	--	

**avrBs3**	CGCGTTGCCGACCACGCGCAAGTGGTTCGCGTGCTGGGTTTTTTCCAGTGCCACTCCCAC	240
**bls256_tal2g**	CGCGTTGCCGACTACGCGCAAGTGGTTCGCGTGCTGGAGTTTTTCCAGTGCCACTCCCAC	240
**avrXa27**	CGCGTTGCCGACCTCGCGCACGTGGTGCGCGTGCTTGGTTTTTTCCAGAGCCACTCCCAC	240
arp3	CGCGTTGCCGACCTCGCGCACGTGGTGCGCGTGCTTGGTTTTTTCCAGAGCCACTCCCAC	240
pxo99a_tal3a	CGCGTTGCCGACCTCGCGCACGTGGTGCGCGTGCTTGGTTTTTTCCAGAGCCACTCCCAC	240
pxo86_tal3	CGCGTTGCCGACCTCGCGCACGTGGTGCGCGTGCTTGGTTTTTTCCAGAGCCACTCCCAC	240
bls256_tal2h	--	
rs105_tal	--	
jsb2-24_tal	--	
cfpb7342_tal11g	--	
pxo86_tal6	--	
pxo99a_tal3b	--	
maff311018_tal5	--	

**avrBs3**	CCAGCGCAAGCATTTGATGACGCCATGACGCAGTTCGGGATGAGCAGGCACGGGTTGTTA	300
**bls256_tal2g**	CCAGCGCACGCATTTGATGAGGCCATGACGCAGTTCGGGATGAGCAGGCAAGGGTTGGTA	300
**avrXa27**	CCAGCGCAAGCATTCGATGACGCCATGACGCAGTTCGGGATGAGCAGGCACGGGTTGGTA	300
arp3	CCAGCGCAAGCATTCGATGACGCCATGACGCAGTTCGGGATGAGCAGGCACGGGTTGGTA	300
pxo99a_tal3a	CCAGCGCAAGCATTCGATGACGCCATGACGCAGTTCGGGATGAGCAGGCACGGGTTGGTA	300
pxo86_tal3	CCAGCGCAAGCATTCGATGACGCCATGACGCAGTTCGGGATGAGCAGGCACGGGTTGGTA	300
bls256_tal2h	--	
rs105_tal	--	
jsb2-24_tal	--	
cfpb7342_tal11g	--	
pxo86_tal6	--	
pxo99a_tal3b	--	
maff311018_tal5	--	

**avrBs3**	CAGCTCTTTCGCAGAGTGGGCGTCACCGAACTCGAAGCCCGCAGTGGAACGCTCCCCCCA	360
**bls256_tal2g**	CAGCTCTTTCGCAGAGTGGGCGTCACCGAACTCGAAGCCCGCTGCGGAACGCTCCCCCCA	360
**avrXa27**	CAGCTCTTTCGCAGAGTGGGCGTCACCGAATTCGAAGCCCGCTGCGGAACGCTCCCCCCA	360
arp3	CAGCTCTTTCGCAGAGTGGGCGTCACCGAATTCGAAGCCCGCTGCGGAACTATCCCCCCA	360
pxo99a_tal3a	CAGCTCTTTCGCAGAGTGGGCGTCACCGAATTCGAAGCCCGCTGCGGAACTATCCCCCCA	360
pxo86_tal3	CAGCTCTTTCGCAGAGTGGGCGTCACCGAATTCGAAGCCCGCTGCGGAACTATCCCCCCA	360
bls256_tal2h	--	
rs105_tal	--	
jsb2-24_tal	--	
cfpb7342_tal11g	--	
pxo86_tal6	--	
pxo99a_tal3b	--	
maff311018_tal5	--	

**avrBs3**	GCCTCGCAGCGTTGGGACCGTATCCTCCAGGCATCAGGGATGAAAAGGGCCAAACCGTCC	420
**bls256_tal2g**	GCCTCGCAGCGTTGGGACCGTATCCTCCAGGCATCAGGGATGAAAAGGGGCAAACCGTCC	420
**avrXa27**	GCCTCGCAGCGTTGGGACCGTATCCTCCAGGCATCAGGGATGAAAAGGGCCAAACCGTCC	420
arp3	GCCTCGCAGCGTTGGGACCGTATCCTCCAGGCATCAGGGACGAAAAGGGCCAAACCGTCC	420
pxo99a_tal3a	GCCTCGCAGCGTTGGGACCGTATCCTCCAGGCATCAGGGACGAAAAGGGCCAAACCGTCC	420
pxo86_tal3	GCCTCGCAGCGTTGGGACCGTATCCTCCAGGCATCAGGGACGAAAAGGGCCAAACCGTCC	420
bls256_tal2h	--	
rs105_tal	--	
jsb2-24_tal	--	
cfpb7342_tal11g	--	
pxo86_tal6	--	
pxo99a_tal3b	--	
maff311018_tal5	--	

**avrBs3**	CCTACTTCAACTCAAACGCCGGATCAGGCGTCTTTGCATGCATTCGCCGATTCGCTGGAG	480
**bls256_tal2g**	CCTACTTCAGCTCAAACCCCGGATCAGGCGTCTTTGCATGGATTCGCCGATTCGCTGGAG	480
**avrXa27**	CCTACTTCAGCTCAAACGCCGGATCAGGCGTCTTTGCATGCATTCGCCGATTCGCTGGAG	480
arp3	CCTACTTCAGCTCAGACGCCGGATCAGGCGTCTTTGCATGCATTCCCCGACTCGCTGGAG	480
pxo99a_tal3a	CCTACTTCAGCTCAGACGCCGGATCAGGCGTCTTTGCATGCATTCCCCGACTCGCTGGAG	480
pxo86_tal3	CCTACTTCAGCTCAGACGCCGGATCAGGCGTCTTTGCATGCATTCCCCGACTCGCTGGAG	480
bls256_tal2h	--	
rs105_tal	--	
jsb2-24_tal	--	
cfpb7342_tal11g	--	
pxo86_tal6	--	
pxo99a_tal3b	--	
maff311018_tal5	--	

```
avrBs3             CGTGACCTTGATGCGCCTAGCCCAATGCACGAGGGAGATCAGACGCGGGCAAGCAGCCGT 540
bls256_tal2g       CGTGACCTTGATGCGCCCAGCCCAATGCACGAGGGAGATCAGACGCGGGCAAGCAGCCGT 540
avrXa27            CGTGACCTTGATGCGCCCAGCCCAATGCACGAGGGAGATCAGACGCGGGCAAGCAGCCGT 540
arp3               CGTGACCTTGATGCGCCCAGCCCAATGCACGAGGGAGATCAGACGCGGGCAAGCAGACGT 540
pxo99a_tal3a       CGTGACCTTGATGCGCCCAGCCCAATGCACGAGGGAGATCAGACGCGGGCAAGCAGACGT 540
pxo86_tal3         CGTGACCTTGATGCGCCCAGCCCAATGCACGAGGGAGATCAGACGCGGGCAAGCAGACGT 540
bls256_tal2h       ------------------------------------------------------------
rs105_tal          ------------------------------------------------------------
jsb2-24_tal        ------------------------------------------------------------
cfpb7342_tal11g    ------------------------------------------------------------
pxo86_tal6         ------------------------------------------------------------
pxo99a_tal3b       ------------------------------------------------------------
maff311018_tal5    ------------------------------------------------------------

avrBs3             AAACGGTCCCGATCGGATCGTGCTGTCACCGGTCCCTCCGCACAGCAATCGTTCGAGGTG 600
bls256_tal2g       AAACGGTCCCGATCGGATCATGCTGTCACCGGCCCCTCCGCACAGCAGGCTGTCGAGGTG 600
avrXa27            AAACGGTCCCGATCGGATCGTGCTGTCACCGGCCCCTCCACACAGCAATCTTTCGAGGTG 600
arp3               AAACGGTCCCGATCGGATCGTGCTGTCACCGACCCCTCCGCACAGCAATCTTTCGAGGTG 600
pxo99a_tal3a       AAACGGTCCTGATCGGATCGTGCTGTCACCGACCCCTCCGCACAGCAATCTTTCGAGGTG 600
pxo86_tal3         AAACGGTCCTGATCGGATCGTGCTGTCACCGACCCCTCCGCACAGCAATCTTTCGAGGTG 600
bls256_tal2h       ------------------------------------------------------------
rs105_tal          ------------------------------------------------------------
jsb2-24_tal        ------------------------------------------------------------
cfpb7342_tal11g    ------------------------------------------------------------
pxo86_tal6         ------------------------------------------------------------
pxo99a_tal3b       ------------------------------------------------------------
maff311018_tal5    ------------------------------------------------------------

avrBs3             CGCGTTCCCGAACAGCGCGATGCGCTGCATTTGCCCCTCAGTTGGAGGGTAAAACGCCCG 660
bls256_tal2g       CGCGTTCCCGAACAGCGCGATGCGCTGCATTTGCCCCTCAGCTGGAGTGTAAAACGCCCG 660
avrXa27            CGCGTTCCCGAACAGCGCGATGCGCTGCATTTGCCCCTCAGCTGGAGGGTAAAACGCCCG 660
arp3               CGCGTTCCCGAACAGCACGATGCGCTGCATTTGCCCCTCAGCTGGAGGGTAAAACGCCCG 660
pxo99a_tal3a       CGCGTTCCCGAACAGCACGATGCGCTGCATTTGCCCCTCAGCTGGAGGGTAAAACGCCCG 660
pxo86_tal3         CGCGTTCCCGAACAGCACGATGCGCTGCATTTGCCCCTCAGCTGGAGGGTAAAACGCCCG 660
bls256_tal2h       ------------------------------------------------------------
rs105_tal          ------------------------------------------------------------
jsb2-24_tal        ------------------------------------------------------------
cfpb7342_tal11g    ------------------------------------------------------------
pxo86_tal6         ------------------------------------------------------------
pxo99a_tal3b       ------------------------------------------------------------
maff311018_tal5    ------------------------------------------------------------

avrBs3             CGTACCAGTATCGGGGGCGGCCTCCCGGATCCTGGTACGCCCACGGCTGCCGACCTGGCA 720
bls256_tal2g       CGTACCAGGATCGGGGGCGGCCTCCCGGATCCTGGTACGCCCATGGCTGCCGACCTGGCA 720
avrXa27            CGTACCAGGATCGGGGGCGGCCTCCCGGATCCTGGTACGCCCATCGCTGCCGACCTGGCA 720
arp3               CGTACCAGGATCGGGGGCGGCCTCCCGGATCCTGGTACGCCCATGGTTGCCGACCTGGCA 720
pxo99a_tal3a       CGTACCAGGATCGGGGGCGGCCTCCCGGATCCTGGTACGCCCATGGTTGCCGACCTGGCA 720
pxo86_tal3         CGTACCAGGATCGGGGGCGGCCTCCCGGATCCTGGTACGCCCATGGTTGCCGACCTGGCA 720
bls256_tal2h       ------------------------------------------------------------
rs105_tal          ------------------------------------------------------------
jsb2-24_tal        ------------------------------------------------------------
cfpb7342_tal11g    ------------------------------------------------------------
pxo86_tal6         ------------------------------------------------------------
pxo99a_tal3b       ------------------------------------------------------------
maff311018_tal5    ------------------------------------------------------------
```

```
avrBs3               GCGTCCAGCACCGTGATGCGGGAACAAGATGAGGACCCCTTCGCAGGGGCAGCGGATGAT 780
bls256_tal2g         GCGTCCAGCACCGTGATGTGGGAACAAGATGCGGCCCCCTTCGCAGGGGCAGCGGATGAT 780
avrXa27              GCGTCCAGCACCGTGATGTGGGAACAAGATGCGGCCCCCTTCGCAGGGGCAGCGGATGAT 780
arp3                 GCGTCCAGCACCGTGATGTGGGAACAAGATGCGGCCCCCTTCGCAGGGGCAGCGGATGAT 780
pxo99a_tal3a         GCGTCCAGCACCGTGATGTGGGAACAAGATGCGGCCCCCTTCGCAGGGGCAGCGGATGAT 780
pxo86_tal3           GCGTCCAGCACCGTGATGTGGGAACAAGATGCGGCCCCCTTCGCAGGGGCAGCGGATGAT 780
bls256_tal2h         ------------------------------------------------------------
rs105_tal            ------------------------------------------------------------
jsb2-24_tal          ------------------------------------------------------------
cfpb7342_tal11g      ------------------------------------------------------------
pxo86_tal6           ------------------------------------------------------------
pxo99a_tal3b         ------------------------------------------------------------
maff311018_tal5      ------------------------------------------------------------

avrBs3               TTCCCGGCATTCAACGAAGAGGAGCTCGCATGGTTGATGGAGCTATTGCCTCAGTGAAGT 840
bls256_tal2g         TTCCCGGCATTCAACGAAGAGGAACTCGCATGGTTGATGGAGCTATTGCCTCAGTGAGGC 840
avrXa27              TTCCCGGCATTCAACGAAGAGGAGCTCGCATGGTTGATGGAGCTATTGCCTCAGTCAGGC 840
arp3                 TTCCCGGCATTCAACGAAGAGGAGCTCGCATGGTTGATGGAGCTATTGCCTCAGTCAGGC 840
pxo99a_tal3a         TTCCCGGCATTCAACGAAGAGGAGCTCGCATGGTTGATGGAGCTATTGCCTCAGTGAGGC 840
pxo86_tal3           TTCCCGGCATTCAACGAAGAGGAGCTCGCATGGTTGATGGAGCTATTGCCTCAGTGAGGC 840
bls256_tal2h         ----CCGCATTCAAGGAAGAGGAAATCGCATGATTGATGGAGCTATTGCCTCAGTGAGGC 152
rs105_tal            ----CCGCATTCAAGGAAGAGGAAATCGCATGATTGATGGAGCTATTGCCTCAGTGAGGC 152
jsb2-24_tal          ----CCGCATTCAAGGAAGAGGAAATCGCATGATTGATGGAGCTATTGCCTCAGTGAGGC 152
cfpb7342_tal11g      ----CCGCATTCAAGGAAGAGGAAATCGCATGATTGATGGAGCTATTGCCTCAGTGAGGC 152
pxo86_tal6           ----CCGCATTCAAGGAAGAGGAAATCGCATGATTGATGGAGCTATTGCCTCAGTGAGGC 152
pxo99a_tal3b         ----CCGCATTCAAGGAAGAGGAAATCGCATGATTGATGGAGCTATTGCCTCAGTGAGGC 152
maff311018_tal5      ----CCGCATTCAAGGAAGAGGAAATCGCATGATTGATGGAGCTATTGCCTCAGTGAGGC 152
                         * ******** ********  ******* ********************** * *

avrBs3               GAGGCTCAGTCGGTGACTACC      861
bls256_tal2g         TCGACGTCCTCCCCTCCTGAG      861
avrXa27              TCAGTCGGAGGGACGATCTGA      861
arp3                 TCAGTCGGAGGGACGATCTGA      861
pxo99a_tal3a         TCAGTCGGGGGACTATCTGAG      861
pxo86_tal3           TCAGTCGGGGGACTATCTGAG      861
bls256_tal2h         TCAGTCGGGGGACTATCTGAG      173
rs105_tal            TCAGTCGGGGGACTATCTGAG      173
jsb2-24_tal          TCAGTCGGGGGACTATCTGAG      173
cfpb7342_tal11g      TCAGTCGGGGGACTATCTGAG      173
pxo86_tal6           TCAGTCGGGGGACTATCTGAG      173
pxo99a_tal3b         TCAGTCGGGGGACTATCTGAG      173
maff311018_tal5      TCAGTCGGGGGACTATCTGAG      173
```
