# Supplementary material for: Single molecule real-time sequencing of Xanthomonas oryzae genomes reveals a dynamic structure and complex TAL (transcription activator-like) effector gene relationships
Source: Microb Genom. 2015 Oct 30;1(4):e000032. doi: 10.1099/mgen.0.000032 (PMC4853030; doi:10.1099/mgen.0.000032)
Supplement: Supplementary file 8 — Supplementary Data [file mgen-01-32-s008.pdf]

# Supplementary Material
# for

# SMRT SEQUENCING OF *XANTHOMONAS ORYZAE* GENOMES REVEALS A DYNAMIC STRUCTURE AND COMPLEX TAL EFFECTOR GENE RELATIONSHIPS

**Nicholas J. Booher[1], Sara C. D. Carpenter[1], Robert P. Sebra[2], Li Wang[1], Steven L. Salzberg[3], Jan E. Leach[4], and Adam J. Bogdanove[1]***
**Address: [1] Plant Pathology and Plant-Microbe Biology Section, School of Integrative Plant Science, Cornell University, Ithaca, NY 14853 USA; [2] Icahn Institute for Genomics and Multiscale Biology and Department of Genetics & Genomic Sciences, Icahn School of Medicine at Mount Sinai, New York, NY 10029 USA; [3] Departments of Biomedical Engineering, Computer Science, and Biostatistics and Center for Computational Biology, Johns Hopkins University, Baltimore, MD 21205 USA; [4] Bioagricultural Sciences and Pest Management, Colorado State University, Ft. Collins, CO 80523 USA**
***Corresponding author: ajb7@cornell.edu**

**File S8. Alignment of *tal* gene 5' ends with large deletions that also have 3' ends related to the 129 bp 3' variant.** Sequences of the 5' ends of the archetypal *X. euvesicatoria avrBs3* gene, the BLS256 *tal2g* gene (representing Xoc *tal* genes), and *avrXa27* (representing Xoo *tal* genes) are shown for comparison. Genbank accessions for sequences included in the alignment are given in the legend to File S6.

```
avrBs3            ATGGATCCCATTCGTTCGCGCACACCAAGTCCTGCCCGCGAGCTTCTGCCCGGACCCCAA 60
bls256_tal2g      ATGGATCCCATTCGTCCGCGCAGGCCAAGTCCTGCCCGCGAGCCTCTGCCCGGACCCCAA 60
avrXa27           ATGGATCCCATTCGTTCGCGCACGCCAAGTCCTGCCCGCGAGCTTCTGCCCGGACCCCAA 60
arp3              ATGGATCCCATTCGTTCGCGCACGCCAAGTCCTGCCCGCGAGCCTCTGCCCGGACCCCAA 60
pxo99a_tal3a      ATGGATCCCATTCGTTCGCGCACGCCAAGTCCTGCCCGCGAGCCTCTGCCCGGACCCCAA 60
pxo86_tal3        ATGGATCCCATTCGTTCGCGCACGCCAAGTCCTGCCCGCGAGCCTCTGCCCGGACCCCAA 60
bls256_tal2h      ATGGATCCCATTCGTTCACGCACGCCAAGTCCTGCCCGCGAGCCTCTGCCCGGACCCCAA 60
rs105_tal         ATGGATCCCATTCGTTCACGCACGCCAAGTCCTGCCCGCGAGCCTCTGCCCGGACCCCAA 60
jsb2-24_tal       ATGGATCCCATTCGTTCACGCACGCCAAGTCCTGCCCGCGAGGGTCTGCCCGGACCCCAA 60
pxo86_tal6        ATGGATCCCATTCGTTCGCGCACGCCAAGTCCTGCCCGCGAGCCTCTGCCCGGACCCCAA 60
pxo99a_tal3b      ATGGATCCCATTCGTTCGCGCACGCCAAGTCCTGCCCGCGAGCCTCTGCCCGGACCCCAA 60
maff311018_tal5   ATGGATCCCATTCGTTCGCGCACGCCAAGTCCTGCCCGCGAGCCTCTGCCCGGACCCCAA 60
                  *************** * ****  ******************  ****************

avrBs3            CCCGATGGGGTTCAGCCGACTGCAGATCGTGGGGTGTCTCCGCCTGCCGGCGGCCCCCTG 120
bls256_tal2g      CCGGATGGGGTTCAGCCGACTGCAGATCGGGGGGTGTCTGCGCCTGCTGGCGGCCCTCTG 120
avrXa27           CCGGATAGGGTTCAGCCGACTGCAGATCGGGGGGGGGCTCCGCCTGCTGGCGGCCCCCTG 120
arp3              CCGGATAGGGTTCAGCCGACTGCAGATCGTGGGGTGTCTGCGCCTGCTGGCAGCCCTCTG 120
pxo99a_tal3a      CCGGATAGGGTTCAGCCGACTGCAGATCGTGGGGTGTCTGCGCCTGCTGGCAGCCCTCTG 120
pxo86_tal3        CCGGATAGGGTTCAGCCGACTGCAGATCGTGGGGTGTCTGCGCCTGCTGGCAGCCCTCTG 120
bls256_tal2h      CCGGATAGGGTTCAGCCGACTGCAGATCGTGGGGTGTCTGCGCCTGCTGGCGGCCCCCTG 120
rs105_tal         CCGGATAGGGTTCAGCCGACTGCAGATCGTGGGGTGTCTGCGCCTGCTGGCGGCCCCCTG 120
jsb2-24_tal       CCGGATAGGGTTCAGCCGACTGCAGATCGTGGGGTGTCTGCGCCTGCTGGCAGCCCTCTG 120
pxo86_tal6        CCGGATAGGGTTCAGCCGACTGCAGATCGTGGGGTGTCTGCGCCTGCTGGCAGCCCTCTG 120
pxo99a_tal3b      CCGGATAGGGTTCAGCCGACTGCAGATCGTGGGGTGTCTGCGCCTGCTGGCAGCCCTCTG 120
maff311018_tal5   CCGGATAGGGTTCAGCCGACTGCAGATCGTGGGGTGTCTGCGCCTGCTGGCAGCCCTCTG 120
                  ** *** ********************** **** * ** ******* *** **** ***

avrBs3            GATGGCTTGCCCGCTCGGCGGACGATGTCCCGGACCCGGCTGCCATCTCCCCCTGCCCCC 180
bls256_tal2g      GATGGTTTGCCCGCTCGGCGGACGATGTCCCGGACCCGGCTGCCATCTCCCCCTGCGCCC 180
avrXa27           GATGGCTTGCCCGCTCGGCGGACGATGTCCCGGACCCGGCTGCCATCTCCCCCTGCGCCC 180
arp3              GATGGCTTGCCCGCTCGGCGGACGGTGTCCCGGACCCGGCTGCCATCTCCCCCTGCCCCC 180
```

```
pxo99a_tal3a         GATGGCTTGCCCGCTCGGCGGACGGTGTCCCGGACCCGGCTGCCATCTCCCCCTGCCCCC 180
pxo86_tal3           GATGGCTTGCCCGCTCGGCGGACGGTGTCCCGGACCCGGCTGCCATCTCCCCCTGCCCCC 180
bls256_tal2h         GATGCCTTGCCCGCTCGGCGGACGGTGTCCCGGACCCGGCTGCCATCTCCCCCTGCGCCC 180
rs105_tal            GATGCCTTGCCCGCTCGGCGGACGGTGTCCCGGACCCGGCTGCCATCTCCCCCTGCGCCC 180
jsb2-24_tal          GATGGCTTGCCCGCTCGGCGGACGGTGTCCCGGACCCGGCTGCCATCTCCCCCTGCCCCC 180
pxo86_tal6           GATGGCTTGCCCGCTCGGCGGACGGTGTCCCGGACCCGGCTGCCATCTCCCCCTGCCCCC 180
pxo99a_tal3b         GATGGCTTGCCCGCTCGGCGGACGGTGTCCCGGACCCGGCTGCCATCTCCCCCTGCCCCC 180
maff311018_tal5      GATGGCTTGCCCGCTCGGCGGACGGTGTCCCGGACCCGGCTGCCATCTCCCCCTGCCCCC 180
                     ****  ****************** ******************************* ***

avrBs3               TCACCTGCGTTCTCGGCGGGCAGCTTCAGTGACCTGTTACGTCAGTTCGATCCGTCACTT 240
bls256_tal2g         TCGCCTGCGTTCTCGGCGGGCAGCTTCAGCGATCTGCTCCGTCCGTTCGATCCGTCGCTT 240
avrXa27              TCGCCTGCGTTCTCGGCGGGCAGCTTCAACGATCTGCTCCGTCAGTTCGATCCGTCGCTT 240
arp3                 TTGCCTGCGTTCTCGGCGGGCAGCTCCACCGATCGGCTCCGTCAGTTCGATCCGTCGCTT 240
pxo99a_tal3a         TTGCCTGCGTTCTCGGCGGGCAGCTCCACCGATCGGCTCCGTCCGTTCGATCCGTCGCTT 240
pxo86_tal3           TTGCCTGCGTTCTCGGCGGGCAGCTCCACCGATCGGCTCCGTCCGTTCGATCCGTCGCTT 240
bls256_tal2h         TTGCCTGCGTTCTCGGCGGGCAGCTCCACCGATCGGCTCCGTCCGTTCGATCCGTCGCTT 240
rs105_tal            TTGCCTGCGTTCTCGGCGGGCAGCTCCACCGATCGGCTCCGTCCGTTCGATCCGTCGCTT 240
jsb2-24_tal          TTGCCTGCGTTCTCGGCGGGCAGCTCCACCGATCGGCTCCGTCCGTTCGATCCGTCGCTT 240
pxo86_tal6           TTGCCTGCGTTCTCGGCGGGCAGCTCCACCGATCGGCTCCGTCCGTTCGATCCGTCGCTT 240
pxo99a_tal3b         TTGCCTGCGTTCTCGGCGGGCAGCTCCACCGATCGGCTCCGTCCGTTCGATCCGTCGCTT 240
maff311018_tal5      TTGCCTGCGTTCTCGGCGGGCAGCTCCACCGATCGGCTCCGTCCGTTCGATCCGTCGCTT 240
                     *  ********************** **  ** * * * **** ************ ***

avrBs3               TTTAATACATCGCTTTTTGATTCATTGCCTCCCTTCGGCGCTCACCATACAGAGGCTGCC 300
bls256_tal2g         CTTGATACATCGCTTCTTGATTCGATGCCTGCCGTCGGCACGCCGCATACAGCGGCTGCC 300
avrXa27              CTTGATACATCGCTTCTTGATTCGATGCCTGCCGTCGGCACGCCGCATACAGCGGCTGCC 300
arp3                 CCTGATACATCGCTTTTTGATTCGATGCCTGCCGTCGGCACGCCTCATACAGAGGCTGCC 300
pxo99a_tal3a         CCTGATACATCGCTTTTTGATTCGATGCCTGCCGTCGGCACGCCTCATACAGAGGCTGCC 300
pxo86_tal3           CCTGATACATCGCTTTTTGATTCGATGCCTGCCGTCGGCACGCCTCATACAGAGGCTGCC 300
bls256_tal2h         CCTGATACATCGCTTTTTGATTCGATGCCTGCCGTCGGCACGCCGCATACAGAGGCTGCC 300
rs105_tal            CCTGATACATCGCTTTTTGATTCGATGCCTGCCGTCGGCACGCCGCATACAGAGGCTGCC 300
jsb2-24_tal          CCTGATACATCGCTTTTTGATTCGATGCCTGCCGTCGGCACGCCGCATACAGAGGCTGCC 300
pxo86_tal6           CCTGATACATCGCTTTTTGATTCGATGCCTGCCGTCGGCACGCCTCATACAGAGGCTGCC 300
pxo99a_tal3b         CCTGATACATCGCTTTTTGATTCGATGCCTGCCGTCGGCACGCCTCATACAGAGGCTGCC 300
maff311018_tal5      CCTGATACATCGCTTTTTGATTCGATGCCTGCCGTCGGCACGCCTCATACAGAGGCTGCC 300
                       * *********** *******  ***** ** ***** * *  ******* *******

avrBs3               ACAGGCGAGTGGGATGAGGTGCAATCGGGTCTGCGGGCAGCCGACGCCCCCCCACCCACC 360
bls256_tal2g         CCAGCAGAGTGGGATGAGGCGCAATCGGGTCTGCGTGCAGCCGATGACCCGCCACCCACC 360
avrXa27              CCAGCAGAGTGGGATGAGGTGCAATCGGGTCTGCGTGCAGCCGATGACCCGCCACCCACC 360
arp3                 CCAGCAGAC--------------------------------------------------- 309
pxo99a_tal3a         CCAGCAGAC--------------------------------------------------- 309
pxo86_tal3           CCAGCAGAC--------------------------------------------------- 309
bls256_tal2h         CCAGCAGAC--------------------------------------------------- 309
rs105_tal            CCAGCAGAC--------------------------------------------------- 309
jsb2-24_tal          CCAGCAGAC--------------------------------------------------- 309
pxo86_tal6           CCAGCAGAC--------------------------------------------------- 309
pxo99a_tal3b         CCAGCAGAC--------------------------------------------------- 309
maff311018_tal5      CCAGCAGAC--------------------------------------------------- 309
                      ***  **

avrBs3               ATGCGCGTGGCTGTCACTGCCGCGCGGCCGCCGCGCGCCAAGCCGGCGCCGCGACGACGT 420
bls256_tal2g         GTGCGTGTCGCTGTCACTGCCGCGCGGCCGCCGCGCGCCAAGCCGGCCCCGCGACGGCGT 420
avrXa27              GTGCGTGTCGCTGTCACTGCCGCGCGGCCGCCGCGCGCCAAGCCGGCCCCGCGACGGCGT 420
arp3                 ------------------------------------------------------------
pxo99a_tal3a         ------------------------------------------------------------
pxo86_tal3           ------------------------------------------------------------
bls256_tal2h         ------------------------------------------------------------
rs105_tal            ------------------------------------------------------------
jsb2-24_tal          ------------------------------------------------------------
pxo86_tal6           ------------------------------------------------------------
pxo99a_tal3b         ------------------------------------------------------------
```

```
maff311018_tal5      ------------------------------------------------------------


avrBs3               GCTGCGCAACCCTCCGACGCTTCGCCGGCCGCGCAGGTGGATCTACGCACGCTCGGCTAC 480
bls256_tal2g         GCGGCGCAACCCTCCGACGCTTCGCCGGCCGCGCAGGTGGATCTAAGCACGCTCGGCTAC 480
avrXa27              GCGGCGCAACCCTCCGACGCTTCGCCGGCCGCGCAGGTGGATCTACGCACGCTCGGCTAC 480
arp3                 ------------------ACTTCGCCGGCCGCGCAGGTGGATCTACTCACGCTC------ 345
pxo99a_tal3a         ------------------ACTTCGCCGGCCGCGCAGGTGGATCTACTCACGCTC------ 345
pxo86_tal3           ------------------ACTTCGCCGGCCGCGCAGGTGGATCTACTCACGCTC------ 345
bls256_tal2h         ------------------ACTTCGCCGGCCGCGCAGGTGGATCTACTCACGCTC------ 345
rs105_tal            ------------------ACTTCGCCGGCCGCGCAGGTGGATCTACTCACGCTC------ 345
jsb2-24_tal          ------------------ACTTCGCCGGCCGCGCAGGTGGATCTACTCACGCTC------ 345
pxo86_tal6           ------------------ACTTCGCCGGCCGCGCAGGTGGATCTACTCACGCTC------ 345
pxo99a_tal3b         ------------------ACTTCGCCGGCCGCGCAGGTGGATCTACTCACGCTC------ 345
maff311018_tal5      ------------------ACTTCGCCGGCCGCGCAGGTGGATCTACTCACGCTC------ 345
                                        **************************  *******
```

```
avrBs3             AGCCAGCAGCAACAGGAGAAGATCAAACCGAAGGTTCGTTCGACAGTGGCGCAGCACCAC 540
bls256_tal2g       AGTCAGCAGCAGCAAGAGAAGATCAAACCGAATGTGCGTTCGACAGTGGCGCAGCACCAC 540
avrXa27            AGTCAGCAGCAGCAAGAGAAGATCAAATCGAAGGTGCGTTCGACAGTGGCGCAGCACCAC 540
arp3               ---------------------------------------GCGACAGTGGCGCAGCACCAC 366
pxo99a_tal3a       ---------------------------------------GCGACAGTGGCGCAGCACCAC 366
pxo86_tal3         ---------------------------------------GCGACAGTGGCGCAGCACCAC 366
bls256_tal2h       ---------------------------------------GCGACAGTGGCGCAGCACCAC 366
rs105_tal          ---------------------------------------GCGACAGTGGCGCAGCACCAC 366
jsb2-24_tal        ---------------------------------------GCGACAGTGGCGCAGCACCAC 366
pxo86_tal6         ---------------------------------------GCGACAGTGGCGCAGCACCAC 366
pxo99a_tal3b       ---------------------------------------GCGACAGTGGCGCAGCACCAC 366
maff311018_tal5    ---------------------------------------GCGACAGTGGCGCAGCACCAC 366
                                                           ********************

avrBs3             GAGGCACTGGTCGGCCATGGGTTTACACACGCGCACATCGTTGCGCTCAGCCAACACCCG 600
bls256_tal2g       GAGGCACTGGTGGGCCATGGGTTTACACACGCGCACATCGTTGCGCTCAGCCAACACCCG 600
avrXa27            GAGGCACTGGTGGGCCATGGGTTTACACACGCGCACATCGTTGCGCTCAGCCAACACCCG 600
arp3               GAGGCACTGGTGGGCCATGGGTTTACACACGCGCACATCGTTGCGCTCAGCCAACACCCG 426
pxo99a_tal3a       GAGGCACTGGTGGGCCATGGGTTTACACACGCGCACATCGTTGCGCTCAGCCAACACCCG 426
pxo86_tal3         GAGGCACTGGTGGGCCATGGGTTTACACACGCGCACATCGTTGCGCTCAGCCAACACCCG 426
bls256_tal2h       GAGGCACTGGTGGGCCATGGGTTTACACACGCGCACATCGTTGCGCTCAGCCAACACCCG 426
rs105_tal          GAGGCACTGGTGGGCCATGGGTTTACACACGCGCACATCGTTGCGCTCAGCCAACACCCG 426
jsb2-24_tal        GAGGCACTGGTGGGCCATGGGTTTACACACGCGCACATCGTTGCGCTCAGCCAACACCCG 426
pxo86_tal6         GAGGCACTGGTGGGCCATGGGTTTACACACGCGCACATCGTTGCGCTCAGCCAACACCCG 426
pxo99a_tal3b       GAGGCACTGGTGGGCCATGGGTTTACACACGCGCACATCGTTGCGCTCAGCCAACACCCG 426
maff311018_tal5    GAGGCACTGGTGGGCCATGGGTTTACACACGCGCACATCGTTGCGCTCAGCCAACACCCG 426
                   *********** ************************************************

avrBs3             GCAGCGTTAGGGACCGTCGCTGTCAAGTATCAGGACATGATCGCAGCGTTGCCAGAGGCG 660
bls256_tal2g       GCAGCGTTAGGGACCGTTGCTGTCACGTATCAGCACATAATCACGGCGTTGCCAGAGGCG 660
avrXa27            GCAGCGTTAGGGACCGTCGCTGTCAAGTATCAGCACATAATCACGGCGTTGCCAGAGGCG 660
arp3               GCAGCGTTAGGGACCGTTGCTGTCACGTATCAAGACATAATCACGGCGTTGCCAGAGGCG 486
pxo99a_tal3a       GCAGCGTTAGGGACCGTTGCTGTCACGTATCAAGACATAATCACGGCGTTGCCAGAGGCG 486
pxo86_tal3         GCAGCGTTAGGGACCGTTGCTGTCACGTATCAAGACATAATCACGGCGTTGCCAGAGGCG 486
bls256_tal2h       GCAGCGTTAGGGACCGTTGCTGTCATGTATCAGCACATAATCACGGCGTTGCCAGAGGCG 486
rs105_tal          GCAGCGTTAGGGACCGTTGCTGTCATGTATCAGCACATAATCACGGCGTTGCCAGAGGCG 486
jsb2-24_tal        GCAGCGTTAGGGACCGTTGCTGTCATGTATCAAGACATAATCACGGCGTTGCCAGAGGCG 486
pxo86_tal6         GCAGCGTTAGGGACCGTTGCTGTCACGTATCAAGACATAATCACGGCGTTGCCAGAGGCG 486
pxo99a_tal3b       GCAGCGTTAGGGACCGTTGCTGTCACGTATCAAGACATAATCACGGCGTTGCCAGAGGCG 486
maff311018_tal5    GCAGCGTTAGGGACCGTTGCTGTCACGTATCAAGACATAATCACGGCGTTGCCAGAGGCG 486
                   ***************** ******* ******  **** *** * ***************

avrBs3             ACACACGAAGCGATCGTTGGCGTCGGCAAACAGTGGTCCGGCGCACGCGCTCTGGAGGCC 720
bls256_tal2g       ACACACGAAGACATCGTTGGCGTCGGCAAACAGTGGTCCGGCGCACGCGCCCTGGAGGCC 720
avrXa27            ACACACGAAGACATCGTTGGCGTCGGCAAACAGTGGTCCGGCGCACGCGCCCTGGAGGCC 720
arp3               ACACACGAAGACATCGTTGGCGTCGGCAAACAGTTGTCCGGCGCACGCGCCCTGGAGGCC 546
pxo99a_tal3a       ACACACGAAGACATCGTTGGCGTCGGCAAACAGTTGTCCGGCGCACGCGCCCTGGAGGCC 546
pxo86_tal3         ACACACGAAGACATCGTTGGCGTCGGCAAACAGTTGTCCGGCGCACGCGCCCTGGAGGCC 546
bls256_tal2h       ACACACGAAGACATCGTTGGCGTCGGCAAACAGTTGTCCGACGCACGCGCCCTGGAGGCC 546
rs105_tal          ACACACGAAGACATCGTTGGCGTCGGCAAACAGTTGTCCGACGCACGCGCCCTGGAGGCC 546
jsb2-24_tal        ACACACGAAGACATCGTTGGCGTCGGCAAACAGTTGTCCGGCGCACGCGCCCTGGAGGCC 546
pxo86_tal6         ACACACGAAGACATCGTTGGCGTCGGCAAACAGTTGTCCGGCGCACGCGCCCTGGAGGCC 546
pxo99a_tal3b       ACACACGAAGACATCGTTGGCGTCGGCAAACAGTTGTCCGGCGCACGCGCCCTGGAGGCC 546
maff311018_tal5    ACACACGAAGACATCGTTGGCGTCGGCAAACAGTTGTCCGGCGCACGCGCCCTGGAGGCC 546
                   **********  ********************** ***** ********* *********
```

```
avrBs3               TTGCTCACGGTGGCGGGAGAGTTGAGAGGTCCACCGTTACAGTTGGACACAGGCCAACTT 780
bls256_tal2g         TTGCTCGCGGATGCGGGGGAGTTGAGAGGTCCGCCGTTACAGTTGGACACAGGCCAACTT 780
avrXa27              TTGCTCACGAAGGCGGGGGAGTTGAGAGGTCCGCCGTTACAGTTGGACACAGGCCAACTT 780
arp3                 TTGCTCACGAAGGCGGGGGAGTTGAGAGGTCCGCCGTTACAGTTGGACACAGGCCAACTT 606
pxo99a_tal3a         TTGCTCACGAAGGCGGGGGAGTTGAGAGGTCCGCCGTTACAGTTGGACACAGGCCAACTT 606
pxo86_tal3           TTGCTCACGAAGGCGGGGGAGTTGAGAGGTCCGCCGTTACAGTTGGACACAGGCCAACTT 606
bls256_tal2h         TTGCTCACGAAGGCGGGGGAGTTGAGAGGTCCGCCGTTACAGTTGGACACAGGCCAACTT 606
rs105_tal            TTGCTCACGAAGGCGGGGGAGTTGAGAGGTCCGCCGTTACAGTTGGACACAGGCCAACTT 606
jsb2-24_tal          TTGCTCACGAAGGCGGGGGAGTTGAGAGGTCCGCCGTTACAGTTGGACACAGGCCAACTT 606
pxo86_tal6           TTGCTCACGAAGGCGGGGGAGTTGAGAGGTCCGCCGTTACAGTTGGACACAGGCCAACTT 606
pxo99a_tal3b         TTGCTCACGAAGGCGGGGGAGTTGAGAGGTCCGCCGTTACAGTTGGACACAGGCCAACTT 606
maff311018_tal5      TTGCTCACGAAGGCGGGGGAGTTGAGAGGTCCGCCGTTACAGTTGGACACAGGCCAACTT 606
                     ****** **   ***** ************** ***************************

avrBs3               CTCAAGATTGCAAAACGTGGCGGCGTGACCGCAGTGGAGGCAGTGCATGCATGGCGCAAT 840
bls256_tal2g         CTCAAGATTGCAAAACGTGGCGGCGTGACCGCAGTGGAGGCAGTGCATGCATCGCGCAAT 840
avrXa27              CTCAAGATTGCAAAACGTGGCGGCGTGACCGCAGTGGAGGCAGTGCATGCATCGCGCAAT 840
arp3                 CTCAAGATTGCAAGACGTGGCGGCGTGACCGCAGTGGAGGCAGTGCATGCATGGCGCAAT 666
pxo99a_tal3a         CTCAAGATTGCAAGACGTGGCGGCGTGACCGCAGTGGAGGCAGTGCATGCATGGCGCAAT 666
pxo86_tal3           CTCAAGATTGCAAGACGTGGCGGCGTGACCGCAGTGGAGGCAGTGCATGCATGGCGCAAT 666
bls256_tal2h         CTCAAGATTGCAAGACGTGGCGGCGTGACCGCAGTGGAGGCAGTGCATGCATGGCGCAAT 666
rs105_tal            CTCAAGATTGCAAGACGTGGCGGCGTGACCGCAGTGGAGGCAGTGCATGCATGGCGCAAT 666
jsb2-24_tal          CTCAAGATTGCAAGACGTGGCGGCGTGACCGCAGTGGAGGCAGTGCATGCATGGCGCAAT 666
pxo86_tal6           CTCAAGATTGCAAGACGTGGCGGCGTGACCGCAGTGGAGGCAGTGCATGCATGGCGCAAT 666
pxo99a_tal3b         CTCAAGATTGCAAGACGTGGCGGCGTGACCGCAGTGGAGGCAGTGCATGCATGGCGCAAT 666
maff311018_tal5      CTCAAGATTGCAAGACGTGGCGGCGTGACCGCAGTGGAGGCAGTGCATGCATGGCGCAAT 666
                     ************* ************************************** *******

avrBs3               GCACTGACGGGTGCCCCCCTGAAC 864
bls256_tal2g         GCACTGACGGGTGCCCCCCTGAAC 864
avrXa27              GCACTGACGGGTGCCCCCCTGAAC 864
arp3                 GCACTGACGGGTGCCCCCCTGAAC 690
pxo99a_tal3a         GCACTGACGGGTGCCCCCCTGAAC 690
pxo86_tal3           GCACTGACGGGTGCCCCCCTGAAC 690
bls256_tal2h         GCACTGACGGGTGCCCCCCTGAAC 690
rs105_tal            GCACTGACGGGTGCCCCCCTGAAC 690
jsb2-24_tal          GCACTGACGGGTGCCCCCCTGAAC 690
pxo86_tal6           GCACTGACGGGTGCCCCCCTGAAC 690
pxo99a_tal3b         GCACTGACGGGTGCCCCCCTGAAC 690
maff311018_tal5      GCACTGACGGGTGCCCCCCTGAAC 690
                     ************************
```
