# Supplementary material for: Single molecule real-time sequencing of Xanthomonas oryzae genomes reveals a dynamic structure and complex TAL (transcription activator-like) effector gene relationships
Source: Microb Genom. 2015 Oct 30;1(4):e000032. doi: 10.1099/mgen.0.000032 (PMC4853030; doi:10.1099/mgen.0.000032)
Supplement: Supplementary file 9 — Supplementary Data [file mgen-01-32-s009.pdf]

Supplementary Material
for

SMRT SEQUENCING OF *XANTHOMONAS ORYZAE* GENOMES REVEALS A DYNAMIC STRUCTURE AND COMPLEX TAL EFFECTOR GENE RELATIONSHIPS

Nicholas J. Booher[1], Sara C. D. Carpenter[1], Robert P. Sebra[2], Li Wang[1], Steven L. Salzberg[3], Jan E. Leach[4], and Adam J. Bogdanove[1]*
Address: [1] Plant Pathology and Plant-Microbe Biology Section, School of Integrative Plant Science, Cornell University, Ithaca, NY 14853 USA; [2] Icahn Institute for Genomics and Multiscale Biology and Department of Genetics & Genomic Sciences, Icahn School of Medicine at Mount Sinai, New York, NY 10029 USA; [3] Departments of Biomedical Engineering, Computer Science, and Biostatistics and Center for Computational Biology, Johns Hopkins University, Baltimore, MD 21205 USA; [4] Bioagricultural Sciences and Pest Management, Colorado State University, Ft. Collins, CO 80523 USA
***Corresponding author: ajb7@cornell.edu**

File S9. The 5' end of *tal11g* of CFBP7342. Sequences of the 5' ends of the archetypal *X. euvesicatoria avrBs3* gene, the BLS256 *tal2g* gene (representing Xoc *tal* genes), and *avrXa27* (representing Xoo *tal* genes) are shown for comparison. Genbank accessions for sequences included in the alignment are given in the legend to File S6.

```
avrBs3          ATGGATCCCATTCGTTCGCGCACACCAAGTCCTGCCCGCGAGCTTCTGCCCGGACCCCAA 60
bls256_tal2g    ATGGATCCCATTCGTCCGCGCAGGCCAAGTCCTGCCCGCGAGCCTCTGCCCGGACCCCAA 60
avrXa27         ATGGATCCCATTCGTTCGCGCACGCCAAGTCCTGCCCGCGAGCTTCTGCCCGGACCCCAA 60
cfbp7342_tal11g ATGGATCCCATTCGTCCGCGCAGGCCAAGTCCTGCCCGCGAGCTTCTGCCCGGACCCCAA 60
                *************** ******  ******************* ****************

avrBs3          CCCGATGGGGTTCAGCCGACTGCAGATCGTGGGGTGT-CTCCGCCTGCCGGCGGCCCCCT 119
bls256_tal2g    CCGGATGGGGTTCAGCCGACTGCAGATCGGGGGGTGT-CTGCGCCTGCTGGCGGCCCTCT 119
avrXa27         CCGGATAGGGTTCAGCCGACTGCAGATCGGGGGGGGG-CTCCGCCTGCTGGCGGCCCCCT 119
cfbp7342_tal11g CCGGATAGGATTCAGCCGACTGCAGATCGGGGGGGGGGCTCCGCCTGCTGGCGGCCCCCT 120
                ** *** ** ******************* **** *   ** ******* ******** **

avrBs3          GGATGGCTTGCCCGCTCGGCGGACGATGTCCCGGACCCGGCTGCCATCTCCCCCTGCCCC 179
bls256_tal2g    GGATGGTTTGCCCGCTCGGCGGACGATGTCCCGGACCCGGCTGCCATCTCCCCCTGCGCC 179
avrXa27         GGATGGCTTGCCCGCTCGGCGGACGATGTCCCGGACCCGGCTGCCATCTCCCCCTGCGCC 179
cfbp7342_tal11g GGATGGCTTGCCCGCTCGGCGGACGATGTCCCGGACCCGGCTGCCATCTCCCCCTGCGCC 180
                ****** ************************************************** **

avrBs3          CTCACCTGCGTTCTCGGCGGGCAGCTTCAGTGACCTGTTACGTCAGTTCGATCCGTCACT 239
bls256_tal2g    CTCGCCTGCGTTCTCGGCGGGCAGCTTCAGCGATCTGCTCCGTCCGTTCGATCCGTCGCT 239
avrXa27         CTCGCCTGCGTTCTCGGCGGGCAGCTTCAACGATCTGCTCCGTCAGTTCGATCCGTCGCT 239
cfbp7342_tal11g CTCGCCTGCGTTCTCGGCGGGCAGCTTCAGCGATCTGCTCCGTCCGTTCGATCCGTCGCT 240
                *** *************************  ** *** * **** ************ **

avrBs3          T----------------------------------------------------------- 240
bls256_tal2g    T----------------------------------------------------------- 240
avrXa27         T----------------------------------------------------------- 240
cfbp7342_tal11g TCGACGTCCCCCCGAGTTTGAGTAGCACCTTGGTTTGGAGTCCAATTCCCTACCCCGAGG 300
                *

avrBs3          ------------------------------------------------------------
bls256_tal2g    ------------------------------------------------------------
```

```
avrXa27           ------------------------------------------------------------
cfbp7342_tal11g   AGATTGGACGTGAAGAAGCGTTTTTCCGAAGAACAGATCATCGGCTTCCTGCGCGAAGCC 360

avrBs3            ------------------------------------------------------------
bls256_tal2g      ------------------------------------------------------------
avrXa27           ------------------------------------------------------------
cfbp7342_tal11g   GAGGCCGGCATGCCGATCAAGGACCTGTGCCGGCGGCATGGCTTCAGTGAGGCCTCGTAC 420

avrBs3            ------------------------------------------------------------
bls256_tal2g      ------------------------------------------------------------
avrXa27           ------------------------------------------------------------
cfbp7342_tal11g   TACCTGTGGCGCAGTAAGTTCGGCGGCATGAGCGTGCCCGATGCCAAGCGGCTCAAGGAC 480

avrBs3            ------------------------------------------------------------
bls256_tal2g      ------------------------------------------------------------
avrXa27           ------------------------------------------------------------
cfbp7342_tal11g   CTGGAGGCCGAGAACACGCGGCTGAAGAAGTTGCTGGCCGAGCAGGTGTTCCAGAACGAC 540

avrBs3            ------------------------------------------------------------
bls256_tal2g      ------------------------------------------------------------
avrXa27           ------------------------------------------------------------
cfbp7342_tal11g   CTGATCAAGGATGCGCTGCAAAAAAAATGGTGAGCGCACCGGCGCGTCGTGCGCTGGTGC 600
```

```
avrBs3              ------------------------------------------------------------
bls256_tal2g        ------------------------------------------------------------
avrXa27             ------------------------------------------------------------
cfbp7342_tal11g     GCGAGTGGATCGAAGGTGGCGCCAGCGAGCGCTGCGCCCTGGCGGCGATCGGCATGAGCG 660

avrBs3              ------------------------------------------------------------
bls256_tal2g        ------------------------------------------------------------
avrXa27             ------------------------------------------------------------
cfbp7342_tal11g     CCAGTGCGCTGCGCTATCGCCCGCGCGAGGACCGCAACGTTGAGCTGCGCGAGCGCATCC 720

avrBs3              ------------------------------------------------------------
bls256_tal2g        ------------------------------------------------------------
avrXa27             ------------------------------------------------------------
cfbp7342_tal11g     TTGCGTTGGCGCATCGCCATCGCCGCTATGGCGTGGGGATGATCTATCTCAAGCTGCGGC 780

avrBs3              ------------------------------------------------------------
bls256_tal2g        ------------------------------------------------------------
avrXa27             ------------------------------------------------------------
cfbp7342_tal11g     AGGAAGGTCGTCTGGTGAACTATAAGCGGGTGGAGCGGCTGTATTGCGAGCAGCAGCTGC 840

avrBs3              ------------------------------------------------------------
bls256_tal2g        ------------------------------------------------------------
avrXa27             ------------------------------------------------------------
cfbp7342_tal11g     AGGTCCGCCGCCGCAAGCGCAAAAAGGTGCCGGTTGGCGAGCGTGCACCGTTGCTGCGGC 900

avrBs3              ------------------------------------------------------------
bls256_tal2g        ------------------------------------------------------------
avrXa27             ------------------------------------------------------------
cfbp7342_tal11g     CCACCAAGGCCAACCAGGTGTGGTCGATGGACTTCGTGTTCGACCGCACCGCCGAAGGTC 960

avrBs3              ------------------------------------------------------------
bls256_tal2g        ------------------------------------------------------------
avrXa27             ------------------------------------------------------------
cfbp7342_tal11g     GGGCGATCAAGTGTCTGGTGATCGTGGACGACGCAACCCACGAAGCGGTCGCCATCGAGG 1020

avrBs3              ------------------------------------------------------------
bls256_tal2g        ------------------------------------------------------------
avrXa27             ------------------------------------------------------------
cfbp7342_tal11g     TCGAACGCGCCATCTCCGGCCACGGCGTTGCGCGCGTGCTGGATCGGTTGGCACACAGTC 1080

avrBs3              ------------------------------------------------------------
bls256_tal2g        ------------------------------------------------------------
avrXa27             ------------------------------------------------------------
cfbp7342_tal11g     GCGGCCTGCCGAAGATGATCCGCACGGACAACGGCAAGGAGTTCTGTGGCAAGGCCATGG 1140

avrBs3              ------------------------------------------------------------
bls256_tal2g        ------------------------------------------------------------
avrXa27             ------------------------------------------------------------
cfbp7342_tal11g     TCGCCTGGGCGCATGCCAATCGTGTGCAGCTACGCCAGATCCAGCCTGGCAAGCCGAACC 1200
```

```
avrBs3              ------------------------------------------------------------
bls256_tal2g        ------------------------------------------------------------
avrXa27             ------------------------------------------------------------
cfbp7342_tal11g     AGAATGCCTATGTCGAATCCTTCAACGGCCGGCTGCGCGACGAATGCCTCAACGAACACT 1260


avrBs3              ------------------------------------------------------------
bls256_tal2g        ------------------------------------------------------------
avrXa27             ------------------------------------------------------------
cfbp7342_tal11g     GGTTCCCAACGCTGCTGCATGCGCGCACCGAGATCGAACGCTGGCGCCGCGAATACAACG 1320


avrBs3              ------------------------------------------------------------
bls256_tal2g        ------------------------------------------------------------
avrXa27             ------------------------------------------------------------
cfbp7342_tal11g     AGGACCGACCCAAGAAAGCAATCGGCGCAATGACGCCGGCGGCCTATGCCCAGCAGTTGG 1380


avrBs3              ------------------------------------------------------------
bls256_tal2g        ------------------------------------------------------------
avrXa27             ------------------------------------------------------------
cfbp7342_tal11g     CCAATAGCGATATCATCAACCCCGGACTCTAAACCCGACTGCTACTCAGGATGGGGGGAC 1440


avrBs3              --------TTTAATACATCGCTTTTTGATTCATTGCCTCCCTTCGGCGCTCACCATACAG 292
bls256_tal2g        --------CTTGATACATCGCTTCTTGATTCGATGCCTGCCGTCGGCACGCCGCATACAG 292
avrXa27             --------CTTGATACATCGCTTCTTGATTCGATGCCTGCCGTCGGCACGCCGCATACAG 292
cfbp7342_tal11g     GTCGGCTTCTTGATACATCGCTTCTTGATTCGATGCCTGCCGTCGGCACGCCGCATACAG 1500
                             ** *********** *******  ***** ** ***** * *  *******

avrBs3              AGGCTGCCACAGGCGAGTGGGATGAGGTGCAATCGGGTCTGCGGGCAGCCGACGCCCCCC 352
bls256_tal2g        CGGCTGCCCCAGCAGAGTGGGATGAGGCGCAATCGGGTCTGCGTGCAGCCGATGACCCGC 352
avrXa27             CGGCTGCCCCAGCAGAGTGGGATGAGGTGCAATCGGGTCTGCGTGCAGCCGATGACCCGC 352
cfbp7342_tal11g     CGGCTGCCCCAGCAGAGTGGGATGAGGTGCAATCGGCTCTGCGTGCAGCCGATGACCCGC 1560
                     ******* ***  ************* ******** ****** ******** * *** *

avrBs3              CACCCACCATGCGCGTGGCTGTCACTGCCGCGCGGCCGCCGCGCGCCAAGCCGGCGCCGC 412
bls256_tal2g        CACCCACCGTGCGTGTCGCTGTCACTGCCGCGCGGCCGCCGCGCGCCAAGCCGGCCCCGC 412
avrXa27             CACCCACCGTGCGTGTCGCTGTCACTGCCGCGCGGCCGCCGCGCGCCAAGCCGGCCCCGC 412
cfbp7342_tal11g     CACCCACCGTGCGTGTCGCTGTCACTGCCGCGCGGCCGCCGCGCGCCAAGCCGGCCCCGC 1620
                    ******** **** ** ************************************** ****

avrBs3              GACGACGTGCTGCGCAACCCTCCGACGCTTCGCCGGCCGCGCAGGTGGATCTACGCACGC 472
bls256_tal2g        GACGGCGTGCGGCGCAACCCTCCGACGCTTCGCCGGCCGCGCAGGTGGATCTAAGCACGC 472
avrXa27             GACGGCGTGCGGCGCAACCCTCCGACGCTTCGCCGGCCGCGCAGGTGGATCTACGCACGC 472
cfbp7342_tal11g     GACGGCGTGCGGCGCAACCCTCCGACGCTTCGCCGGCCGCGCAGGTGGATCTACGCACGC 1680
                    **** ***** ****************************************** ******

avrBs3              TCGGCTACAGCCAGCAGCAACAGGAGAAGATCAAACCGAAGGTTCGTTCGACAGTGGCGC 532
bls256_tal2g        TCGGCTACAGTCAGCAGCAGCAAGAGAAGATCAAACCGAATGTGCGTTCGACAGTGGCGC 532
avrXa27             TCGGCTACAGTCAGCAGCAGCAAGAGAAGATCAAATCGAAGGTGCGTTCGACAGTGGCGC 532
cfbp7342_tal11g     TCGGCTACAGTCAGCAGCAGCAAGAGAAGATCGAACCGAATGTTCGTTCGACAGTTGCGC 1740
                    ********** ******** ** ********* ** **** ** *********** ****

avrBs3              AGCACCACGAGGCACTGGTCGGCCATGGGTTTACACACGCGCACATCGTTGCGCTCAGCC 592
bls256_tal2g        AGCACCACGAGGCACTGGTGGGCCATGGGTTTACACACGCGCACATCGTTGCGCTCAGCC 592
avrXa27             AGCACCACGAGGCACTGGTGGGCCATGGGTTTACACACGCGCACATCGTTGCGCTCAGCC 592
cfbp7342_tal11g     AGCACCACGAGGCACTGGTGGGCCATGGGTTTACACACGCGCACATCGTTGCGCTCAGCC 1800
                    ******************* ****************************************
```

```
avrBs3              AACACCCGGCAGCGTTAGGGACCGTCGCTGTCAAGTATCAGGACATGATCGCAGCGTTGC 652
bls256_tal2g        AACACCCGGCAGCGTTAGGGACCGTTGCTGTCACGTATCAGCACATAATCACGGCGTTGC 652
avrXa27             AACACCCGGCAGCGTTAGGGACCGTCGCTGTCAAGTATCAGCACATAATCACGGCGTTGC 652
cfbp7342_tal11g     GACACCCGGCAGCGTTAGGGACCGTCGCTGTCACGTATCAAGACATAATCACGGCGTTGC 1860
                     ************************ ******* ******  **** *** * *******

avrBs3              CAGAGGCGACACACGAAGCGATCGTTGGCGTCGGCAAACAGTGGTCCGGCGCACGCGCTC 712
bls256_tal2g        CAGAGGCGACACACGAAGACATCGTTGGCGTCGGCAAACAGTGGTCCGGCGCACGCGCCC 712
avrXa27             CAGAGGCGACACACGAAGACATCGTTGGCGTCGGCAAACAGTGGTCCGGCGCACGCGCCC 712
cfbp7342_tal11g     CAGAGGCGACACACGAAGACATCGTTGGCGTCGGCAAACAGTGGTCCGGCGCACGCGCCC 1920
                    ******************  ************************************** *

avrBs3              TGGAGGCCTTGCTCACGGTGGCGGGAGAGTTGAGAGGTCCACCGTTACAGTTGGACACAG 772
bls256_tal2g        TGGAGGCCTTGCTCGCGGATGCGGGGGAGTTGAGAGGTCCGCCGTTACAGTTGGACACAG 772
avrXa27             TGGAGGCCTTGCTCACGAAGGCGGGGGAGTTGAGAGGTCCGCCGTTACAGTTGGACACAG 772
cfbp7342_tal11g     TGGAGGCCTTGCTCACGGTGGCGGGAGAGTTGAGAGGTCCACCGTTGCAGTTGGACACAG 1980
                    ************** **   ***** ************** ***** *************

avrBs3              GCCAACTTCTCAAGATTGCAAAACGTGGCGGCGTGACCGCAGTGGAGGCAGTGCATGCAT 832
bls256_tal2g        GCCAACTTCTCAAGATTGCAAAACGTGGCGGCGTGACCGCAGTGGAGGCAGTGCATGCAT 832
avrXa27             GCCAACTTCTCAAGATTGCAAAACGTGGCGGCGTGACCGCAGTGGAGGCAGTGCATGCAT 832
cfbp7342_tal11g     GCCAACTTGTCAAGATTGCAAAACGTGGCGGCGTGACCGCAGTGGAGGCAGTGCATGCAT 2040
                    ******** ***************************************************

avrBs3              GGCGCAATGCACTGACGGGTGCCCCCCTGAAC 864
bls256_tal2g        CGCGCAATGCACTGACGGGTGCCCCCCTGAAC 864
avrXa27             CGCGCAATGCACTGACGGGTGCCCCCCTGAAC 864
cfbp7342_tal11g     CGCGCAATGCACTGACGGGTGCCCCCCTGAAC 2072
                     *******************************
```
