# Supplementary material for: Single molecule real-time sequencing of Xanthomonas oryzae genomes reveals a dynamic structure and complex TAL (transcription activator-like) effector gene relationships
Source: Microb Genom. 2015 Oct 30;1(4):e000032. doi: 10.1099/mgen.0.000032 (PMC4853030; doi:10.1099/mgen.0.000032)
Supplement: Supplementary file 10 — Supplementary Data [file mgen-01-32-s010.pdf]

Supplementary Material
for

SMRT SEQUENCING OF *XANTHOMONAS ORYZAE* GENOMES REVEALS A DYNAMIC STRUCTURE AND COMPLEX TAL EFFECTOR GENE RELATIONSHIPS

Nicholas J. Booher[1], Sara C. D. Carpenter[1], Robert P. Sebra[2], Li Wang[1], Steven L. Salzberg[3], Jan E. Leach[4], and Adam J. Bogdanove[1]*
Address: [1] Plant Pathology and Plant-Microbe Biology Section, School of Integrative Plant Science, Cornell University, Ithaca, NY 14853 USA; [2] Icahn Institute for Genomics and Multiscale Biology and Department of Genetics & Genomic Sciences, Icahn School of Medicine at Mount Sinai, New York, NY 10029 USA; [3] Departments of Biomedical Engineering, Computer Science, and Biostatistics and Center for Computational Biology, Johns Hopkins University, Baltimore, MD 21205 USA; [4] Bioagricultural Sciences and Pest Management, Colorado State University, Ft. Collins, CO 80523 USA
***Corresponding author: ajb7@cornell.edu**

File S10. Detailed discussion of *tal* gene relationships across strains.

Consistent with our previous comparison of the Sanger assemblies for Xoo strains PXO99A and MAFF311018 (Salzberg *et al.*, 2008), whole genome alignments of the BLS256 and CFBP7342 genomes and the PXO99A and PXO86 genomes show numerous small indels, some large transpositions, and several major inversions (Fig. 5). Within the pathovars, major clusters of *tal* genes appear largely conserved with regard to genomic context, orientation, and overall size, but only to a lesser extent gene content. Indeed, the content variation in a cluster across genomes within a pathovar and the presence of the same *tal* gene in different clusters in different genomes within and across pathovars suggests that for *tal* genes individually, recombination in and between clusters, integration into any cluster following horizontal transfer, and duplication followed by divergence are not infrequent events.

A striking example of apparent recombination is the relationship among *tal3a* of MAFF311018, *tal5a* of PXO86, and *tal5a* of PXO99A. Each has the same 5 codon deletion in the 5' end, and the PXO86 and PXO99A genes have identical encoded RVD sequences, but the MAFF311018 gene encodes a different RVD sequence, nearly identical instead to that of *tal7a* of PXO86 and *tal4* of PXO99A. The most likely explanation is that somewhere in the lineage that gave rise to these strains, two *tal* genes recombined to swap 5' ends (or CRRs). Recombination involving CRRs appears to be particularly frequent, or is more readily detected. Inspection of RVD sequences within and across the genomes (Fig. 7, Fig. 8, and (Bogdanove *et al.*, 2011; Ochiai *et al.*, 2005; Salzberg *et al.*, 2008)) yields several examples of CRRs that share a long stretch of RVDs flanked or in some cases interrupted by other RVDs not in common. In other cases, otherwise identical CRRs differ at only one or a few RVDs, or in the case of *tal6c* of MAFF311018 and *tal1* of PXO86, by the presence or absence of an atypical-length repeat.

Whereas such relationships might be expected to arise from the accumulation of point mutations and small indels, micro-recombination within a CRR likely also contributes.

Comparison of *tal* genes in the Xoc genomes with those in the Xoo genomes provides compelling evidence of horizontal gene transfer. As we noted previously (Bogdanove *et al.*, 2011), the 129 bp 3' end of *tal3b* of PXO99A and sequences surrounding it are typical of a variant found in BLS256 (*tal2h*, Fig. 6), suggesting transfer to Xoo from Xoc. This atypical 3' end is present also in one *tal* gene each in the MAFF311018 and PXO86 genomes and in the CFBP7342 genome. The three Xoo genes share similar RVD sequences. These are different from those of the BLS256 and CFBP7342 genes, which unlike the Xoo genes are distinct from one another. This conservation in Xoo and divergence in Xoc of the CRRs associated with the 129 bp 3' variant bolster the conclusion that the transfer was from Xoc to Xoo and that it took place prior to the divergence of the Xoo strains. Other possibilities exist, however, including horizontal transfer among the Xoo strains, or persistence of the variant from the common ancestor of Xoc and Xoo. In addition to the 129 bp 3' end variant, an 11 codon duplication found in the 3' end of *tal2f* of BLS256 is another example of evidence for horizontal transfer. This same duplication is present in some of the Xoo *tal* genes (Fig. 6). Its absence from CFBP7342 argues against it having been transmitted vertically from the common ancestor. The duplication appears to have been integrated intragenically following transfer: after the 11 codon duplication, *tal2f* ends at the typical point for an Xoc *tal* gene while the Xoo *tal* genes with the duplication lack that stop codon and end 24 bases later at the typical Xoo location. A third example of probable horizontal transfer is *tal5a* of BLS256, which aligns with *tal2a* of MAFF311018, *tal8e* PXO86, and *tal9e* of PXO99A, differing only at codons for two RVDs and in the extreme 3' ends, which are typical of the respective pathovars, suggesting integration by recombination on transfer. A final example is found in *tal3* of PXO86 and *tal3a* of PXO99A, which each bear a premature stop codon. Following that stop codon the sequence in each case aligns closely with a typical Xoc *tal* gene 3' end, and the next stop codon occurs at the typical Xoc location. As noted in Results, *tal3* and *tal6* of PXO86 arose from a 13.5 kb duplication that also gave rise to *tal3a* and *tal3b* in PXO99A. *tal6* of PXO86 and *tal3b* of PXO99A, along with *tal5* of MAFF311018, share similar 3' ends distinct from the Xoc-like end in *tal3* of PXO86 and *tal3a* of PXO99a. Thus, the Xoc-like end is likely another example of an intragenically integrated horizontal transfer, one in this case also associated with a gene duplication event.

In addition to *tal3* and *tal6* of PXO86, two other examples provide evidence of duplications contributing to the evolution of *tal* gene content. First, as detailed in Results, we discovered that the 212 kb duplication in the PXO99A lineage gave rise to a *tal* gene variant with a deletion of 15 repeats from its CRR, and that distinct reversions took place in subsequent culture that resulted in genotypes with either the original full length *tal* gene or the new short variant. Second, the 3' end variant represented by *tal2f* of BLS256, discussed above as an example of likely horizontal transfer, is present in one *tal* gene in MAFF311018, but in two each in the other Xoo genomes (yellow arrowheads in Fig. 6). Within those genomes, the two genes have distinct RVD sequences, suggesting differentiation following a duplication.

Overall, the dynamic genome structure and complex *tal* gene relationships that the comparisons reveal illustrate the highly plastic nature of *tal* gene content in this species.

References

Bogdanove, A. J., Koebnik, R., Lu, H., Furutani, A., Angiuoli, S. V., Patil, P. B., Van Sluys, M. A., Ryan, R. P., Meyer, D. F. & other authors (2011). Two new complete genome

sequences offer insight into host and tissue specificity of plant pathogenic *Xanthomonas spp. J Bacteriol* **193**, 5450-5464.

Ochiai, H., Inoue, Y., Takeya, M., Sasaki, A. & Kaku, H. (2005). Genome sequence of *Xanthomonas oryzae* pv. oryzae suggests contribution of large numbers of effector genes and insertion sequences to its race diversity. *JARQ-Jpn Agr Res Q* **39**, 275-287.

Salzberg, S. L., Sommer, D. D., Schatz, M. C., Phillippy, A. M., Rabinowicz, P. D., Tsuge, S., Furutani, A., Ochiai, H., Delcher, A. L. & other authors (2008). Genome sequence and rapid evolution of the rice pathogen *Xanthomonas oryzae* pv. oryzae PXO99A. *BMC Genomics* **9**, 204.
