# Supplementary material for: Single molecule real-time sequencing of Xanthomonas oryzae genomes reveals a dynamic structure and complex TAL (transcription activator-like) effector gene relationships
Source: Microb Genom. 2015 Oct 30;1(4):e000032. doi: 10.1099/mgen.0.000032 (PMC4853030; doi:10.1099/mgen.0.000032)
Supplement: Supplementary file 11 — Supplementary Data [file mgen-01-32-s011.pdf]

Supplementary Material
for

SMRT SEQUENCING OF *XANTHOMONAS ORYZAE* GENOMES REVEALS A DYNAMIC STRUCTURE AND COMPLEX TAL EFFECTOR GENE RELATIONSHIPS

Nicholas J. Booher[1], Sara C. D. Carpenter[1], Robert P. Sebra[2], Li Wang[1], Steven L. Salzberg[3], Jan E. Leach[4], and Adam J. Bogdanove[1]*
Address: [1] Plant Pathology and Plant-Microbe Biology Section, School of Integrative Plant Science, Cornell University, Ithaca, NY 14853 USA; [2] Icahn Institute for Genomics and Multiscale Biology and Department of Genetics & Genomic Sciences, Icahn School of Medicine at Mount Sinai, New York, NY 10029 USA; [3] Departments of Biomedical Engineering, Computer Science, and Biostatistics and Center for Computational Biology, Johns Hopkins University, Baltimore, MD 21205 USA; [4] Bioagricultural Sciences and Pest Management, Colorado State University, Ft. Collins, CO 80523 USA
***Corresponding author: ajb7@cornell.edu**

File S11. Settings and procedure for assembly with HGAP v3.0.

Software
PacBio data processing and analysis were done using SMRTAnalysis 2.2. The BLASR aligner included in the distribution was updated to revision 133977 from the GitHub repository to fix an issue of incorrect mapQVs given to alignments resulting in poor assemblies.

Whole genome assembly with HGAP v3.0
HGAP v3.0 was run with the following settings. For the filter stage of the protocol the minimum read and minimum subread length cutoffs were set to 4000 to ensure that any reads containing a *tal* gene repeat region would be unambiguously alignable. For the assembly stage the target genome size was set to 5000000 to reflect the approximate size of previously sequenced *Xanthomonas* genomes, and the BLASR options string was set to “-noSplitSubreads -minReadLength 4000 -minSubreadLength 4000 -maxScore -1000 -maxLCPLength 16”. For the mapping stage, the “Place Repeats Randomly” option was unchecked, and the pbalign_opts setting string was set to “--seed=1 --minAccuracy=0.75 --minLength=50 --algorithmOptions=‘-useQuality -minReadLength 4000 -minSubreadLength 4000’”. All other settings were left at defaults. After the run, for each strain the polished assembly was run through the RS_Resequencing protocol again with filter and mapping settings set to those used for the HGAP v3.0 run. The consensus sequence of this run was circularized by splitting it in half at an arbitrary location away from any *tal* gene region and assembling the fragments with Minimo, and the assembled sequence was then rotated and flipped to match the start position and strand of the start of the reference sequence. The RS_Resequencing protocol was then run again with the earlier settings to produce the final assembly.
